# Prevalence of common mental disorders among medical students in China: a systematic review and meta-analysis

**DOI:** 10.3389/fpubh.2023.1116616

**Published:** 2023-08-31

**Authors:** Jinxingyi Wang, Min Liu, Jian Bai, Yuhan Chen, Jie Xia, Baolin Liang, Ruixuan Wei, Jiayin Lin, Jiajun Wu, Peng Xiong

**Affiliations:** ^1^The Second Affiliated Hospital of Guizhou Medical University, Guizhou, China; ^2^Zhuhai Center for Maternal and Child Health Care, Zhuhai Women and Children's Hospital, Zhuhai, China; ^3^School of Medicine, Jinan University, Guangzhou, China; ^4^Department of Public Health and Preventive Medicine, School of Medicine, Jinan University, Guangzhou, China; ^5^School of Stomatology, Jinan University, Guangzhou, China; ^6^School of Nursing, Jinan University, Guangzhou, China

**Keywords:** common mental disorders (CMDs), depression, anxiety, suicidal behaviors, medical students, meta-analysis

## Abstract

**Background:**

The prevalence of mental distress is common for medical students in China due to factors such as the long duration of schooling, stressful doctor-patient relationship, numerous patient population, and limited medical resources. However, previous studies have failed to provide a comprehensive prevalence of these mental disorders in this population. This meta-analysis aimed to estimate the prevalence of common mental disorders (CMDs), including depression, anxiety, and suicidal behaviors, among medical students in China.

**Methods:**

We conducted a systematic search for empirical studies on the prevalence of depression, anxiety, suicide attempt, suicide ideation, and suicide plan in Chinese medical students published from January 2000 to December 2020. All data were collected pre-COVID-19. The prevalence and heterogeneity estimations were computed by using a random-effects model and univariate meta-regression analyses.

**Results:**

A total of 197 studies conducted in 23 provinces in China were included in the final meta-analysis. The prevalence data of depression, anxiety, suicide attempt, suicide ideation, and suicide plan were extracted from 129, 80, 21, 53, and 14 studies, respectively. The overall pooled crude prevalence for depression was 29% [38,309/132,343; 95% confidence interval (CI): 26%−32%]; anxiety, 18% (19,479/105,397; 95% CI: 15%−20%); suicide ideation, 13% (15,546/119,069; 95% CI: 11%−15%); suicide attempt, 3% (1,730/69,786; 95% CI: 1%−4%); and suicide plan, 4% (1,188/27,025; 95% CI: 3%−6%).

**Conclusion:**

This meta-analysis demonstrated the high prevalence of CMDs among Chinese medical students. Further research is needed to identify targeted strategies to improve the mental health of this population.

## Introduction

Worldwide, medical schools aim to train and produce competent medical doctors to meet healthcare needs and promote public health. This is achieved through arduous training that requires high motivation, intelligence, and endurance. Globally, medical students usually experience high-pressure situations during school, such as the long duration of training (1), the heavy workload of intern clinical practice (2), sleep deprivation (3), financial concerns (4), intensive exams, and career uncertainty (5). Such pressures could cause negative effects on medical students' wellbeing (6) and academic performance (7) and precipitate mental distresses such as depression, anxiety symptoms, and suicidal behaviors (8, 9). A systematic review and meta-analysis including 167 cross-sectional empirical studies reported a global prevalence of depression or depressive symptoms and suicidal ideation in medical students of 27.2 and 11.1%, respectively, indicating high psychological morbidities in this population (10). Furthermore, a meta-analysis involving 57 studies (*n* = 25,735) demonstrated a substantial prevalence of poor sleep quality of 52.7% among medical students worldwide (11). Burnout among medical students is common as well. A systematic review of 58 studies reported a wide range of burnout prevalence, varying from 7.0 to 75.2% (12). Even before entering residency, the burden of burnout is substantial, as demonstrated by a meta-analysis encompassing 17,431 medical students, which found that 44.2% of global medical students experienced burnout, regardless of gender (13). Anxiety is another significant concern affecting medical students, with a substantially higher prevalence compared to the general population. Globally, about one in three (33.8%) medical students experience anxiety, with a higher prevalence observed among medical students from the Middle East and Asia (14). Furthermore, as medical students advance to higher levels of training and enter residency, they continue to face a significant risk of experiencing mental distress. A meta-analysis that incorporated data from 31 cross-sectional and 23 longitudinal studies revealed an overall pooled prevalence of depression or depressive symptoms of 28.8% among resident physicians (15). Moreover, another meta-analysis involving 22,778 residents indicated that the prevalence of burnout was 51.0% (16). This further highlighted the enduring vulnerability of resident physicians to mental health challenges.

Undetected or untreated mental distress can have persistent and worsening effects, particularly for medical students (17). These effects can manifest in various adverse outcomes, including poor academic performance, a higher dropout rate, limited professional development (18), and impaired quality of life (19). Additionally, there is an increased risk of engaging in unhealthy coping mechanisms such as alcohol and substance abuse, as well as an elevated risk of suicide (20). Furthermore, the presence of chronic psychological distress among medical students can contribute to a decline in empathy and enthusiasm toward patients, resulting in higher rates of medical errors and increased levels of job burnout in future clinical practice (21). This, in turn, can further strain the doctor-patient relationship, diminish treatment quality (22), and ultimately impact the overall culture of the medical profession (20). It highlights the urgency of addressing mental health issues among medical students to prevent these detrimental consequences and ensure the wellbeing of both students and the patients they will serve in their future medical careers.

In China, the medical education system and healthcare environment differ in certain areas compared to Western or other Asian countries. China has great complexity in the levels of programs designed to train doctors. The main current medical education system in China comprises a 3-year junior college medical program, a 5-year medical bachelor's degree program, a “5 + 3” medical master's degree program, and an 8-year medical doctoral degree program (23). Usually, medical students have to go through the “5 + 3” model before gaining the formal job of a medical doctor. One type of “5 + 3” model is finishing 5 years of undergraduate medical education first (leading to a bachelor's degree), then completing 3 years of standardized residency training (SRT). The other type of “5 + 3” model encompasses 5 years of undergraduate education, the postgraduate entrance examination, and 3 years of a professional master's degree (master of medicine, MM) program (including SRT) (24). However, with the increasing demands and expectations of society and the medical system for doctors, more and more medical students choose to achieve a doctoral degree. The long medical schooling cycle that the medical students have to go through is undoubtedly a substantial burden for them. The numerous patient populations and relatively limited medical resources cause overwhelming workload pressures, which could further lead to burnout and low wellbeing (5). Recently, more stressful doctor-patient relationships for Chinese doctors in work settings (25) have been common. This unstable relationship frequently led to workplace violence, and with the patients as perpetrators, healthcare workers experienced greater physical and mental health burdens. These factors are likely to contribute to depression, anxiety symptoms, and suicidal behaviors (e.g., suicidal ideation).

The above findings warrant broader awareness of and greater attention to medical students' mental health in China. Previous meta-analyses have reported the pooled prevalence of mental distress in this population; however, some study limitations exist. For example, a meta-analysis of Chinese medical students published in 2019 and including 21 empirical studies demonstrated a mean prevalence of depression and anxiety of 32.74 and 27.22%, respectively (26). However, this study only investigated psychological morbidities in undergraduate medical students, excluding those at the graduate levels, who might bear a higher burden of mental distress due to higher academic pressure and challenging working environments (27). Another review with 10 primary studies reported the pooled prevalence of depression, anxiety, and suicidal ideation as 29%, 21%, and 11%, respectively (28). However, the review did not provide a comprehensive analysis of prevalence in this population in China because it failed to search related articles in Chinese databases. A recent systematic review and meta-analysis showed a 27% comprehensive prevalence of depression in Chinese medical students (29), but reported only the pooled estimate of one mental disease, i.e., depression, which failed to provide an overview of CMDs in this population.

Given this serious public health problem and the limitations of previous reviews, we aimed to perform a systematic review and meta-analysis by conducting a systematic search of English and Chinese databases to (1) systematically assess the comprehensive prevalence of common mental distresses (including depression, anxiety, suicide attempt, suicide ideation, and suicide plan) among medical students in China; (2) conduct subgroup analysis; and (3) explore the sources of heterogeneity among studies.

## Materials and methods

This meta-analysis was conducted in accordance with the standards of the Preferred Reporting Items for Systematic Reviews and Meta-Analyses (PRISMA) Statement (30) and the Meta-Analyses Observational Studies in Epidemiology (MOOSE) guidelines (31). This study was registered with the International Prospective Register of Systematic Reviews (PROSPERO) (CRD42019142527).

### Search strategy and study eligibility

An electronic search was conducted to identify original articles published from January 2000 to December 2020 that reported the prevalence of depression, anxiety, and suicidal behaviors (including suicide attempt, suicide ideation, and suicide plan) in Chinese medical students. Databases searched included PubMed, Cochrane Library, Cumulative Index to Nursing and Allied Health Literature (CINAHL), MEDLINE, PsycINFO, and the Chinese databases such as China National Knowledge Infrastructure [CNKI], WANFANG Data, and Weipu (CQVIP) Data. The key terms were “common mental disorders,” “depression,” “anxiety,” “suicide,” and “Chinese medical students.” The detailed search strategy is provided in the Supplementary material. Due to COVID-19, we did not include articles published after January 2021.

### Inclusion and exclusion criteria

Studies were included in this meta-analysis if they (1) reported original quantitative studies, including cross-sectional, cohort, and case-control studies; (2) were published in peer-reviewed journals; (3) were written in English or Chinese language; (4) reported on the population comprised of medical students in China (including Hong Kong, Macao, and Taiwan); and (5) used validated assessment tools with good reliability and validity to evaluate the level of depression, anxiety, and suicidal behaviors among medical students.

Studies were excluded if the (1) prevalence data could not be extracted by indirect calculation or by contacting the corresponding author; (2) publication format was a conference abstract, review, meta-analysis, export opinion, or letter; (3) reported sample size was <30 individuals; (4) the reported participants were not from China; (5) reported population was non-medical students; and (6) reported mental health problems arose under emergency or special circumstances, such as severe acute respiratory syndromes (SARS), Wenchuan earthquakes, and COVID-19.

### Selection procedure and data extraction

**Figure 1:**
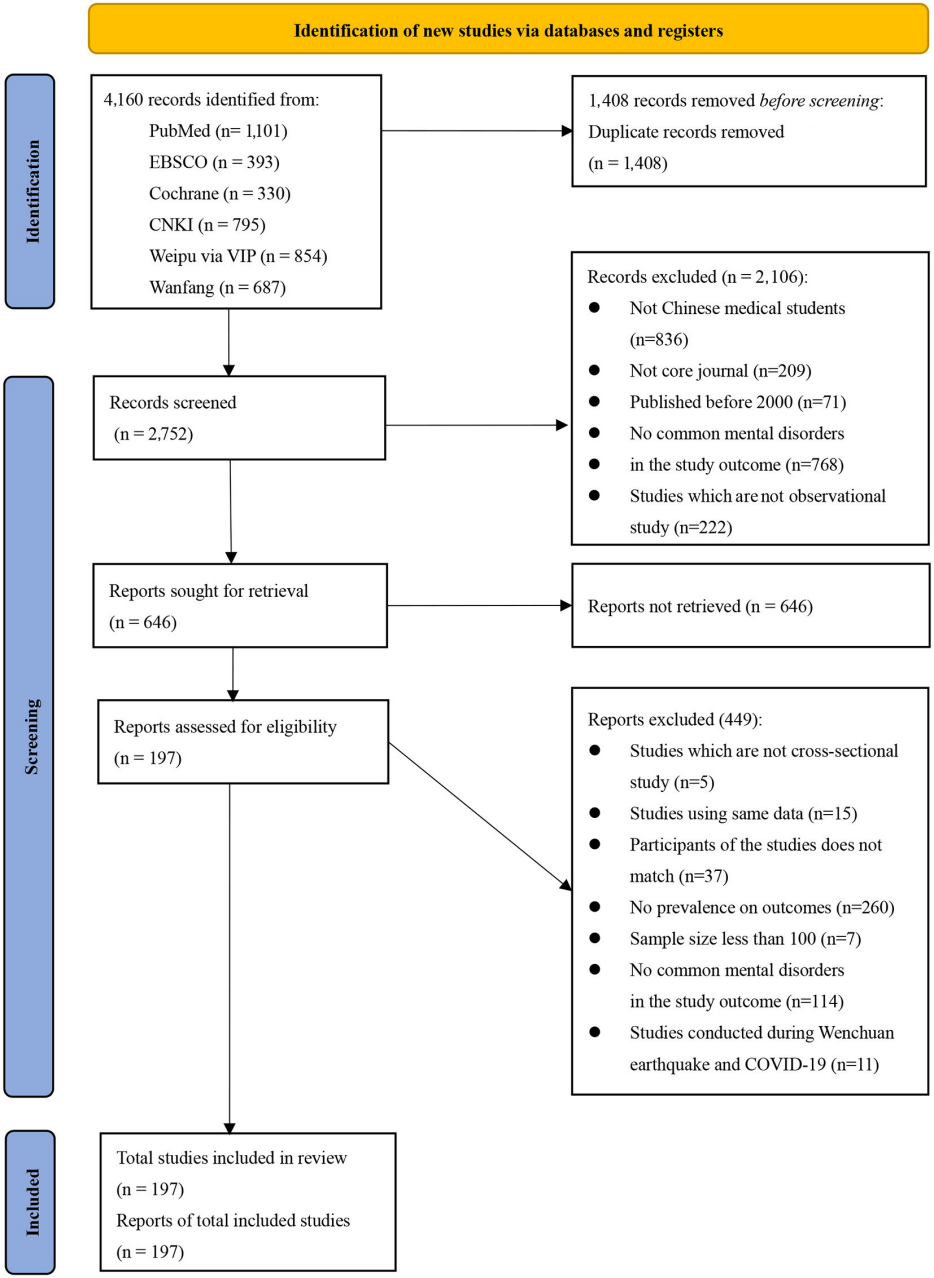
PRISMA flow chart for study selection.

First, two reviewers (JW and JB) independently identified and screened the articles by title and abstract to determine their eligibility for further examination. Then, the full texts were assessed against eligibility criteria independently by two reviewers (JW and JB), and any disagreement was resolved by a third reviewer (ML or PX; Figure 1). Finally, two reviewers (JW and JB) conducted data extraction from the final included studies. The extracted data included first author, year of publication, study location, sampling method, recall period, measurement tool and cutoff score, study type, sample size, number of medical students with mental problems (including depression, anxiety, and suicide attempt/ideation/plans), and sample characteristics (including age, grade, sex, school type, and major category).

### Quality appraisal

The quality appraisal was conducted independently by JW and JB using the Joanna Briggs Institute (JBI) Critical Appraisal Quality Assessment Tool (32). The tool was validated well and was popularly used in previous studies (33, 34). JBI is a renowned and efficient quality tool for assessing the credibility, relevance, and outcomes of prevalence studies. It is composed of 10 items, with each item scored from 0 to 2. A score of 0 represents “not mentioned,” 1 represents “mentioned but not described in detail,” and 2 represents “detailed and comprehensive description.” The higher the total score, the better the quality of the study in terms of credibility, relevance, and outcomes. The detailed scores of each included study are shown in the Supplementary material.

### Data synthesis and analysis

The pooled prevalence estimates of depression, anxiety, and suicidal behaviors were calculated by using random-effects models, which were applied when differences in study designs and methodology were assumed to produce variations in effect sizes across individual studies. The *Q*-statistic was used to evaluate the heterogeneity of effect sizes across studies, and a significant *p*-value indicated meaningful heterogeneity (35). The *I*^2^ statistic, a variance ratio, which described the proportion of heterogeneity observed in the total variability attributed to the heterogeneity between the studies and not to chance, was calculated (36). *I*^2^ values of 25%, 50%, and 75% indicated low, middle, and high levels of heterogeneity, respectively. To further explore the possible sources of heterogeneity, subgroup analysis and univariate meta-regression analysis were performed based on the following characteristics: study region, survey year, sample size, sampling method, recall period of suicidality, measurement tool, and cutoff score. Specifically, the regional classification was based on China's geographic divisions, including North China, East China, South China, Central China, Northeast China, Northwest China, Southwest China, and others (such as multiple regions and not reported). Sensitivity analyses were performed by serially excluding each study to determine the influence of individual studies on the overall prevalence estimates. Egger's test (37) and Begg's test (38) were utilized to investigate publication bias, with *p* < 0.05 demonstrating statistical publication bias. All statistical analyses were performed using the Stata software (version 14.2; StataCorp, College Station, TX, United States) (39).

## Results

### Characteristics of the included studies

A total of 197 studies involving 294,408 medical students in China were included in the final meta-analysis (Figure 1). The median sample size was 690 (range: 100–10,344). Among the included studies, 129 reported the prevalence of depression, with a combined sample size of 132,343 individuals. The prevalence of anxiety symptoms was reported in 80 studies, with a combined sample size of 105,397 individuals. The prevalence of suicide attempt, suicide ideation, and suicide plan was reported in 21, 53, and 14 studies, respectively, with combined samples of 69,786, 119,069, and 27,025 individuals.

Of the included studies, 172 were written in Chinese and 26 were written in English. A cross-sectional design was used in 197 studies, and only one study used a randomized controlled trial design. The JBI quality score of the 197 included studies ranged from 6 to 20, with a mean score of 15.

Publication years ranged from 2000 to 2020, and the study regions covered 23 provinces on the mainland and Taiwan Province of China. The most common sampling methods used were multiple sampling methods (*n* = 58), cluster sampling (*n* = 55), and simple random sampling (*n* = 44). Other methods, such as convenience sampling, stratified sampling, and multi-stage sampling, were also used in some of the included studies. With regard to measurement tools or items, 17, 13, and 19 types of tools were used to assess depression, anxiety symptoms, and suicidal behaviors (including suicide attempt, suicide ideation, and suicide plan), respectively. Common measurement tools for depression were Zung's Self-Rating Depression Scale (SDS), the Center for Epidemiologic Studies Depression Scale (CES-D), and the Beck Depression Rating Scale (BDI), which were used in 66, 17, and 17 of the included studies, respectively. Anxiety measurement tools were the Self-Rating Anxiety Scale (SAS), the symptom checklist-90 (SCL-90), and the Beck Anxiety Inventory (BAI), used in 52, 10, and 5 of the included studies, respectively. The assessments used for suicidal behaviors were self-made questionnaires or standardized scales, such as the National Comorbidity Survey (NCS) and Suicidal Behaviors Questionnaire (SBQ). The recall period to measure suicidal behavior included “past 1 week,” “past 6 months,” “past 1 year,” “past 2 years,” and “lifetime.” A detailed summary of the characteristics of the included studies is provided in Tables 1–3.

**Table 1:** Characteristics of the 129 studies included on depression in this review.

**Year**	**First author**	**Province**	**Age, years**	**Major**	**Grade**	**Sampling method**	**Measurement tools and cutoff score**	**Study type**
2000	Lin Daxi	Fujian	Mean: 19	Medicine	College students	Cluster sampling	SDS	Cross-sectional study
2000	Du Zhaoyun	Shandong	Mean (SD): 20.4 (1.6)	Medicine	Undergraduates	Simple random sampling and cluster sampling	BDI-13	Cross-sectional study
2000	Wu Hualin	Shanxi	Mean: 20.5	Medicine	College students	Simple random sampling	SDS	Cross-sectional study
2000	Yang Benfu	NA	Mean: 20.5	Medicine	Undergraduates	Stratified and cluster sampling	SDS	Cross-sectional study
2001	Yu Miao	Fujian	Mean: 21	Medicine	Undergraduates	Cluster sampling	CES-D	Cross-sectional study
2001	Lin Zhiping	Fujian	Mean: 21.5	Medicine	Undergraduates	Cluster sampling	CES-D	Cross-sectional study
2001	Zhang Yushan	Anhui	Mean (SD): 21.8 (3.2)	Medicine	Undergraduates	NA	SDS	Cross-sectional study
2001	Zhang Yunsheng	Henan	NA	Pharmacy and nursing	Undergraduates	Simple random sampling	SCL-90	Cross-sectional study
2002	Rao Hong	NA	Mean: 20	Medicine	College students	NA	BDI	Cross-sectional study
2002	Xu Limei	NA	Mean: 19	Medicine	Undergraduates	Stratified and cluster sampling	SDS	Cross-sectional study
2003	Zhou Rong	Guangdong	Mean: 21	Medicine	Undergraduates	Simple random sampling	SDS	Cross-sectional study
2003	Wang Menglong	Guangdong	Mean: 20	Medicine	Grades 1 and 3	NA	SDS	Cross-sectional study
2003	Gesang Zeren	NA	Mean: 16.5	Medicine and nursing	NA	NA	CES-D	Cross-sectional study
2004	Zhang Fuquan	Hunan	Mean (SD): 19.85 (1.18)	Medicine	Undergraduates	Stratified and cluster sampling	SCL-90	Cross-sectional study
2004	Zhang Shuying	NA	Mean (SD): 21.8 (0.89)	Medicine	Undergraduates	NA	SCL-90	Cross-sectional study
2005	Shi Xiaoning	Shanghai	Mean (SD): 21.39 (1.46)	Medicine	Undergraduates	Cluster sampling	CES-D	Cross-sectional study
2005	Gesang Zeren	Sichuan	Mean: 19.5	Public health and pharmacy	Undergraduates and college students	Cluster sampling	CES-D	Cross-sectional study
2005	Ren Huaneng	Hubei	Mean (SD): 20.07 (1.36)	Medicine	College students	Simple random sampling	SDS	Cross-sectional study
2005	Li Yingchun	Anhui	Mean (SD): 21.66 (1.15)	Medicine	Undergraduates	NA	SDS	Cross-sectional study
2005	Guo Rong	Guizhou	Mean (SD): 20.16 (1.43)	Medicine	Grade 2	Stratified and cluster sampling	SDS	Cross-sectional study
2005	Xu Limei	NA	Mean: 23	Medicine	Grade 5	Cluster sampling	SDS	Cross-sectional study
2005	Yang Xiuzhen	Shandong	Mean: 20.5	Medicine	Undergraduates	Stratified sampling	SDS	Cross-sectional study
2005	Wei Xiaoqing	Liaoning	Mean: 20	Medicine	Grades 1–2	Simple random sampling	SDS	Cross-sectional study
2005	Feng Fenglian	Hebei	NA	Medicine	Undergraduates	NA	SDS	Cross-sectional study
2006	Jin ji	Liaoning	Mean (SD): 20.79 (1.28)	Medicine	Undergraduates	Stratified and cluster sampling	SDS	Cross-sectional study
2006	Zhang Zewu	Guangdong	Mean (SD): 21.4 (2.6)	Medicine	Undergraduates	Cluster sampling	DIS	Cross-sectional study
2006	Zhai Dechun	NA	Mean (SD): 20.79 (1.28)	Medicine	Undergraduates	Stratified and cluster sampling	SDS	Cross-sectional study
2006	Wei Junbiao	Henan	Mean: 20	Medicine	Undergraduates	Cluster sampling	SDS	Cross-sectional study
2006	Zeng Qiang	NA	NA	Medicine	Undergraduates	Cluster sampling	SDS	Cross-sectional study
2006	Zhang Zewu	Guangdong	Mean (SD): 21.5 (2.3)	Medicine	Undergraduates	Cluster sampling	DSI	Cross-sectional study
2006	Mei Lin	Beijing	Mean: 21.5	Medicine	Undergraduates	Cluster sampling	SDS	Cross-sectional study
2006	Song Jing	Hubei	Mean: 22	Clinical medicine	Undergraduates	Cluster sampling	SCL-90	Cross-sectional study
2006	Wu Yan	Hubei	NA	Medicine	Undergraduates	NA	BDI	Cross-sectional study
2007	Meng Zhaoying	NA	Mean (SD): 20.71 (1.23)	Medicine	Undergraduates	Stratified and cluster sampling	SDS	Cross-sectional study
2007	Wang Tao	NA	Mean (SD): 20.82 (2.27)	Medicine	Undergraduates	Cluster sampling	SDS	Cross-sectional study
2007	Deng Shusong	Guangxi	Mean: 20	Medicine	Undergraduates	Cluster sampling	SCL-90	Cross-sectional study
2007	Sang Wenhua	Hebei	NA	Medicine	Grades 1–3	Cluster sampling	SDS	Cross-sectional study
2007	Liu Yulan	Jilin	Mean (SD): 22.6 (1)	Medicine	Undergraduates	Simple random sampling	SDS	Cross-sectional study
2007	Li Li	Liaoning	NA	Medicine	Undergraduates	Simple random sampling	SCL-90	Cross-sectional study
2008	Chen Zehua	Guangdong	NA	Medicine	College students and undergraduates	Cluster sampling	YRBSS	Cross-sectional study
2008	Li Yaqin	Hebei	Mean: 19.5	Medicine	College students	Simple random sampling and cluster sampling	DSI	Cross-sectional study
2009	Mu Yunzhen	Yunnan	Mean (SD): 21.86 (2.58)	Medicine	Undergraduates	Simple random sampling	SCL-90	Cross-sectional study
2009	Shang Yuxiu	Ningxia	Mean (SD): 20.62 (1.64)	Medicine	Undergraduates	Stratified and cluster sampling	SDS	Cross-sectional study
2009	Zhou Xin	Hebei, Jiangsu, and Ningxia	Mean (SD): 21.48 (1.242)	Nursing	Undergraduates	Cluster sampling	SDS	Cross-sectional study
2009	Li Wenwen	Guangdong	Mean: 25.5	Medicine	Undergraduates	Cluster sampling	CES-D	Cross-sectional study
2009	Yang Xiaohui	Sichuan	Mean: 21.5	Medicine	Undergraduates	NA	BDI	Cross-sectional study
2009	Jin Zhengguo	Jining	NA	Medicine	Undergraduates	NA	SCL-90	Cross-sectional study
2009	Zhao Shujuan	NA	NA	Medicine	Grade 1	Simple random sampling	SDS	Cross-sectional study
2010	Yanhui Liao	China	Mean (SD): 18.5 (0.8)	Medicine	Grade 1	Simple random sampling	SDS	Cross-sectional study
2011	Liang Sun	Anhui	Mean: 20	Medicine	Grades 1–2	NA	BDI	Cross-sectional study
2011	Dong Guanbo	Beijing	NA	Masters and doctors	8-year program student	Cluster sampling	SDS	Cross-sectional study
2011	Jiang Qing	Fujian	NA	Medicine	Undergraduates	Simple random sampling	HAD	Cross-sectional study
2011	Wei Yali	Guizhou	Mean: 20	Medicine	Grade 1	Stratified and cluster sampling	CES-D	Cross-sectional study
2011	Gao Shuhui	Hebei	Mean: 21	Medicine	Undergraduates	Stratified random sampling	SDS	Cross-sectional study
2011	Zhang Guifeng	Guangdong	Mean: 20.5	Medicine	Undergraduates	Stratified sampling	BDI	Cross-sectional study
2011	Zhao Qiuzhen	Hebei	NA	Medicine	Undergraduates	Cluster sampling	SDS	Cross-sectional study
2011	Xu Limei	NA	Mean: 19	Medicine	Undergraduates	NA	SDS	Cross-sectional study
2011	Tan Erli	NA	Mean (SD): 20.3 (1.1)	Medicine	College students	Cluster sampling	NA	Cross-sectional study
2012	Wang Na	Beijing	NA	Medicine	Undergraduates	Stratified and cluster sampling	IVR(self-made)	Cross-sectional study
2012	Li Wei	Chongqing	NA	Medicine	Undergraduates	Cluster sampling	SCL-90	Cross-sectional study
2012	Yang Chuanwei	Henan	Mean (SD): 20.67 (1.43)	Medicine	Undergraduates	Stratified and cluster sampling	SDS	Cross-sectional study
2012	Yang Yanfang	Inner Mongolia	Mean: 21.5	Medicine	Grade 1–3	NA	SDS	Cross-sectional study
2012	Shi Shenchao	Henan	Mean (SD): 20.67 (1.43)	Medicine	Undergraduates	Simple random sampling	SDS	Cross-sectional study
2012	Ding Jianfei	NA	NA	Medicine	Undergraduates	Cluster sampling	CES-D	Cross-sectional study
2012	Liu Xiuhua	Hebei	Mean: 21.5	Medicine	Undergraduates	Simple random sampling	SDS	Cross-sectional study
2013	Wang Dongping	Henan	Mean (SD): 19.98 (1.15)	Medicine	Undergraduates	Simple random sampling	SDS	Rct
2013	Wang Jun	Anhui	Mean (SD): 19.66 (0.96)	Medicine	Undergraduates	Cluster sampling	SDS	Cross-sectional study
2013	Liu Rui	Gansu	NA	Medicine	Undergraduates	Cluster sampling	SDS	Cross-sectional study
2013	Ren Xiaohui	NA	Mean (SD): 21 (1)	Medicine	Undergraduates	NA	SDS	Cross-sectional study
2014	Fan Yang	Hubei	Mean: 20.5	Medicine	Undergraduates	Stratified cluster sampling	SCL-90	Cross-sectional study
2014	Yao Ran	Guangdong	Mean: 21	Medicine	Undergraduates	Stratified and cluster sampling	SDS	Cross-sectional study
2014	Kunmi Sobowale	Mainland China	NA	Medicine	Grades 2 and 3	NA	PHQ-9	Cross-sectional study
2014	Qu Wei	Anhui	Mean (SD): 20.3 (2.09)	Medicine	Grades 1–2	Stratified and cluster sampling	SDS	Cross-sectional study
2014	Tao Shuman	Anhui	Mean (SD): 20 (1)	Medicine	Grades 1–3	Convenience sampling	SDS	Cross-sectional study
2014	Xian Pengcheng	Inner Mongolia	Mean: 21.5	Medicine	Undergraduates	Simple random sampling	SDS	Cross-sectional study
2014	Wang Feiran	Hubei, Shanxi, and Hebei	Mean (SD): 21.45 (1.37)	Medicine	Undergraduates	Stratified and cluster sampling	SCL-90	Cross-sectional study
2014	Liu Mei	Fujian	NA	Medicine	Undergraduates	Simple random sampling	SDS	Cross-sectional study
2014	Guo Kai	Qinghai	Mean (SD): 21.26 (1.20)	Medicine	Grades 2–4	Stratified and cluster sampling	SDS	Cross-sectional study
2015	Xiongfei Panan	23 provinces	Mean (SD): 20.7 (1.6)	Medicine	Undergraduates	NA	BDI	Cross-sectional study
2015	Liu Yan	Beijing	Mean: 21.5	Medicine	Undergraduate and postgraduate	Stratified sampling	CES-D	Cross-sectional study
2015	Chang Hong	Xinan	Mean (SD): 20.2 (1.5)	Medicine	Undergraduates	Simple random sampling	SDS	Cross-sectional study
2015	C.-J.CHEN	Taiwan	Mean (SD): 17.42 (1.03)	Nursing students	College students	NA	ADI	Cross-sectional study
2015	Meng Shi	Liaoning	Mean: 21.5	Medicine	Undergraduates and postgraduates	Cluster sampling	CES-D	Cross-sectional study
2015	Yu Jiegen	Anhui	NA	Medicine	Undergraduates	Simple random sampling	SDS	Cross-sectional study
2015	Zhao Chuan	Henan	Mean: 22.5	Medicine	Undergraduates	Stratified and cluster sampling	SDS	Cross-sectional study
2015	Yu Linlu	Beijing	Mean: 22	Medicine	Undergraduates	Cluster sampling	CES-D	Cross-sectional study
2015	Yu Linlu	Beijing	Mean: 22	Medicine	Undergraduates	Cluster sampling	CES-D	Cross-sectional study
2015	Han Yashu	Liaoning	NA	Medicine	Undergraduates	NA	SDS	Cross-sectional study
2016	Meng Shi	Liaoning	Mean (SD): 21.65 (1.95)	Medicine	Grades 1–7	Cluster sampling	CES-D	Cross-sectional study
2016	Gao Jie	Anhui	NA	Medicine	Undergraduates	Cluster sampling	SDS	Cross-sectional study
2016	Jiang Hongcheng	Yunnan	Mean (SD): 21.04 (1.84)	Medicine	Undergraduates	Stratified and cluster sampling	SDS	Cross-sectional study
2016	Huang Yalian	Sichuan	Mean: 21	Medicine	Grades 1–3	Simple random sampling	SDS	Cross-sectional study
2016	Qian Yunke	Jiangsu	NA	Medicine	Undergraduates	Stratified and cluster sampling	SDS	Cross-sectional study
2016	Lv Shixin	Shandong	NA	Medicine	Undergraduates	Stratified and cluster sampling	SDS	Cross-sectional study
2016	Qiu Nan	Sichuan	NA	Medicine	Undergraduates	Convenience sampling and cluster sampling	BDI	Cross-sectional study
2016	Wu Yingping	NA	NA	Medicine	Undergraduates	Cluster sampling and convenience sampling	BDI	Cross-sectional study
2017	Li Xue	NA	NA	Medicine	Undergraduates	Stratified and cluster sampling	CES-D	Cross-sectional study
2017	Chen Huan	Ningxia	NA	Medicine	Undergraduates	Stratified and cluster sampling	SDS	Cross-sectional study
2017	Xu Tao	Sichuan and Inner Mongolia	NA	Medicine	Undergraduates	Cluster sampling	BDI	Cross-sectional study
2017	Dai Ruoyi	Chongqing	NA	Medicine	Undergraduates	Stratified and cluster sampling	SDS	Cross-sectional study
2018	Ching-Yen Chen	Taiwan	Mean: 23.5	Medicine	Undergraduates	Simple random sampling	BDI	Multi-staged sampling
2018	Lin Fen	Hubei	NA	Medicine	Undergraduates	Stratified random sampling	BDI	Cross-sectional study
2018	Shi Junfang	Shanxi	Mean: 20.2	Medicine	Undergraduates	Stratified and cluster sampling	SDS/HAMD	Cross-sectional study
2018	Li Xiaoping	Jiangxi	NA	Medicine	Grades 2–4	Stratified and cluster sampling	SDS	Cross-sectional study
2018	Jiang Nan	Liaoning	NA	Medicine	Undergraduates	Simple random sampling	CES-D	Cross-sectional study
2018	Li Xuanxuan	Jilin	Mean (SD): 21.54 (1.98)	Medicine	Undergraduates	Cluster sampling	SDS	Cross-sectional study
2018	Sibo Zhao	China	Mean (SD): 20.25 (3.25)	Medicine	Undergraduates	NA	CES-D	Cross-sectional study
2018	Feng Fenglian	Hebei	NA	Medicine	Grades 1–3	Simple random sampling	SDS	Cross-sectional study
2018	Wu Jinting	Anhui	Mean (SD): 19.39 (0.85)	Medicine	Undergraduates	Stratified sampling	BDI	Cross-sectional study
2019	Jessica A Gold	Hunan	Mean (SD): 22 (1.5)	Medicine	Grades 3–6	Convenience sampling	PHQ-2	Cross-sectional study
2019	Chunli Liu	Northeast	Mean (SD): 31.1 (5.3)	Medicine	Doctoral students	Snowball sampling and stratified sampling	PHQ-9	Cross-sectional study
2019	Ling Wang	Anhui	Mean: 20.5	Medicine	College students and undergraduates	Simple random sampling	DASS-21	Cross-sectional study
2019	Xiaogang Zhong	China	NA	Medicine	Postgraduates and doctors	NA	PRIME-MD	Cross-sectional study
2019	Yanli Zeng	Sichuan	Mean (SD): 20.2 (1.2)	Nursing students	Grades 1–3	Stratified random cluster sampling	DASS-21	Cross-sectional study
2019	Zhao Xiuzhuan	Beijing	NA	Masters and doctors	8-year program student	Simple random sampling	SDS	Cross-sectional study
2019	Xiong Lin	Chongqing	NA	Medicine	College students	Stratified and cluster sampling	BDI	Cross-sectional study
2019	Tang Siyao	Guangdong	Mean (SD): 20.07 (1.49)	Medicine	Undergraduates	Convenience sampling	PHQ-9	Cross-sectional study
2019	Cao Lei	Chongqing	Mean (SD): 18.56 (0.99)	Medicine	Undergraduates	Stratified and cluster sampling	BDI	Cross-sectional study
2019	Steven W. H. Chau	HongKong	NA	Medicine	NA	Simple random sampling	NA	Cross-sectional study
2019	Lin Xin	Xinjiang	NA	Medicine	Grades 1–2	Stratified and cluster sampling	CES-D	Cross-sectional study
2019	Ai Dong	NA	NA	Medicine	Undergraduates	Stratified and cluster sampling	SDS	Cross-sectional study
2020	Yanmei Shen	Hunan	Mean (SD): 18.77 (1.09)	Medicine	College students and undergraduates	Convenience sampling	SDS	Cross-sectional study
2020	Jing Guo	Heilongjiang	Mean (SD): 19.48 (0.85)	Medicine	Grades 2–3	Cluster sampling	BDI-II	Cross-sectional study
2020	Ruyue Shao	Chongqing	Mean (SD): 19.76 (1.17)	Medicine	Grades 1–3	NA	SDS	Cross-sectional study
2020	Chen Jun	NA	Mean (SD): 19.63 (1.28)	Medicine	Grades 1–2	Stratified and cluster sampling	SDS	Cross-sectional study
2020	Yang Xueling	Guangdong	Mean (SD): 18.37 (0.73)	Medicine	Undergraduates	Convenience sampling	BDI-II	Cross-sectional study
2020	Li Ningning	Beijing	NA	Clinical medicine	Grades 5–7	Cluster sampling	Self-made questionnaire	Cross-sectional study
2020	Xiao Rong	Guangdong	Mean (SD): 19.92 (1.04)	Medicine	Undergraduates	Convenience sampling	PHQ-9	Cross-sectional study
2020	Zhu Huiquan	Hainan	Mean: 14.5	Medicine	Undergraduates	Stratified and cluster sampling	SCL-90	Cross-sectional study

NA, not available; SD, Standard Deviation; SDS, Self-Rating Depression Scale; BDI, Beck Depression Rating Scale; BDI-II, Beck Depression Inventory-II; BDI-13, Beck Depression Inventory-13; CES-D, Center for Epidemiologic Studies Depression Scale; SCL-90, the symptom checklist-90; HAMD, Hamilton Depression Scale; HAD, Hospital Anxiety and Depression Scale; IVR, interactive voice response; DSI, Depression Status Inventory; IDLS, the international depression literacy survey; ADI, Adolescent Depression Inventory; DASS-21, Depression Anxiety Stress Scale 21; PRIME-MD, The 2-Item Primary Care Evaluation of Mental Disorders; YRBSS, Youth Risk Behavior Surveillance System Questionnaire.

**Table 2:** Characteristics of the 80 studies included on anxiety in this review.

**Year**	**First author**	**Province**	**Age, years**	**Major**	**Grade**	**Sampling method**	**Measurement tools and cutoff score**	**Study type**
2000	Lin Daxi	Fujian	Mean: 19	Medicine	College students	Cluster sampling	SAS	Cross-sectional study
2000	Yang Benfu	NA	Mean: 20.5	Medicine	Undergraduates	Stratified and cluster sampling	SAS	Cross-sectional study
2001	Huang Juan	Guangdong	Mean (SD): 21.02 (1.87)	Medicine	Undergraduates	NA	SAS	Cross-sectional study
2001	Su Xiaomei	Guangdong	Mean (SD): 19.37 (1.3)	Nursing	Grades 1–4	Cluster sampling	SAS	Cross-sectional study
2001	Zhang Yushan	Anhui	Mean (SD): 21.8 (3.2)	Medicine	Undergraduates	NA	SAS	Cross-sectional study
2001	Zhang Yunsheng	Henan	NA	Pharmacy and nursing	Undergraduates	Simple random sampling	SCL-90	Cross-sectional study
2002	Qi Yulong	Anhui	NA	Medicine	Grade 1	Simple random sampling	SAS	Cross-sectional study
2002	Xu Limei	NA	Mean: 19	Medicine	Grade 1	stratified and cluster sampling	SDS	Cross-sectional study
2003	Zhang Xinwen	Hebei	NA	Medicine	Undergraduates	NA	MAS	Cross-sectional study
2003	Zheng Wenjun	Guangxi	Mean: 20	Clinical medicine	Undergraduates	Cluster sampling	S-AI	Cross-sectional study
2004	Zhang Fuquan	Hunan	Mean (SD): 19.85 (1.18)	Medicine	Undergraduates	Stratified and cluster sampling	SCL-90	Cross-sectional study
2004	Zhang Shuying	NA	Mean (SD): 21.8 (0.89)	Medicine	Undergraduates	NA	SCL-90	Cross-sectional study
2005	Ren Huaneng	Hubei	Mean (SD): 20.07 (1.36)	Medicine	College students	Simple random sampling	SAS	Cross-sectional study
2005	Li Yingchun	Anhui	Mean (SD): 21.66 (1.15)	Medicine	Undergraduates	NA	SAS	Cross-sectional study
2005	Xu Limei	NA	Mean: 23	Medicine	Grade 5	Cluster sampling	SAS	Cross-sectional study
2005	Yang Xiuzhen	Shandong	Mean: 20.5	Medicine	Undergraduates	Stratified sampling	SAS	Cross-sectional study
2005	Wei Xiaoqing	Liaoning	Mean: 20	Medicine	Grades 1–2	Simple random sampling	SAS	Cross-sectional study
2005	Feng Fenglian	Hebei	NA	Medicine	Undergraduates	NA	SAS	Cross-sectional study
2006	Jin ji	Liaoning	Mean (SD): 20.79 (1.28)	Medicine	Undergraduates	Stratified and cluster sampling	SAS	Cross-sectional study
2006	Zhai Dechun	NA	Mean (SD): 20.79 (1.28)	Medicine	Undergraduates	Stratified and cluster sampling	NA	Cross-sectional study
2006	Wei Junbiao	Henan	Mean: 20	Medicine	Undergraduates	Cluster sampling	SAS	Cross-sectional study
2006	Wang Yanfang	Guangdong	NA	Medicine	Undergraduates	Simple random sampling	SAS	Cross-sectional study
2006	Mei Lin	Beijing	Mean: 21.5	Medicine	Undergraduates	Cluster sampling	SAS	Cross-sectional study
2006	Song Jing	Hubei	Mean: 22	Clinical medicine	Undergraduates	Cluster sampling	SCL-90	Cross-sectional study
2007	Meng Zhaoying	NA	Mean (SD): 20.71 (1.23)	Medicine	Grades 1–3 college students	Stratified and cluster sampling	SAS	Cross-sectional study
2007	Liang xinrong	Guangxi	NA	Medicine	Undergraduates	Simple random sampling and cluster sampling	HAMA	Cross-sectional study
2007	Deng Shusong	Guangxi	Mean: 20	Medicine	Undergraduates	Cluster sampling	SCL-90	Cross-sectional study
2007	Liu Yulan	Jilin	Mean (SD): 22.6 (1)	Medicine	Undergraduates	Simple random sampling	SAS	Cross-sectional study
2007	Li Li	Liaoning	NA	Medicine	Undergraduates	Simple random sampling	SCL-90	Cross-sectional study
2009	Mu Yunzhen	Yunnan	Mean (SD): 21.86 (2.58)	Medicine	Undergraduates	Simple random sampling	SCL-90	Cross-sectional study
2009	Zhou Xin	Hebei, Jiangsu, and Ningxia	Mean (SD): 21.48 (1.242)	Nursing	Undergraduates	Cluster sampling	SAS	Cross-sectional study
2009	Liu Kerong	NA	Mean: 24	Medicine	Undergraduates	Stratified sampling	HAMA	Cross-sectional study
2010	Yanhui Liao	China	Mean (SD): 18.5 (0.8)	Medicine	Grades 1	Simple random sampling	SIAS	Cross-sectional study
2010	Feng Tianyi	Ningxia	NA	Medicine	Undergraduates	Stratified sampling	SAS	Cross-sectional study
2010	Wang Fengsheng	Anhui	Mean (SD): 19.33 (1.18)	Medicine	Grades 1–2	Cluster sampling	BAI	Cross-sectional study
2010	Ge Xin	Liaoning	Mean: 17	Medicine	College students	Simple random sampling	SCARED	Cross-sectional study
2011	Ruan Ye	Gansu	NA	Medicine	Undergraduates	Stratified and cluster sampling	SAS	Cross-sectional study
2011	Liang Sun	Anhui	Mean: 20	Medicine	Grades 1–2	NA	BAI	Cross-sectional study
2011	Zhu Shuang	Heilongjiang	Mean (SD): 21.32 (1.4)	Medicine	Undergraduates	Stratified sampling	SAS	Cross-sectional study
2011	Jiang Qing	Fujian	NA	Medicine	Undergraduates	Simple random sampling	HAD	Cross-sectional study
2011	Pan Xin	Shanxi	Mean (SD): 20.96 (1.36)	Medicine	Undergraduates	Stratified sampling	SAS	Cross-sectional study
2011	Zhao Qiuzhen	Hebei	NA	Medicine	Undergraduates	Cluster sampling	SAS	Cross-sectional study
2011	Xu Limei	NA	Mean: 19	Medicine	Undergraduates	NA	SAS	Cross-sectional study
2012	Li Wei	Chongqing	NA	Medicine	Undergraduates	Cluster sampling	SCL-90	Cross-sectional study
2012	Yang Chuanwei	Henan	Mean (SD): 20.67 (1.43)	Medicine	Undergraduates	Stratified and cluster sampling	SAS	Cross-sectional study
2013	Wang Dongping	Henan	Mean (SD): 19.98 (1.15)	Medicine	Undergraduates	Simple random sampling	SAS	Rct
2014	Fan Yang	Hubei	Mean: 20.5	Medicine	Undergraduates	Stratified cluster sampling	SCL-90	Cross-sectional study
2014	Qu Wei	Anhui	Mean (SD): 20.3 (2.09)	Medicine	Grades 1–2	Stratified and cluster sampling	HAMA	Cross-sectional study
2014	Chen Fuxun	Shandong	Mean (SD): 20.55 (1.34)	Medicine	Undergraduates	Cluster sampling	SAS	Cross-sectional study
2014	Wang Feiran	Hubei, Shanxi, and Hebei	Mean (SD): 21.45 (1.37)	Medicine	Undergraduates	Stratified and cluster sampling	SCL-90	Cross-sectional study
2015	Meng Shi	Liaoning	Mean: 21.5	Medicine	Undergraduates and postgraduates	Cluster sampling	SAS	Cross-sectional study
2015	Tian Yunqing	Beijing	Mean: 21.5	Medicine	Undergraduates	Cluster sampling	BAI	Cross-sectional study
2015	Chang Hong	Xinan	Mean (SD): 20.2 (1.5)	Medicine	Undergraduates	Simple random sampling	SAS	Cross-sectional study
2015	Li Qiang	Henan	NA	Medicine	Grades 2 and 3	Stratified and cluster sampling	SAS	Cross-sectional study
2015	Zhao Chuan	Henan	Mean: 22.5	Medicine	Undergraduates	Stratified and cluster sampling	SAS	Cross-sectional study
2016	Jiang Hongcheng	Yunnan	Mean (SD): 21.04 (1.84)	Medicine	Undergraduates	Stratified and cluster sampling	SAS	Cross-sectional study
2016	Sun Weiwei	NA	Mean (SD): 22.12 (2.53)	Medicine	Undergraduates	Simple random sampling	SAS	Cross-sectional study
2017	Feng Fenglian	Hebei	Mean: 20	Clinical medicine	Grades 1–3	Cluster sampling	SAS	Cross-sectional study
2017	Li Xiang	Liaoning	Mean: 21.42	Medicine	Undergraduates	Simple random sampling	SAS	Cross-sectional study
2017	Chen Huan	Ningxia	NA	Medicine	Undergraduates	Stratified and cluster sampling	SAS	Cross-sectional study
2017	Liang Peiyu	Qinghai	NA	Medicine	Undergraduates	Stratified random sampling	SAS	Cross-sectional study
2017	Xu Tao	Sichuan and Inner Mongolia	NA	Medicine	Undergraduates	Cluster sampling	SAS	Cross-sectional study
2018	Ching-Yen Chen	Taiwan	Mean: 23.5	Medicine	Undergraduates	Simple random sampling	BAI	Multi-staged sampling
2018	Zhao Fei	China	Mean (SD): 20.7 (1.6)	Medicine	Undergraduates	Simple random sampling	SAS	Cross-sectional study
2018	Li Xuanxuan	Jilin	Mean (SD): 21.54 (1.98)	Medicine	Undergraduates	Cluster sampling	SAS	Cross-sectional study
2018	Feng Fenglian	Hebei	NA	Medicine	Grades 1–3	Simple random sampling	SAS	Cross-sectional study
2019	Chunli Liu	Northeast	Mean (SD): 31.1 (5.3)	Medicine	Doctoral students	Snowball sampling and stratified sampling	GAD-7	Cross-sectional study
2019	Ling Wang	Anhui	Mean: 20.5	Medicine	College students and undergraduates	Simple random sampling	DASS-21	Cross-sectional study
2019	Yanli Zeng	Sichuan	Mean (SD): 20.2 (1.2)	Nursing students	Grades 1–3	Stratified random cluster sampling	DASS-21	Cross-sectional study
2019	Zhao Xiuzhuan	Beijing	NA	Masters and doctors	8-year program student	Simple random sampling	SAS	Cross-sectional study
2019	Wang Zhe	Heilongjiang	NA	Medicine	Undergraduates	Cluster sampling	SAS	Cross-sectional study
2019	Steven W. H. Chau	Hong Kong	NA	Medicine	NA	Simple random sampling	GHQ-12	Cross-sectional study
2019	Li Zhongcheng	Guangdong	NA	Medicine	Undergraduates	Stratified and cluster sampling	SAS	Cross-sectional study
2019	Ai Dong	NA	NA	Medicine	Undergraduates	Stratified and cluster sampling	SAS	Cross-sectional study
2020	Yanmei Shen	Hunan	Mean (SD): 18.77 (1.09)	Medicine	College students and undergraduates	Convenience sampling	SAS	Cross-sectional study
2020	Ruyue Shao	Chongqing	Mean (SD): 19.76 (1.17)	Medicine	Grades 1–3	NA	SAS	Cross-sectional study
2020	Chen Jun	NA	Mean (SD): 19.63 (1.28)	Medicine	Grades 1–2	Stratified and cluster sampling	SAS	Cross-sectional study
2020	Yang Xueling	Guangdong	Mean (SD): 18.37 (0.73)	Medicine	Undergraduates	Convenience sampling	BAI	Cross-sectional study
2020	Li Ningning	Beijing	NA	Clinical medicine	Grades 5–7	Cluster sampling	Self-made questionnaire	Cross-sectional study
2020	Liu Xia	NA	Mean (SD): 20.38 (2.07)	Medicine	Undergraduates	Stratified and cluster sampling	SAS	Cross-sectional study

NA, not available; SD, Standard Deviation; BAI, Beck Anxiety Inventory; DASS-21, Depression Anxiety Stress Scale 21; GAD-7, Generalized Anxiety Disorder-7; GHQ-12, 12-Item General Health Questionnaire; HAD, Hospital Anxiety and Depression Scale; HAMA, Hamilton Depression Scale; MAS, Manifest Anxiety Scale; S-AI, State-Anxiety Inventory; SAS, Self-Rating Anxiety Scale; SCARED, Rating Scale Scoring Aide; SCL-90, the symptom checklist-90; STAI-6, the 6-item state version of the State-Trait Anxiety Inventory.

**Table 3:** Characteristics of the 21, 53, and 14 studies included on suicidal attempt, suicidal ideation, and suicidal plan in this review.

**Year**	**First author**	**Province**	**Age, years**	**Major**	**Grade**	**Sampling method**	**Measurement tools and cutoff score**	**Study type**
**Suicide attempt**								
2002	Hu Liren	NA	Mean: 21	Medicine	Undergraduates	NA	Self-made questionnaire	Cross-sectional study
2005	Hu Liren	NA	Mean (SD): 21.22 (1.35)	Medicine	Undergraduates	Stratified and cluster sampling	Self-made questionnaire	Cross-sectional study
2005	Wang Dequan	NA	NA	Medicine	Undergraduates	Stratified sampling	Self-made questionnaire	Cross-sectional study
2007	Hu Liren	NA	Mean (SD): 20.57 (1.44)	Medicine	Undergraduates	Stratified and cluster sampling	Self-made questionnaire	Cross-sectional study
2008	Ou Guangzhong	Fujian	Mean: 20	Medicine	Grades 1 and 3	Cluster sampling	QSA and Suicide ideation question	Cross-sectional study
2008	Chen Zehua	Guangdong	NA	Medicine	College students and undergraduates	Cluster sampling	Based on YRBSS	Cross-sectional study
2008	Hu Zhihong	Shanghai	Mean (SD): 21.36 (1.62)	Clinical	Undergraduates	Stratified and cluster sampling	Self-made questionnaire	Cross-sectional study
2008	Fan Yinguang	Anhui	Mean (SD): 20.15 (1.67)	Medicine	Undergraduates	Stratified and cluster sampling		Cross-sectional study
2009	Shang Yuxiu	Ningxia	Mean (SD): 20.62 (1.64)	Medicine	Undergraduates	Stratified and cluster sampling	Self-made questionnaire	Cross-sectional study
2009	Cao Hongyuan	Anhui	Mean (SD): 19.33 (1.17)	Medicine	Grades 1–2	Simple random sampling	Self-made questionnaire	Cross-sectional study
2009	Zeng Zhuanping	NA	NA	Medicine	Grades 1–3	Stratified and cluster sampling	Self-made questionnaire	Cross-sectional study
2010	Xin Shen	Anhui	Mean (SD): 20.56 (1.58)	Medicine	Undergraduates	Cluster sampling	SIOSS	Cross-sectional study
2012	Wan Yuhui	Anhui	SD: 20.5 ± 1.1	Medicine	Grades 1–2	Cluster sampling	Self-made questionnaire	Cross-sectional study
2013	Zhang Yuan	Yunnan	NA	Medicine	Undergraduates	Stratified and simple random sampling	Self-made questionnaire	Cross-sectional study
2014	Yang Linsheng	Anhui	Mean (SD): 19.6 (1.3)	Medicine	Grades 1–2	Simple random sampling	Self-made questionnaire	Cross-sectional study
2014	Yang Linsheng	Anhui	Mean (SD): 19.6 (1.3)	Medicine	Grades 1–2	Cluster sampling	Self-made questionnaire	Cross-sectional study
2017	Long Sun	NA	Mean (SD): 20.25 (1.23)	Medicine	Undergraduates	Simple random sampling	Self-made questionnaire	Cross-sectional study
2018	Zeng Baoer	Guangdong	Mean (SD): 25.79 (4.47)	Medicine	Undergraduates	NA	SBQ-R	Cross-sectional study
2020	Wanjie Tang	NA	NA	Medicine	Undergraduates	Simple random sampling	NCS	Cross-sectional study
2020	Yanmei Shen	Hunan	Mean (SD): 18.77 (1.09)	Medicine	College students and undergraduates	Convenience sampling	Self-made questionnaire	Cross-sectional study
2020	Chen Jun	NA	Mean (SD): 19.63 (1.28)	Medicine	Grades 1–2	Stratified and cluster sampling	Self-made questionnaire	Cross-sectional study
**Suicide ideation**								
2002	Hu Liren	NA	Mean: 21	Medicine	Undergraduates	NA	Self-made questionnaire	Cross-sectional study
2004	Liang Duohong	Liaoning	Mean (SD): 20.8 (0.8)	Medicine	Grades 1–3 and college students	Stratified and cluster sampling	Self-made questionnaire	Cross-sectional study
2005	Hu Liren	NA	Mean (SD): 21.22 (1.35)	Medicine	Undergraduates	Stratified and cluster sampling	Self-made questionnaire	Cross-sectional study
2005	Wang Dequan	NA	NA	Medicine	Undergraduates	Stratified sampling	Self-made questionnaire	Cross-sectional study
2006	Wang Xuelian	Fujian	NA	Medicine	Grades 1–3 and 5	Simple random sampling	Self-made questionnaire	Cross-sectional study
2007	Hu Liren	NA	Mean (SD): 20.57 (1.44)	Medicine	Undergraduates	Stratified and cluster sampling	Self-made questionnaire	Cross-sectional study
2007	Zhang Xiaoyuan	Guangdong	Mean (SD): 20.3 (2.7)	Medicine	Undergraduates	Cluster sampling	EPQ	Cross-sectional study
2008	Ou Guangzhong	Fujian	Mean: 20	Medicine	Grades 1 and 3	Cluster sampling	QSA and Suicide ideation question	Cross-sectional study
2008	Wang Xing	Jiangxi	Mean: 22	Medicine	Undergraduates	Simple random sampling	EPQ	Cross-sectional study
2008	Hu Zhihong	Shanghai	Mean (SD): 21.36 (1.62)	Clinical medicine	Undergraduates	Stratified and cluster sampling	Self-made questionnaire	Cross-sectional study
2008	Yang Benfu	NA	NA	Medicine	Undergraduates	Cluster sampling	SIOSS	Cross-sectional study
2008	Qian Wencai	Huabei	NA	Medicine	Grades 1–3	Cluster sampling	AHRBI	Cross-sectional study
2008	Li Youzi	Liaoning	NA	Medicine	Undergraduates	Simple random sampling	SCL-90	Cross-sectional study
2008	Liu Baohua	Beijing	NA	Medicine	Grade 1	NA	Medical Student Risk Behavior Questionnaire	Cross-sectional study
2008	Chen Zehua	Guangdong	NA	Medicine	College students and undergraduates	Cluster sampling	YRBSS	Cross-sectional study
2008	Fan Yinguang	Anhui	Mean (SD): 20.15 (1.67)	Medicine	Undergraduates	Stratified and cluster sampling	BSSI	Cross-sectional study
2009	Shang Yuxiu	Ningxia	Mean (SD): 20.62 (1.64)	Medicine	Undergraduates	Stratified and cluster sampling	Self-made questionnaire	Cross-sectional study
2009	Cao Hongyuan	Anhui	Mean (SD): 19.33 (1.17)	Medicine	Grades 1–2	Simple random sampling	Self-made questionnaire	Cross-sectional study
2009	Yang Xiaohui	Sichuan	Mean: 21.5	Medicine	Undergraduates	NA	SIOSS	Cross-sectional study
2009	Zeng Zhuanping	NA	NA	Medicine	Grades 1–3	Stratified and cluster sampling	Self-made questionnaire	Cross-sectional study
2010	Song Yumei	Anhui	Mean (SD): 21.8 (1.64)	Medicine	Undergraduates	Stratified and cluster sampling	BSI-CV	Cross-sectional study
2010	Xin Shen	Anhui	Mean (SD): 20.56 (1.58)	Medicine	Undergraduates	Cluster sampling	SIOSS	Cross-sectional study
2010	Shen Liqin	NA	NA	Medicine	Undergraduates	Simple random sampling	Self-made questionnaire	Cross-sectional study
2010	Wang Jian	NA	Mean (SD): 22 (1.23)	Medicine	Grade 3	NA	SIBQ	Cross-sectional study
2010	Yang Yanjie	Heilongjiang	SD: 21.32 ± 2.195	Medicine	NA	Stratified random cluster sampling	Self-made questionnaire	Cross-sectional study
2012	Wan Yuhui	Anhui	SD: 20.5 ± 1.1	Medicine	Grades 1–2	Cluster sampling	Self-made questionnaire	Cross-sectional study
2012	Yang Chuanwei	Henan	Mean (SD): 20.67 (1.43)	Medicine	Undergraduates	Stratified and cluster sampling	SIOSS	Cross-sectional study
2012	Fan, A.P.	Taiwan	NA	Medicine	Undergraduates	Simple random sampling	Self-made questionnaire	Cross-sectional study
2013	Wu Ling	Hainan	Mean (SD): 21.51 (1.67)	Medicine and others	Undergraduates	Multi-stages sampling	SIOSS	Cross-sectional study
2013	Liu Chang	NA	Mean (SD): 19.63 (0.85)	Medicine	Undergraduates	Simple random sampling	UPI	Cross-sectional study
2013	Zhang Yuan	Yunnan	NA	Medicine	Undergraduates	Stratified and simple random sampling	Self-made questionnaire	Cross-sectional study
2014	Yang Linsheng	Anhui	Mean (SD): 19.6 (1.3)	Medicine	Grades 1–2	Simple random sampling	Self-made questionnaire	Cross-sectional study
2014	Yao Ran	Guangdong	Mean: 21	Medicine	Undergraduates	Stratified and cluster sampling	Self-made questionnaire	Cross-sectional study
2014	Kunmi Sobowale	Mainland China	NA	Medicine	Grades 2 and 3	NA	PHQ-9	Cross-sectional study
2014	Aiming Zheng	China	SD: 20.8 ± 1.36	Medicine	Grades 3–5	NA	BHS	Cross-sectional study
2014	Yang Linsheng	Anhui	Mean (SD): 19.6 (1.3)	Medicine	Grades 1–2	Cluster sampling	Self-made questionnaire	Cross-sectional study
2014	Liu Yan	Liaoning	Mean (SD): 20.79 (1.19)	Medicine	Grades 1–3	Stratified and cluster sampling	Self-made questionnaire	Cross-sectional study
2015	Zhang Kaili	Hunan	Mean: 20.5	Clinical and nursing	Undergraduates	Stratified sampling	PIL	Cross-sectional study
2015	Guan Suzhen	Xinjiang	Mean: 21	Medicine	Undergraduates	Stratified and cluster sampling	SSI	Cross-sectional study
2016	Dai Chengshu	NA	NA	Medicine	Undergraduates	Cluster sampling	BSSI	Cross-sectional study
2016	Lv Shixin	Shandong	NA	Medicine	Undergraduates	Stratified and cluster sampling	SIOSS	Cross-sectional study
2017	Long Sun	NA	Mean (SD): 20.25 (1.23)	Medicine	Undergraduates	Simple random sampling	Self-made questionnaire	Cross-sectional study
2017	Ma Xuan	Anhui	Mean (SD): 19.5 (1)	Medicine	Grades 1–2	Stratified and cluster sampling	Self-made questionnaire	Cross-sectional study
2018	Zeng Baoer	Guangdong	Mean (SD): 25.79 (4.47)	Medicine	Undergraduates	NA	SBQ-R	Cross-sectional study
2018	Zeng Baoer	Guangdong	Mean (SD): 25.79 (4.47)	Medicine	Undergraduates	NA	SBQ-R	Cross-sectional study
2018	Dan Wu	China	NA	Medicine	Undergraduates	Multi-staged sampling	Single item	Cross-sectional study
2018	Sibo Zhao	China	Mean (SD): 20.25 (3.25)	Medicine	Undergraduates	NA	SSI	Cross-sectional study
2018	Zheng Chuanjuan	Zhejiang	NA	Medicine	Undergraduates and postgraduates	Stratified sampling	Self-made questionnaire	Cross-sectional study
2019	Liu Jing	Anhui	Mean (SD): 20 (1.5)	Medicine	Undergraduates	Cluster sampling	Self-made questionnaire	Cross-sectional study
2020	Wanjie Tang	NA	NA	Medicine	Undergraduates	Simple random sampling	NCS	Cross-sectional study
2020	Yanmei Shen	Hunan	Mean (SD): 18.77 (1.09)	Medicine	College students and undergraduates	Convenience sampling	Self-made questionnaire	Cross-sectional study
2020	Chen Jun	NA	Mean (SD): 19.63 (1.28)	Medicine	Grades 1–2	Stratified and cluster sampling	Self-made questionnaire	Cross-sectional study
**Suicide plan**								
2002	Hu Liren	NA	Mean: 21	Medicine	Undergraduates	NA	Self-made questionnaire	Cross-sectional study
2004	Liang Duohong	Liaoning	Mean (SD): 2 (0.8)	Medicine	Grades 1–3 and college students	Stratified and cluster sampling	Self-made questionnaire	Cross-sectional study
2005	Wang Dequan	NA	NA	Medicine	Undergraduates	Stratified sampling	Self-made questionnaire	Cross-sectional study
2006	Wang Xuelian	Fujian	NA	Medicine	Grades 1–3 and 5	Simple random sampling	Self-made questionnaire	Cross-sectional study
2007	Hu Liren	NA	Mean (SD): 20.57 (1.44)	Medicine	Undergraduates	Stratified and cluster sampling	Self-made questionnaire	Cross-sectional study
2008	Ou Guangzhong	Fujian	Mean: 20	Medicine	Grades 1 and 3	Cluster sampling	QSA and Suicide ideation question	Cross-sectional study
2008	Hu Zhihong	Shanghai	Mean (SD): 21.36 (1.62)	Clinical	Undergraduates	Stratified and cluster sampling	Self-made questionnaire	Cross-sectional study
2009	Shang Yuxiu	Ningxia	Mean (SD): 20.62 (1.64)	Medicine	Undergraduates	Stratified and cluster sampling	Self-made questionnaire	Cross-sectional study
2012	Wan Yuhui	Anhui	SD: 20.5 ± 1.1	Medicine	Grades 1–2	Cluster sampling	Self-made questionnaire	Cross-sectional study
2017	Long Sun	NA	Mean (SD): 20.25 (1.23)	Medicine	Undergraduates	Simple random sampling	Self-made questionnaire	Cross-sectional study
2018	Zeng Baoer	Guangdong	Mean (SD): 25.79 (4.47)	Medicine	Undergraduates	NA	SBQ-R	Cross-sectional study
2020	Wanjie Tang	NA	NA	Medicine	Undergraduates	Simple random sampling	NCS	Cross-sectional study
2020	Yanmei Shen	Hunan	Mean (SD): 18.77 (1.09)	Medicine	College students and undergraduates	Convenience sampling	Self-made questionnaire	Cross-sectional study

NA, not available; SD, Standard Deviation; NCS, National Comorbidity Survey; QSA, Suicide Attitude Questionnaire; SBQ-R, The Suicide Behaviors Questionnaire-Revised; SIOSS, Self-rating Idea of Suicide Scale; PHQ-9, the Patient Health Questionnaire-9; BHS, Beck Hopelessness Scale; BSI-CV, Beck Scale for Suicide Ideation-Chinese Version; BSSI, Beck Scale for Suicidal Ideation; PIL, Purpose in Life Test; EPQ, Eysenck Personality Questionnaire; SIBQ, Suicidal Ideation and Behavior Questionnaire; SSI, Scale for Suicide Ideation; AHRBI, the Adolescent Health-Related Risky Behavior Inventory; SCL-90, the symptom checklist-90; UPI, University Personality Inventory; YRBSS, Youth Risk Behavior Surveillance System Questionnaire.

### Depression

**Figure 2:**
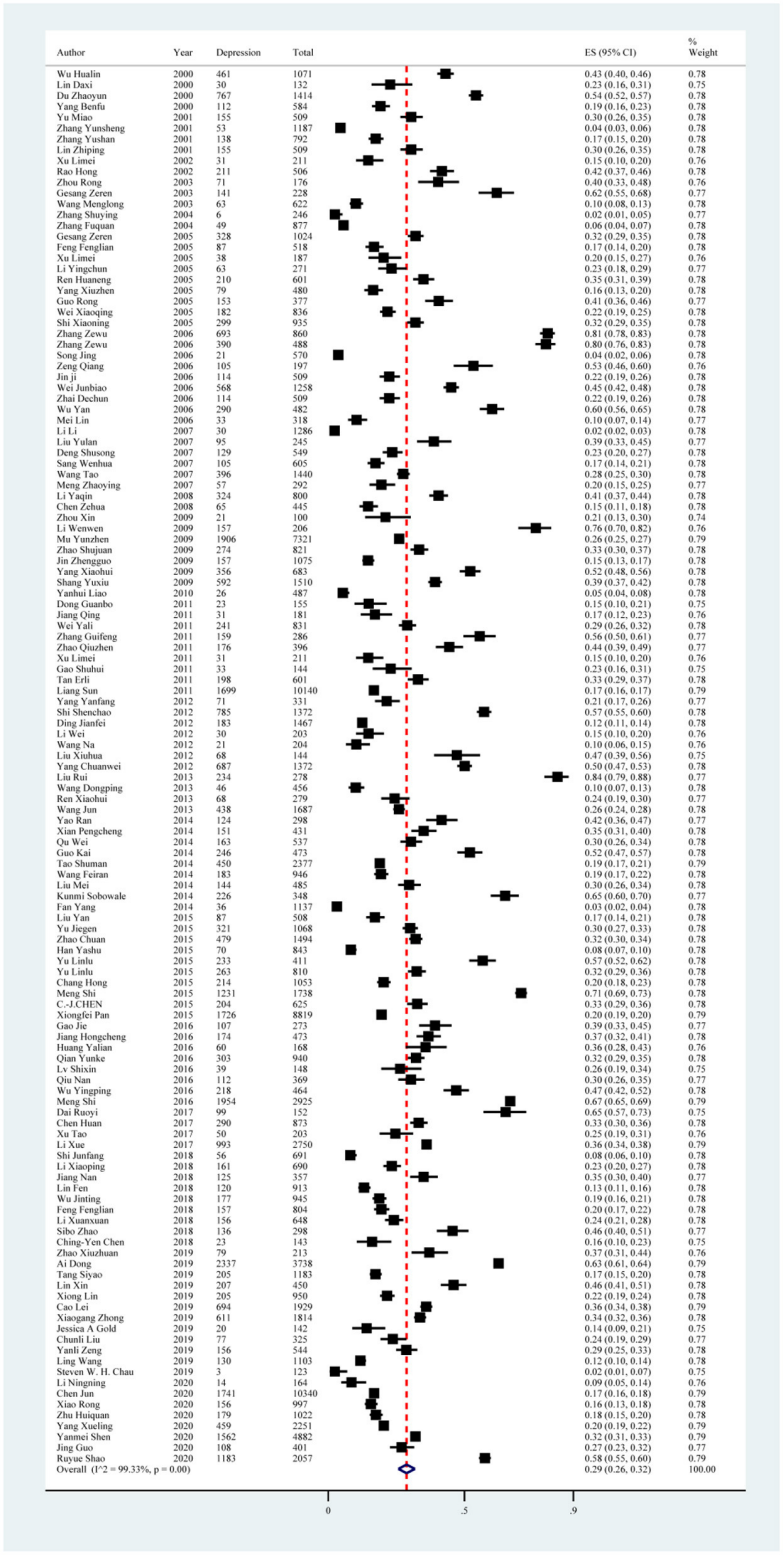
Forest plot of prevalence of depression in Chinese medical students.

Depression symptoms reported in the 129 included studies yielded a pooled prevalence of 29% (38,309/132,343; 95% CI: 26%−32%), with substantial evidence of between-study heterogeneity (*I*^2^ = 99.33%; Figure 2, Table 4). Sensitivity analysis showed that no individual study significantly affected the overall result (Supplementary material S5, Figure 1). In subgroup analysis, heterogeneity was reduced in studies using BDI with a score ≥ 14 (*I*^2^ = 87.97%), SCL-90 with a score ≥ 2 (*I*^2^ = 81.69%), and SCL-90 with a score ≥ 3 (*I*^2^ = 47.42%; Table 4).

**Table 4:** Estimated depression prevalence among medical students in China.

**Subgroup**	**No. of studies**	**No. of depression**	**Sample size**	**Subgroup analysis**				**Meta-regression**	
				**Estimated rate (95% CI)**	***Q***	*I*^2^ **(%)**	***p*** **-value**	*I*^2^ **(%)**	***p*** **-value**
**Study region**									
Northeast	12	4,299	11,188	0.28 (0.13, 0.45)	4,159.55	99.74%	<0.01	99.50	0.1682
North China	19	2,442	8,718	0.25 (0.19, 0.32)	914.95	98.03%	<0.01		
East China	23	6,076	26,384	0.26 (0.21, 0.30)	1,255.88	98.25%	<0.01		
South China	14	2,853	9,506	0.33 (0.21, 0.48)	2,553.71	99.49%	<0.01		
Central China	14	4,923	16,743	0.23 (0.13, 0.34)	3,313.32	99.61%	<0.01		
Northwest	5	1,569	3,584	0.51 (0.37, 0.66)	280.58	98.57%	<0.01		
Southwest	15	5,911	18,134	0.35 (0.28, 0.41)	1,064.86	98.69%	<0.01		
Multiple regions	8	2,979	13,015	0.28 (0.19, 0.38)	642.10	98.91%	<0.01		
*N*	19	7,254	25,071	0.28 (0.20, 0.37)	3,484.46	99.48%	<0.01		
**Survey year**									
2000–2005	24	3,882	14,293	0.25 (0.18, 0.32)	2,098.33	98.90%	<0.01	99.51	0.6012
2005–2010	25	7,018	23,056	0.31 (0.23, 0.40)	4,270.98	99.44%	<0.01		
2010–2015	39	11,773	45,139	0.30 (0.25, 0.36)	5,682.24	99.33%	<0.01		
2015–2020	41	15,636	49,855	0.28 (0.23, 0.34)	6,736.96	99.41%	<0.01		
**Sample size**									
<200	16	678	2,456	0.25 (0.17, 0.34)	363.84	95.88%	<0.01	99.54	0.6346
201–400	26	2,562	7,266	0.33 (0.25, 0.42)	1,429.42	98.25%	<0.01		
401–600	26	3,881	12,778	0.30 (0.23, 0.37)	2,066.31	98.79%	<0.01		
601–800	11	1,971	7,358	0.26 (0.18, 0.34)	669.56	98.51%	<0.01		
801–1,000	16	3,662	14,181	0.25 (0.17, 0.34)	1,996.52	99.25%	<0.01		
>1,000	34	25,555	88,304	0.29 (0.24, 0.35)	12,430.00	99.73%	<0.01		
**Sampling methods**									
Simple	25	5,645	22,132	0.24 (0.18, 0.31)	2,603.18	99.08%	<0.01	99.48	0.2927
Convenience	6	2,852	11,832	0.20 (0.14, 0.26)	293.03	98.29%	<0.01		
Stratified	4	502	2,219	0.26 (0.13, 0.41)	165.74	98.19%	<0.01		
Cluster	34	9,086	22,692	0.34 (0.26, 0.42)	5,467.54	99.40%	<0.01		
Multiple sampling methods	39	12,687	42,280	0.29 (0.24, 0.34)	5,625.12	99.32%	<0.01		
*N*	21	7,537	31,188	0.29 (0.22, 0.36)	3,046.89	99.34%	<0.01		
**Educational level**									
Undergraduate	122	36,181	1,27,448	0.29 (0.26, 0.32)	17,679.64	99.32%	<0.01	99.51	0.7368
Postgraduate	6	2,041	4,387	0.32 (0.14, 0.52)	793.43	99.37%	<0.01		
Unclassified	1	87	508	0.17 (0.14, 0.21)	–	–	–		
**Measurement tool and cutoff score**									
ADI score ≥ 8	1	204	625	0.33 (0.29, 0.36)	–	–	–	98.76	<0.001
BDI score ≥ 5	7	2,040	4,719	0.46 (0.38, 0.54)	166.95	96.41%	<0.01		
BDI score ≥ 10	1	1,699	10,140	0.17 (0.16, 0.17)	–	–	–		
BDI score ≥ 14	5	2,124	11,028	0.19 (0.15, 0.22)	33.24	87.97%	<0.01		
BDI without cutoff score reported	1	177	945	0.19 (0.16, 0.21)	–	–	–		
BDI-13 score ≥ 5	1	767	1,414	0.54 (0.52, 0.57)	–	–	–		
BDI-II score ≥ 14	2	567	2,652	0.21 (0.20, 0.23)	–	–	–		
CES-D score ≥ 16	10	4,951	9,557	0.46 (0.34, 0.58)	1,231.06	99.27%	<0.01		
CES-D score ≥ 20	7	1,937	6,399	0.34 (0.22, 0.48)	612.45	99.02%	<0.01		
DASS-21 score ≥ 10	2	286	1,647	0.17 (0.15, 0.19)	–	–	–		
DSI severity index ≥ 0.5	3	1,407	2,148	0.68 (0.40, 0.90)	–	–	–		
GHQ-12 score ≥ 2	1	3	123	0.02 (0.01, 0.07)	–	–	–		
HAD score ≥ 9	1	31	181	0.17 (0.12, 0.23)	–	–	–		
IVR(self-made) score ≥ 10	1	21	204	0.10 (0.06, 0.15)	–	–	–		
PHQ-2 score ≥ 3	1	20	142	0.14 (0.09, 0.21)	–	–	–		
PHQ-9 score ≥ 5	1	226	348	0.65 (0.60, 0.70)	–	–	–		
PHQ-9 score ≥ 10	3	438	2,505	0.18 (0.15, 0.22)	–	–	–		
PRIME-MD answer “yes”	1	611	1,814	0.34 (0.32, 0.36)	–	–	–		
SCL-90 score ≥ 1.8	1	1,906	7,321	0.26 (0.25, 0.27)	–	–	–		
SCL-90 score ≥ 2	5	678	3,795	0.18 (0.15, 0.21)	21.85	81.69%	<0.01		
SCL-90 score > 2	1	36	1,137	0.03 (0.02, 0.04)	–	–	–		
SCL-90 score ≥ 3	4	129	2,880	0.04 (0.03, 0.05)	5.71	47.42%	0.13		
SCL-90 without cutoff score reported	1	30	1,286	0.02 (0.02, 0.03)	–	–	–		
SDS score ≥ 5	1	163	537	0.30 (0.26, 0.34)	–	–	–		
SDS score ≥ 14	1	214	1,053	0.20 (0.18, 0.23)	–	–	–		
SDS score ≥ 40	2	150	656	0.22 (0.19, 0.25)	–	–	–		
SDS score ≥ 41	3	401	1,706	0.25 (0.11, 0.42)	–	–	–		
SDS score ≥ 42	1	144	485	0.30 (0.26, 0.34)	–	–	–		
SDS score ≥ 50	24	5,060	14,975	0.29 (0.23, 0.35)	1,413.90	98.37%	<0.01		
SDS score > 50	1	63	622	0.10 (0.08, 0.13)	–	–	–		
SDS score ≥ 52	1	303	940	0.32 (0.29, 0.35)	–	–	–		
SDS score ≥ 53	14	4,655	15,256	0.32 (0.25, 0.39)	976.16	98.67%	<0.01		
SDS severity index ≥ 0.5	12	4,548	9,083	0.38 (0.29, 0.48)	879.01	98.75%	<0.01		
SDS score ≥ 50 and HAMD	1	56	691	0.08 (0.06, 0.10)	–	–	–		
SDS without cutoff score reported	5	2,185	12,720	0.19 (0.13, 0.26)	147.09	97.28%	<0.01		
Self-made questions answers “yes”	1	14	164	0.09 (0.05, 0.14)	–	–	–		
YRBSS without cutoff score reported	1	65	445	0.15 (0.11, 0.18)					
Overall	129	38,309	1,32,343	0.29 (0.26, 0.32)	19,186.54	99.33%	<0.01		

N, not reported; HAD, Hospital Anxiety and Depression Scale; BDI, Beck Depression Rating Scale; BDI-II, Beck Depression Inventory-II; BDI-13, Beck Depression Inventory-13; CES-D, Center for Epidemiologic Studies Depression Scale; DASS-21, Depression Anxiety Stress Scale-21; DSI, Depression Status Inventory; GHQ, General Health Questionnaire; IDLS, the international depression literacy survey; IVR, interactive voice response; PHQ-2, The Patient Health Questionnaire-2; PHQ-9, The Patient Health Questionnaire-9; SCL-90, the symptom checklist-90; PRIME-MD, The 2-Item Primary Care Evaluation of Mental Disorders; SDS, Self-Rating Depression Scale; HAMD, Hamilton Depression Scale; YRBSS, Youth Risk Behavior Surveillance System Questionnaire.

**Figure 3:**
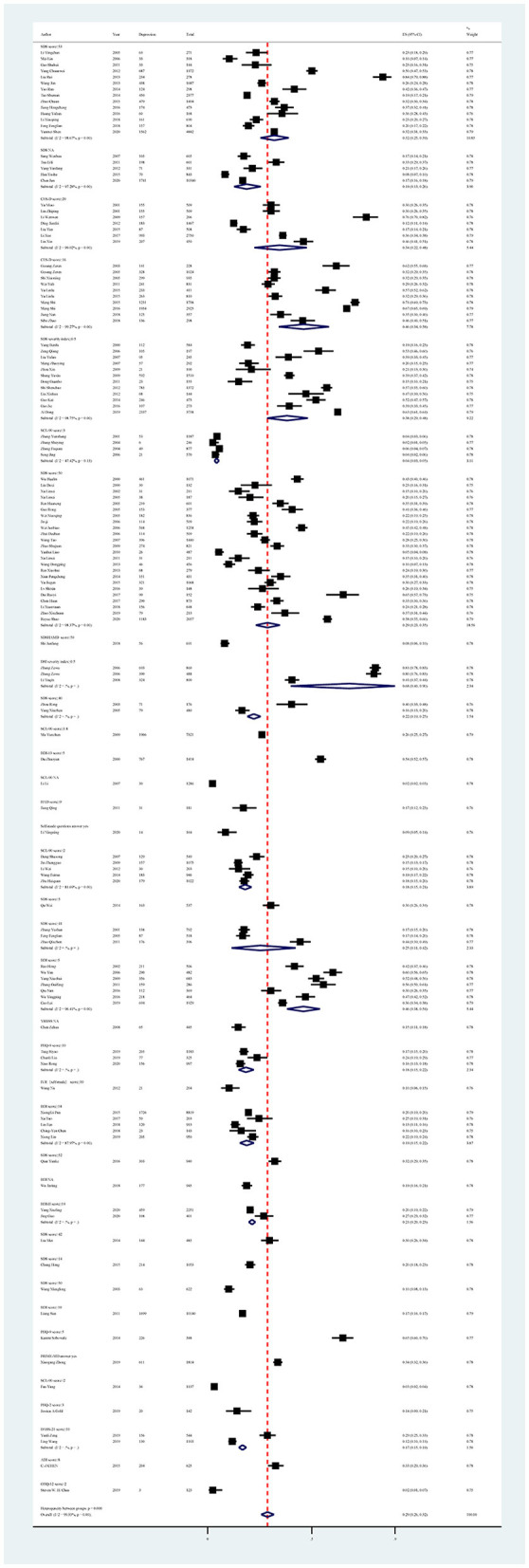
Subgroup analysis of depression in Chinese medical students based on measurements tools.

Subgroup analysis showed differences in prevalence based on study regions, recall periods, sampling methods, measurement tools, and cutoff scores. In this study, the pooled prevalence of depression symptoms was higher in the northwest region of China, with an estimate of 51% (95% CI: 37%−66%). Furthermore, studies conducted between 2005 and 2010 found a higher prevalence of depression symptoms (31%; 95% CI: 23%−40%). All studies that used a cluster sampling method reported a higher prevalence of depression symptoms than other sampling methods. In terms of measurement tool and cutoff score, studies using the Depression Status Inventory (DSI) with a severity index ≥ 0.5 and the BDI-13 with a score ≥ 5 reported a higher estimated prevalence, with a pooled prevalence of 68% (95% CI: 40%−90%) and 54% (95% CI: 52%−57%), respectively (Figure 3, Table 4).

In all univariate meta-regression analyses, only the measurement tool and cutoff score could explain the heterogeneity between studies (*p* < 0.001). The result of Egger's test showed publication bias, with *p* < 0.01 (Supplementary material S6, Figure 1).

### Anxiety

**Figure 4:**
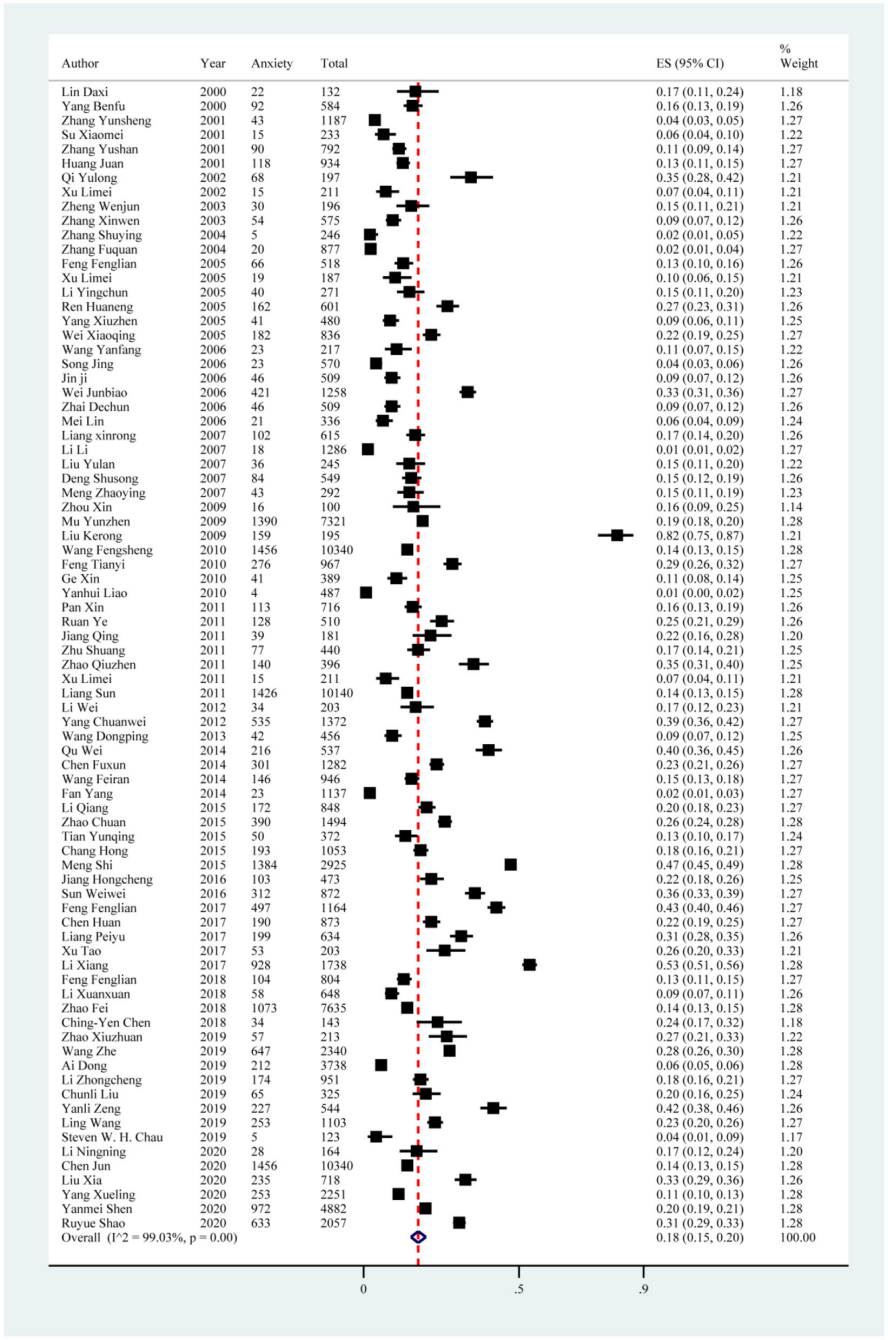
Forest plot of prevalence of anxiety in Chinese medical students.

The anxiety symptoms reported in the 80 included studies yielded a pooled prevalence of 18% (19,479/105,397; 95% CI: 15%−20%), with substantial evidence of between-study heterogeneity (*I*^2^ = 99.03%; Figure 4, Table 5). Sensitivity analysis showed that no individual study significantly affected the overall result (Supplementary material S5, Figure 2). In the subgroup analysis, heterogeneity was found to be reduced in the southwest region (*I*^2^ = 97.87%), south China (*I*^2^ = 86.94%), and in studies using SCL-90 with a score ≥ 3 (*I*^2^ = 77.66%; Table 5).

**Table 5:** Estimated anxiety prevalence among medical students in China.

**Subgroup**	**No. of studies**	**No. of anxiety**	**Sample size**	**Subgroup analysis**				**Meta-regression**	
				**Estimated rate (95% CI)**	***Q***	*I*^2^ **(%)**	***p*** **-value**	*I*^2^ **(%)**	***p*** **-value**
**Study region**									
Northeast	11	3,482	11,681	0.19 (0.09, 0.32)	2,385.60	99.58%	<0.01	99.25	0.6626
North China	10	1,130	5,258	0.18 (0.11, 0.27)	512.18	98.27%	<0.01		
East China	12	3,986	25,598	0.20 (0.16, 0.23)	381.33	97.12%	<0.01		
South China	9	804	6,069	0.12 (0.10, 0.15)	61.24	86.94%	<0.01		
Central China	11	2,803	14,682	0.15 (0.08, 0.23)	1,680.35	99.40%	<0.01		
Northwest	4	793	2,984	0.27 (0.23, 0.31)	20.60	85.43%	<0.01		
Southwest	6	2,580	11,651	0.24 (0.18, 0.31)	234.93	97.87%	<0.01		
Multiple regions	5	1,292	9,371	0.13 (0.06, 0.21)	179.87	97.78%	<0.01		
*N*	12	2,609	18,103	0.17 (0.11, 0.25)	1,199.99	99.08%	<0.01		
**Survey year**									
2000–2005	18	1,082	9,057	0.12 (0.08, 0.16)	540.21	96.85%	<0.01	99.21	0.0490
2005–2010	18	4,205	26,185	0.15 (0.10, 0.20)	1,583.38	98.93%	<0.01		
2010–2015	19	5,424	25,219	0.20 (0.15, 0.27)	2,294.95	99.22%	<0.01		
2015–2020	25	8,768	44,936	0.22 (0.18, 0.27)	3,125.67	99.23%	<0.01		
**Sample size**									
<200	10	420	1,618	0.23 (0.11, 0.37)	395.94	97.73%	<0.01	99.29	0.3992
201–400	16	653	4,363	0.14 (0.09, 0.18)	270.87	94.46%	<0.01		
401–600	15	1,249	7,741	0.14 (0.09, 0.21)	780.15	98.21%	<0.01		
601–800	7	959	4,724	0.20 (0.13, 0.27)	244.11	97.54%	<0.01		
801–1,000	10	1,694	8,908	0.18 (0.12, 0.25)	548.94	98.36%	<0.01		
>1,000	22	14,504	78,043	0.20 (0.15, 0.25)	5,750.08	99.63%	<0.01		
Simple	21	5,007	27,087	0.16 (0.12, 0.22)	2,464.46	99.19%	<0.01	99.25	0.3401
Convenience	2	1,225	7,133	0.17 (0.16, 0.18)	–	–	–		
Stratified	5	666	2,798	0.29 (0.13, 0.48)	430.75	99.07%	<0.01		
Cluster	20	5,299	23,598	0.19 (0.13, 0.25)	2,149.95	99.12%	<0.01		
Multiple sampling methods	23	4,835	29,037	0.18 (0.14, 0.23)	2,172.46	98.99%	<0.01		
*N*	9	2,447	15,744	0.12 (0.08, 0.17)	410.95	98.05%	<0.01		
**Educational level**									
Undergraduate	77	17,973	1,01,934	0.17 (0.15, 0.19)	6,828.34	98.89%	<0.01	99.20	0.1020
Postgraduate	3	1,506	3,463	0.31 (0.14, 0.51)	–	–	–		
**Measurement tool and cutoff score**									
BAI score ≥ 8	1	34	143	0.24 (0.17, 0.32)	–	–	–	98.94	0.0010
BAI score ≥ 10	2	2,882	20,480	0.14 (0.14, 0.15)	–	–	–		
BAI score ≥ 15	1	253	2,251	0.11 (0.10, 0.13)	–	–	–		
BAI score ≥ 50	1	50	372	0.13 (0.10, 0.17)	–	–	–		
DASS-21 score ≥ 8	2	480	1,647	0.29 (0.27, 0.31)	–	–	–		
GAD-7 score ≥ 10	1	65	325	0.20 (0.16, 0.25)	–	–	–		
GHQ-12 score ≥ 2	1	5	123	0.04 (0.01, 0.09)	–	–	–		
HAD score ≥ 9	1	39	181	0.22 (0.16, 0.28)	–	–	–		
HAMA score ≥ 7	1	159	195	0.82 (0.75, 0.87)	–	–	–		
HAMA score ≥ 14	2	318	1,152	0.27 (0.24, 0.29)	–	–	–		
MAS without cutoff score reported	1	54	575	0.09 (0.07, 0.12)	–	–	–		
S-AI without cutoff score reported	1	30	196	0.15 (0.11, 0.21)	–	–	–		
SAS without cutoff score reported	1	1,456	10,340	0.14 (0.13, 0.15)	–	–	–		
SAS score ≥ 40	3	197	1,790	0.11 (0.09, 0.13)	–	–	–		
SAS score ≥ 41	1	140	396	0.35 (0.31, 0.40)	–	–	–		
SAS score ≥ 47	3	151	976	0.15 (0.13, 0.18)	–	–	–		
SAS score ≥ 50	42	11,126	47,980	0.20 (0.17, 0.24)	4,378.44	99.06%	<0.01		
SAS score > 50	1	113	716	0.16 (0.13, 0.19)	–	–	–		
SAS score ≥ 51	1	68	197	0.35 (0.28, 0.42)	–	–	–		
SCARED score ≥ 23	1	41	389	0.11 (0.08, 0.14)	–	–	–		
SCL-90 score ≥ 1.8	1	1,390	7,321	0.19 (0.18, 0.20)	–	–	–		
SCL-90 score ≥ 2	3	264	1,698	0.16 (0.14, 0.17)	–	–	–		
SCL-90 score > 2	1	23	1,137	0.02 (0.01, 0.03)	–	–	–		
SCL-90 score ≥ 3	5	109	4,166	0.03 (0.02, 0.04)	17.91	77.66%	<0.01		
SIAS score ≥ 50	1	4	487	0.01 (0.00, 0.02)	–	–	–		
Self-made questions answers “yes”	1	28	164	0.17 (0.12, 0.24)	–	–	–		
Overall	80	19,479	1,05,397	0.18 (0.15, 0.20)	8,143.11	99.03%	<0.01		

N, not reported; BAI, Beck Anxiety Inventory; DASS-21, Depression Anxiety Stress Scale 21; GAD-7, Generalized Anxiety Disorder-7; GHQ-12, 12-item General Health Questionnaire; HAD, Hospital Anxiety and Depression Scale; HAMA, Hamilton Anxiety Scale; MAS, Manifest Anxiety Scale; S-AI, State-Anxiety Inventory; SAS, Self-Rating Anxiety Scale; SCARED, Rating Scale Scoring Aide; SCL-90, the symptom checklist-90; STAI-6, the 6-Item State Version of the State-Trait Anxiety Inventory.

**Figure 5:**
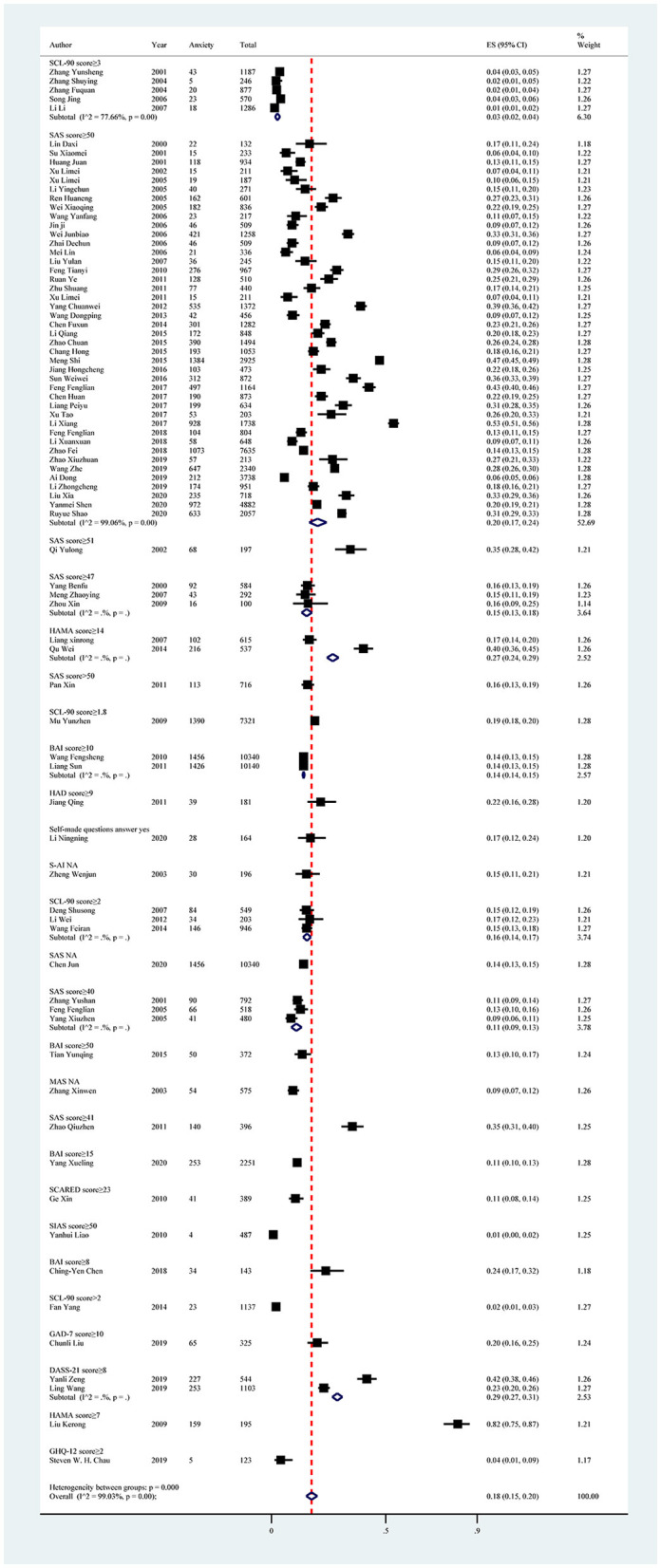
Subgroup analysis of anxiety in Chinese medical students based on measurements tools.

Subgroup analysis showed differences in prevalence based on study regions, survey years, sampling methods, measurement tools, and cutoff scores. Among all study regions, the estimated prevalence of anxiety symptoms was highest in the northwest region (27%; 95% CI: 23%−31%), followed by the southwest region (24%; 95% CI: 18%−31%). Furthermore, studies conducted between 2015 and 2020 showed a higher prevalence of anxiety symptoms (22%; 95% CI: 18%−27%) than other years. Among all sampling methods, the estimated prevalence of anxiety symptoms was highest in studies using stratified sampling methods (29%; 95% CI: 13%−48%), followed by cluster sampling methods (19%; 95% CI: 13%−25%). In terms of measurement tools and cutoff scores, the highest prevalence of anxiety symptoms was reported in the study using the Hamilton Depression Scale (HAMA) with a score ≥ 7 (82%; 95% CI: 75%−87%; Figure 5, Table 5).

In all univariate meta-regression analyses, only the measurement tool and cutoff score (*p* = 0.0010) could explain the heterogeneity between studies. Publication bias was found in the pooled prevalence analysis (*p* < 0.001 using Egger's test; Supplementary material S6, Figure 2).

### Suicidal behaviors

#### Suicidal ideation

**Figure 6:**
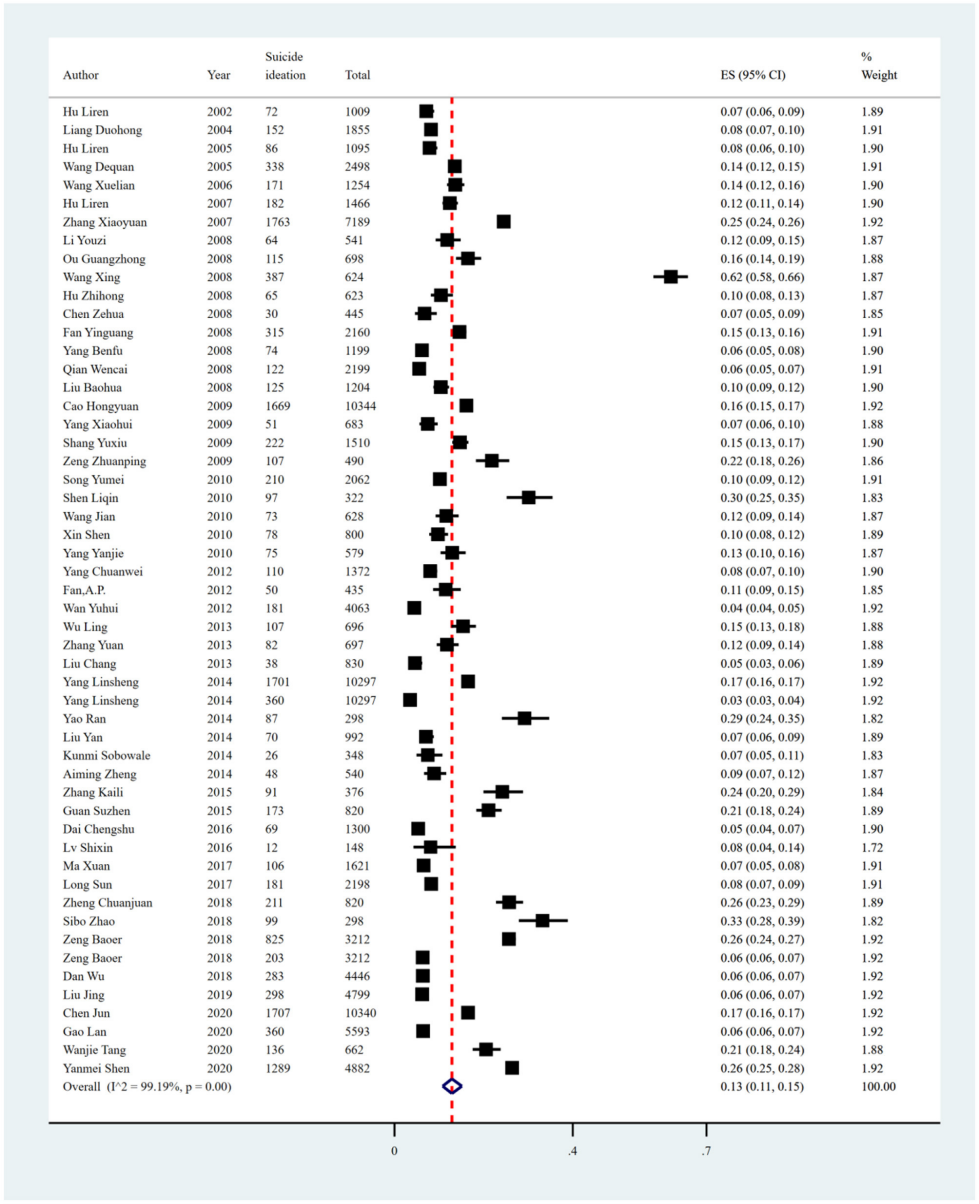
Forest plot of prevalence of suicidal ideation in Chinese medical students.

The pooled prevalence of suicide ideation reported in 53 studies was 13% (15,546/119,069, 95% CI: 11%−15%), with significant heterogeneity of 99.19% among included studies (Figure 6, Table 6). Sensitivity analysis showed that no individual study significantly affected the overall result (Supplementary material S5, Figure 3). In the subgroup analysis, heterogeneity was found to be reduced in the northeast region (*I*^2^ = 85.58%), recall period of the past 1 week (*I*^2^ = 84.33%), and in studies using the Self-rating Idea of Suicide Scale (SIOSS) to identify suicide ideation (*I*^2^ = 88.71%).

**Table 6:** Estimated suicide ideation prevalence among medical students in China.

**Subgroup**	**No. of studies**	**No. of suicide ideation**	**Sample size**	**Subgroup analysis**				**Meta-regression**	
				**Estimated rate (95% CI)**	***Q***	*I*^2^ **(%)**	***p*** **-value**	*I*^2^ **(%)**	***p*** **-value**
**Study region**									
Northeast	4	361	3,967	0.10 (0.07, 0.12)	20.81	85.58%	<0.01	99.14	0.8519
North China	2	247	3,403	0.07 (0.06, 0.08)	–	–	–		
East China	16	5,929	51,045	0.13 (0.09, 0.18)	2,844.04	99.47%	<0.01		
South China	6	3,015	15,052	0.17 (0.09, 0.26)	794.93	99.37%	<0.01		
Central China	3	1,490	6,630	0.19 (0.07, 0.34)	–	–	–		
Northwest	2	395	2,330	0.17 (0.15, 0.18)	–	–	–		
Southwest	2	133	1,380	0.10 (0.08, 0.11)	–	–	–		
Multiple regions	4	816	11,225	0.11 (0.07, 0.15)	152.41	97.38%	<0.01		
*N*	13	3,160	24,037	0.12 (0.09, 0.15)	575.80	97.92%	<0.01		
**Survey year**									
2000–2005	4	648	6,457	0.09 (0.06, 0.12)	53.76	94.42%	<0.01	99.08	0.6095
2005–2010	21	5,995	37,020	0.15 (0.11, 0.18)	1,642.94	98.78%	<0.01		
2010–2015	14	3,124	32,061	0.11 (0.08, 0.16)	1,547.63	99.16%	<0.01		
2015–2020	14	5,779	43,531	0.13 (0.09, 0.18)	2,352.25	99.45%	<0.01		
**Sample size**									
<200	1	12	148	0.08 (0.04, 0.14)	–	–	–	99.24	0.0686
201–400	5	400	1,642	0.24 (0.14, 0.35)	98.53	95.94%	<0.01		
401–600	6	374	3,030	0.12 (0.08, 0.16)	54.91	90.89%	<0.01		
601–800	9	1,094	6,111	0.17 (0.09, 0.27)	733.53	98.91%	<0.01		
801–1,000	4	492	3,462	0.13 (0.05, 0.25)	242.27	98.76%	<0.01		
>1,000	27	13,174	1,04,676	0.10 (0.08, 0.13)	4,980.92	99.46%	<0.01		
**Sampling methods**									
Simple	10	4,854	33,100	0.17 (0.12, 0.22)	1,373.24	99.27%	<0.01	98.96	0.2339
Convenience	1	1,289	4,882	0.26 (0.25, 0.28)	–	–	–		
Stratified	3	640	3,694	0.21 (0.12, 0.31)	–	–	–		
Cluster	10	3,090	32,989	0.08 (0.04, 0.14)	2,144.67	99.58%	<0.01		
Multiple	18	4,044	32,574	0.12 (0.10, 0.15)	681.49	97.51%	<0.01		
Multi-stage sampling	1	107	696	0.15 (0.13, 0.18)	–	–	–		
*N*	9	1,152	11,134	0.12 (0.07, 0.19)	669.67	98.81%	<0.01		
**Recall period**									
Past 1 week	4	671	5,460	0.12 (0.10, 0.15)	19.15	84.33%	<0.01	98.46	0.0583
Past 6 months	1	58	2,498	0.02 (0.02, 0.03)	–	–	–		
Past 1 year	18	2,495	36,144	0.10 (0.08, 0.12)	824.66	97.94%	<0.01		
Past 2 years	1	51	2,498	0.02 (0.02, 0.03)	–	–	–		
Lifetime	13	8,546	43,898	0.19 (0.15, 0.24)	1,383.14	99.13%	<0.01		
*N*	18	3,834	33,567	0.12 (0.09, 0.15)	1,068.59	98.41%	<0.01		
**Educational level**									
Undergraduate	51	15,096	1,12,897	0.13 (0.11, 0.15)	6,130.56	99.18%	<0.01	99.21	0.4261
Postgraduate/doctor	1	15	820	0.02 (0.01, 0.03)	–	–	–		
Unclassified	2	286	1,399	0.20 (0.18, 0.22)	–	–	–		
**Measurement tool**									
NCS	1	136	662	0.21 (0.18, 0.24)	–	–	–	99.26	0.0282
SBQ-R	2	1,028	6,424	0.15 (0.14, 0.16)	–	–	–		
QSA and Suicide ideation question	1	115	698	0.16 (0.14, 0.19)	–	–	–		
PHQ-9	2	386	5,941	0.06 (0.06, 0.07)	–	–	–		
BHS	1	48	540	0.09 (0.07, 0.12)	–	–	–		
SIOSS	6	432	4,898	0.09 (0.07, 0.12)	44.30	88.71%	<0.01		
BSI-CV	1	210	2,062	0.10 (0.09, 0.12)	–	–	–		
BSSI	2	384	3,460	0.11 (0.10, 0.12)	–	–	–		
PIL	1	91	376	0.24 (0.20, 0.29)	–	–	–		
EPQ	2	2,150	7,813	0.27 (0.26, 0.28)	–	–	–		
SIBQ	1	73	628	0.12 (0.09, 0.14)	–	–	–		
SSI	2	272	1,118	0.24 (0.22, 0.27)	–	–	–		
AHRBI	1	122	2,199	0.06 (0.05, 0.07)	–	–	–		
SCL-90	1	64	541	0.12 (0.09, 0.15)	–	–	–		
UPI	1	38	830	0.05 (0.03, 0.06)	–	–	–		
YRBSS	1	30	445	0.07 (0.05, 0.09)	–	–	–		
Medical Student Risk Behavior Questionnaire	1	125	1,204	0.10 (0.09, 0.12)	–	–	–		
Single item	1	283	4,446	0.06 (0.06, 0.07)					
Self-made questionnaire	25	9,559	74,784	0.13 (0.10, 0.16)	3,300.26	99.27%	<0.01		
Overall	53	15,546	119,069	0.13 (0.11, 0.15)	6,382.63	99.19%	<0.01		

N, not reported; NCS, National Comorbidity Survey; SBQ-R, The Suicide Behaviors Questionnaire-Revised; QSA, Suicide Attitude Questionnaire; PHQ-9, the Patient Health Questionnaire-9; BHS, Beck Hopelessness Scale; SIOSS, Self-Rating Idea of Suicide Scale; BSI-CV, Beck Scale for Suicide Ideation-Chinese Version; BSSI, Beck Scale for Suicidal Ideation; PIL, Purpose in Life Test; EPQ, Eysenck Personality Questionnaire; SIBQ, Suicidal Ideation and Behavior Questionnaire; SSI, Scale for Suicide Ideation; AHRBI, the Adolescent Health related Risky Behavior Inventory; SCL-90, the symptom checklist-90; UPI, University Personality Inventory; YRBSS, Youth Risk Behavior Surveillance System Questionnaire.

**Figure 7:**
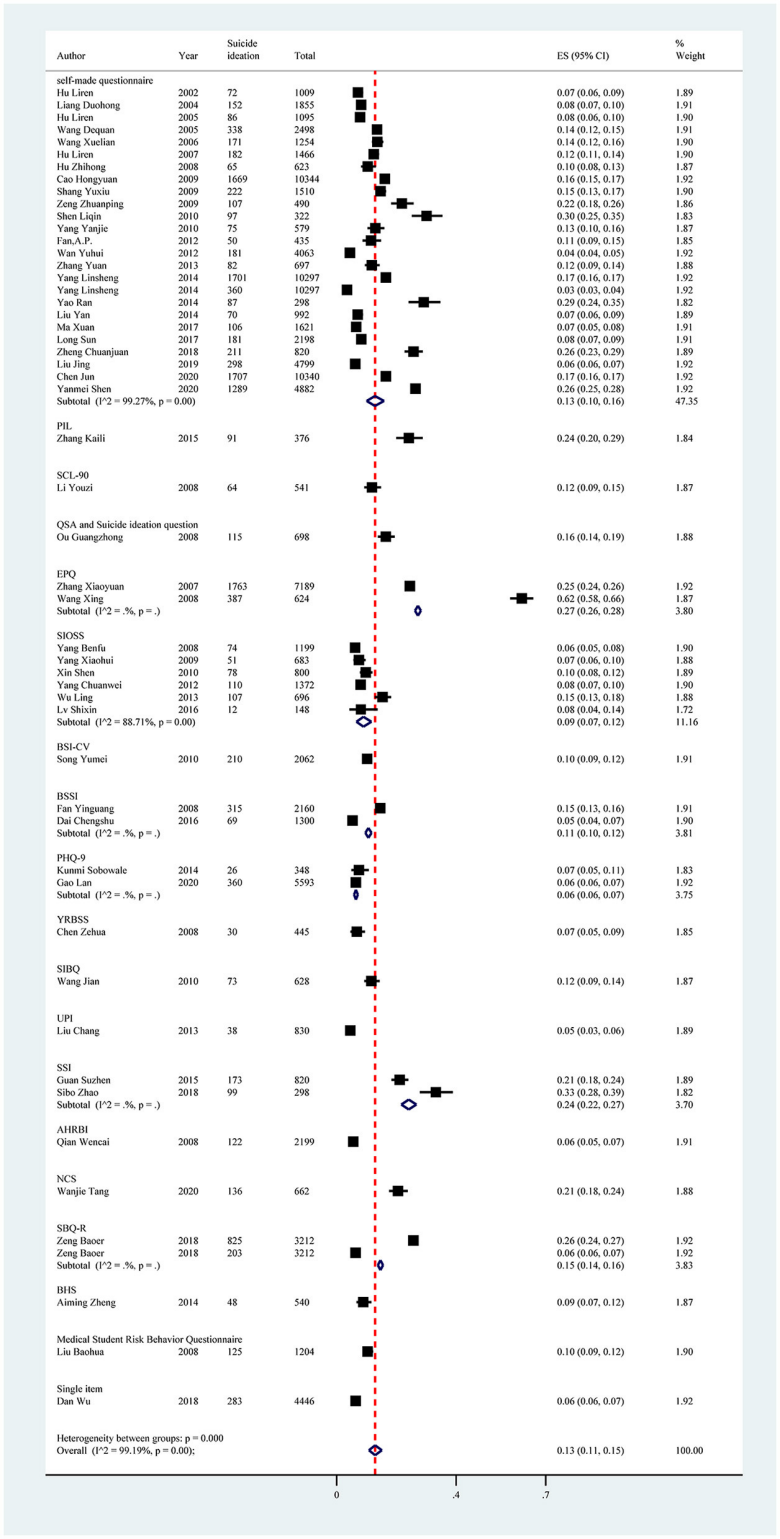
Subgroup analysis of suicide ideation in Chinese medical students based on measurements tools.

Subgroup analysis showed differences in prevalence based on study regions, sampling methods, recall periods, and measurement tools. The estimated prevalence of suicide ideation was highest in central China (19%; 95% CI: 7%−34%), followed by south China (17%, 95% CI: 9%−26%) and the southwest region (17%; 95% CI: 15%−18%). Furthermore, studies conducted between 2005 and 2010 had a higher prevalence of suicide ideation than other survey years (15%; 95% CI: 11%−18%). The estimated prevalence was higher in those studies using convenience sampling methods (26%; 95% CI: 25%−28%) compared with other sampling methods. Among all recall periods reported in the included studies, those studies using the recall period “lifetime” reported a higher estimated prevalence of suicide ideation (19%; 95% CI: 15%−24%). In terms of measurement tools, studies using the Eysenck Personality Questionnaire (EPQ), SSI, and Purpose in Life Test (PIL) reported higher pooled prevalence, with estimates of 27% (95% CI: 26%−28%), 24% (95% CI: 22%−27%), and 24% (95% CI: 20%−29%), respectively (Figure 7, Table 6).

Univariate meta-regression analyses demonstrated that measurement tools (*p* = 0.0282) could explain the potential source of the heterogeneity. Publication bias was found in the pooled prevalence analysis (*p* < 0.001 using Egger's test; Supplementary material S6, Figure 3).

#### Suicidal attempt

**Figure 8:**
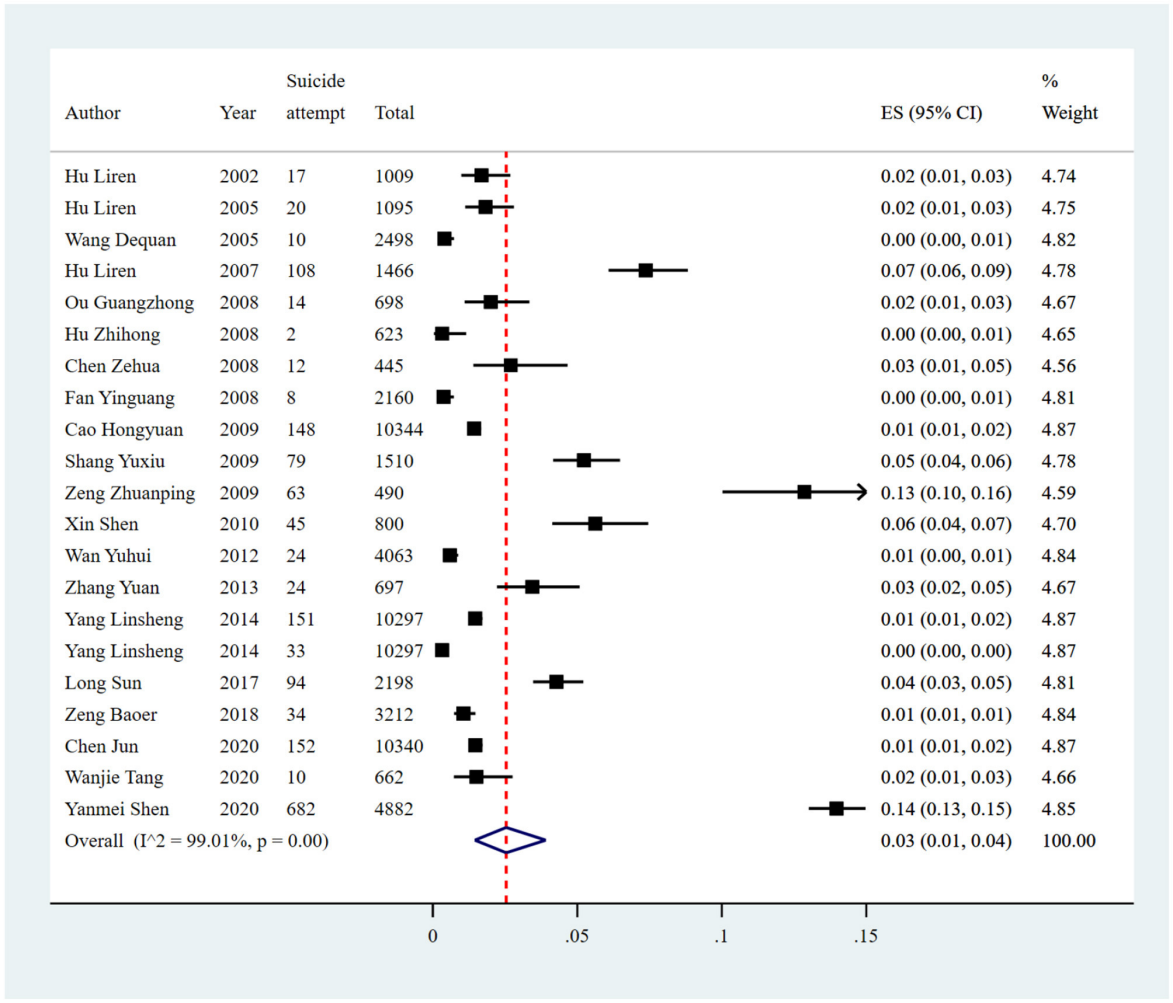
Forest plot of prevalence of suicidal attempt in Chinese medical students.

The pooled prevalence of suicide attempts reported in 21 studies was 3% (1,730/69,786, 95% CI: 1%−4%), with significant heterogeneity of 99.01% among the included studies (Figure 8, Table 7). Sensitivity analysis showed that no individual study significantly affected the overall result (Supplementary material S5, Figure 4).

**Table 7:** Estimated suicide attempt prevalence among medical students in China.

**Subgroup**	**No. of studies**	**No. of suicide attempt**	**Sample size**	**Subgroup analysis**				**Meta-regression**	
				**Estimated rate (95% CI)**	***Q***	*I*^2^ **(%)**	***p*** **-value**	*I*^2^ **(%)**	***p*** **-value**
**Study region**									
East China	8	425	39,282	0.01 (0.01, 0.02)	203.81	96.57%	<0.01	96.45	0.0294
South China	2	46	3,657	0.01 (0.01, 0.02)	–	–	–		
Central China	1	682	4,882	0.14 (0.13, 0.15)	–	–	–		
Northwest	1	79	1,510	0.05 (0.04, 0.06)	–	–	–		
Southwest	1	24	697	0.03 (0.02, 0.05)	–	–	–		
*N*	8	474	19,758	0.03 (0.01, 0.05)	321.29	97.82%	<0.01		
**Survey year**									
2000–2005	3	47	4,602	0.01 (0.00, 0.03)	–	–	–	98.39	0.4842
2005–2010	9	479	18,536	0.03 (0.02, 0.06)	373.32	97.86%	<0.01		
2010–2015	4	232	25,354	0.01 (0.00, 0.02)	115.28	97.40%	<0.01		
2015–2020	5	972	21,294	0.04 (0.01, 0.09)	1,030.54	99.61%	<0.01		
**Sample size**									
<600	2	75	935	0.07 (0.06, 0.09)	–	–	–	98.52	0.2902
601–800	5	95	3,480	0.02 (0.01, 0.04)	50.15	92.02%	<0.01		
>1,000	14	1,560	65,371	0.02 (0.01, 0.04)	1,855.03	99.30%	<0.01		
**Sampling methods**									
Simple	4	403	23,501	0.02 (0.01, 0.03)	61.58	95.13%	<0.01	95.96	0.0402
Convenience	1	682	4,882	0.14 (0.13, 0.15)	–	–	–		
Stratified	1	10	2,498	0.00 (0.00, 0.01)	–	–	–		
Cluster	5	128	16,303	0.02 (0.01, 0.04)	129.61	96.91%	<0.01		
Multiple	8	456	18,381	0.03 (0.01, 0.06)	345.48	97.97%	<0.01		
*N*	2	51	4,221	0.01 (0.01, 0.02)	–	–	–		
**Recall period**									
Past 1 week	2	32	2,857	0.01 (0.01, 0.01)	–	–	–	98.41	0.1190
Past 1 month	1	63	490	0.13 (0.10, 0.16)	–	–	–		
Past 1 year	8	271	20,807	0.02 (0.01, 0.03)	309.87	97.74%	<0.01		
Lifetime	6	1,133	32,699	0.03 (0.01, 0.07)	1,308.39	99.62%	<0.01		
*N*	4	231	12,933	0.02 (0.01, 0.04)	42.70	92.97%	<0.01		
**Educational level**									
Undergraduate	21	1,730	69,786	0.03 (0.01, 0.04)	2,022.20	99.01%	<0.01	-	-
**Measurement tool**									
NCS	1	10	662	0.02 (0.01, 0.03)	–	–	–	98.82	0.9576
QSA and Suicide ideation question	1	14	698	0.02 (0.01, 0.03)	–	–	–		
BSSI	1	8	2,160	0.00 (0.00, 0.01)	–	–	–		
SBQ-R	1	34	3,212	0.01 (0.01, 0.01)	–	–	–		
SIOSS	1	45	800	0.06 (0.04, 0.07)	–	–	–		
Self-made questionnaire	15	1,607	61,809	0.03 (0.01, 0.05)	1,924.10	99.27%	<0.01		
YRBSS	1	12	445	0.03 (0.01, 0.05)	–	–	–		
Overall	21	1,730	69,786	0.03 (0.01, 0.04)	2,022.20	99.01%	<0.01		

N, not reported; NCS, National Comorbidity Survey; QSA, Suicide Attitude Questionnaire; SBQ-R, The Suicide Behaviors Questionnaire-Revised; SIOSS, Self-rating Idea of Suicide Scale; YRBSS, Youth Risk Behavior Surveillance System.

**Figure 9:**
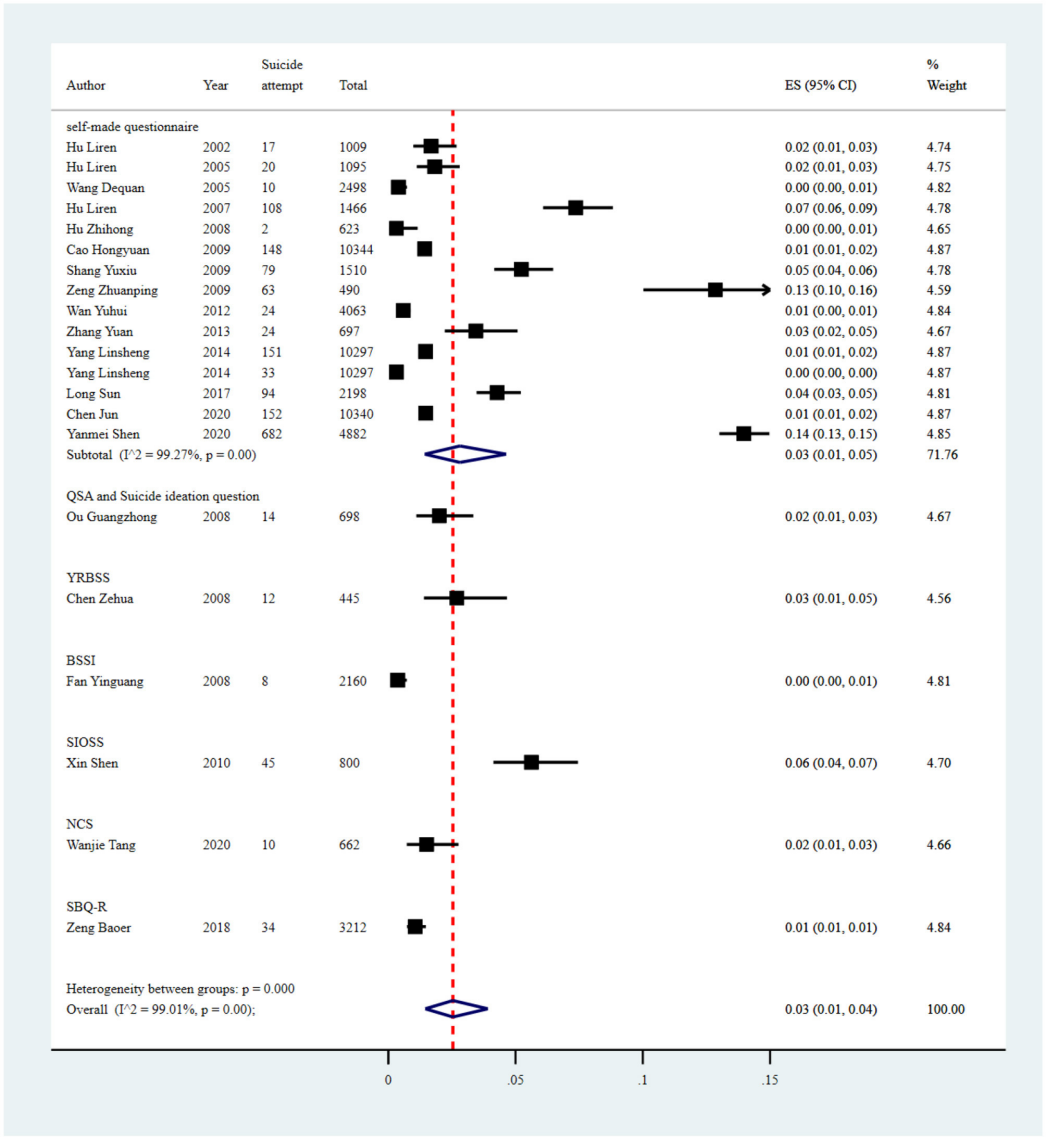
Subgroup analysis of suicide attempt in Chinese medical students based on measurements tools.

Subgroup analysis showed differences in prevalence based on study regions, survey years, sampling methods, recall periods, and measurement tools. The estimated prevalence of suicide attempt was higher in central China (14%; 95% CI: 13%−15%) than other regions. Studies conducted between 2015 and 2020 (4%; 95% CI: 1%−9%) had a higher prevalence of suicide attempt than other survey years. Furthermore, the estimated prevalence was higher in those studies using convenience sampling methods (14%; 95% CI: 13%−15%) than other sampling methods. The studies with a recall period of the past 1 month reported a significantly higher pooled prevalence (13%; 95% CI: 10%−16%) than other recall periods. As for measurement tools, the studies using SIOSS reported a higher pooled prevalence of suicide attempt, with an estimate of 6% (95% CI: 4%−7%; Figure 9, Table 7).

Univariate meta-regression analyses demonstrated that study region (*p* = 0.0294) and sampling method (*p* = 0.0402) could explain the potential source of the heterogeneity. Publication bias was found in the pooled prevalence analysis (*p* < 0.001 using Egger's test; Supplementary material S6, Figure 4).

#### Suicidal plan

**Figure 10:**
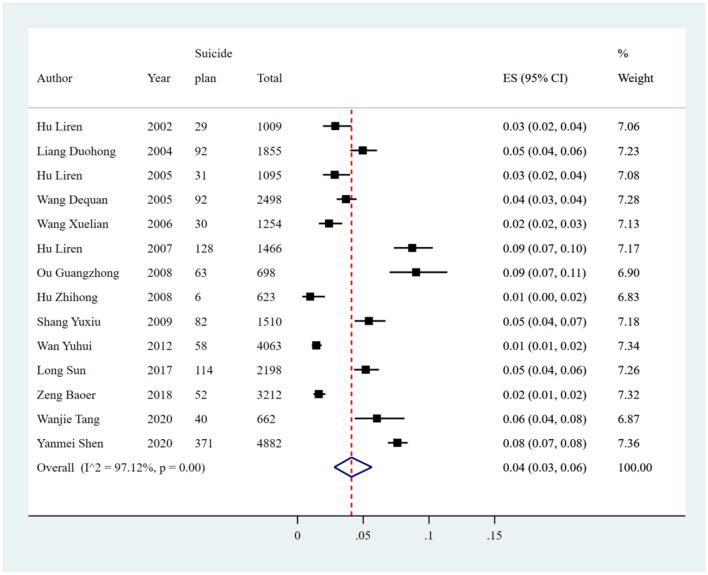
Forest plot of prevalence of suicidal plan in Chinese medical students.

The pooled prevalence of suicide plan reported in 14 studies was 4% (1,188/27,025, 95% CI: 3%−6%), with significant heterogeneity of 97.12% among the included studies (Figure 10, Table 8). Sensitivity analysis showed that no individual study significantly affected the overall result (Supplementary material S5, Figure 5). In the subgroup analysis, heterogeneity was found to be reduced in the survey years from 2000 to 2005 (*I*^2^ = 74.16%).

**Table 8:** Estimated suicide plan prevalence among medical students in China.

**Subgroup**	**No. of studies**	**No. of suicide plan**	**Sample size**	**Subgroup analysis**				**Meta-regression**	
				**Estimated rate (95% CI)**	***Q***	*I*^2^ **(%)**	***p*** **-value**	*I*^2^ **(%)**	***p*** **-value**
**Study region**									
Northeast	1	92	1,855	0.05 (0.04, 0.06)	–	–	–	93.07	0.6759
East China	4	157	6,638	0.03 (0.01, 0.06)	87.76	96.58%	<0.01		
South China	1	52	3,212	0.02 (0.01, 0.02)	–	–	–		
Central China	1	371	4,882	0.08 (0.07, 0.08)	–	–	–		
Northwest	1	82	1,510	0.05 (0.04, 0.07)	–	–	–		
*N*	6	434	8,928	0.05 (0.03, 0.07)	68.38	92.69%	<0.01		
**Survey year**									
2000–2005	4	244	6,457	0.04 (0.03, 0.05)	11.61	74.16%	0.01	97.19	0.5487
2005–2010	5	309	5,551	0.05 (0.02, 0.08)	113.35	96.47%	<0.01		
2010–2015	1	58	4,063	0.01 (0.01, 0.02)	–	–	–		
2015–2020	4	577	10,954	0.05 (0.02, 0.09)	180.12	98.33%	<0.01		
**Sample size**									
601–800	3	109	1,983	0.05 (0.01, 0.11)	–	–	–	97.29	0.614
>1,000	11	1,079	25,042	0.04 (0.03, 0.06)	389.51	97.43%	<0.01		
**Sampling methods**									
Simple	3	184	4,114	0.04 (0.02, 0.07)	–	–	–	95.81	0.7784
Convenience	1	371	4,882	0.08 (0.07, 0.08)	–	–	–		
Stratified	1	92	2,498	0.04 (0.03, 0.04)	–	–	–		
Cluster	2	121	4,761	0.02 (0.02, 0.03)		–	–		
Multiple	5	339	6,549	0.04 (0.02, 0.07)	84.78	95.28%	<0.01		
*N*	2	81	4,221	0.02 (0.01, 0.02)	–	–	–		
**Recall period**									
During college	1	30	1,254	0.02 (0.02, 0.03)	–	–	–	97.56	0.6329
Past 1 year	7	421	11,920	0.04 (0.02, 0.05)	142.20	95.78%	<0.01		
Lifetime	4	643	12,058	0.05 (0.02, 0.09)	221.57	98.65%	<0.01		
Undergraduate	14	1,188	27,025	0.04 (0.03, 0.06)	450.90	97.12%	<0.01	–	–
**Measurement tool**									
NCS	1	40	662	0.06 (0.04, 0.08)	–	–	–	97.25	0.2418
QSA and suicide ideation question	1	63	698	0.09 (0.07, 0.11)	–	–	–		
SBQ-R	1	52	3,212	0.02 (0.01, 0.02)	–	–	–		
Self-made questionnaire	11	1,033	22,453	0.04 (0.03, 0.06)	340.98	97.07%	<0.01		
Overall	14	1,188	27,025	0,04 (0.03, 0.06)	450.90	97.12%	<0.01		

N, not reported; NCS, National Comorbidity Survey; QSA, Suicide Attitude Questionnaire; SBQ-R, The Suicide Behaviors Questionnaire-Revised.

**Figure 11:**
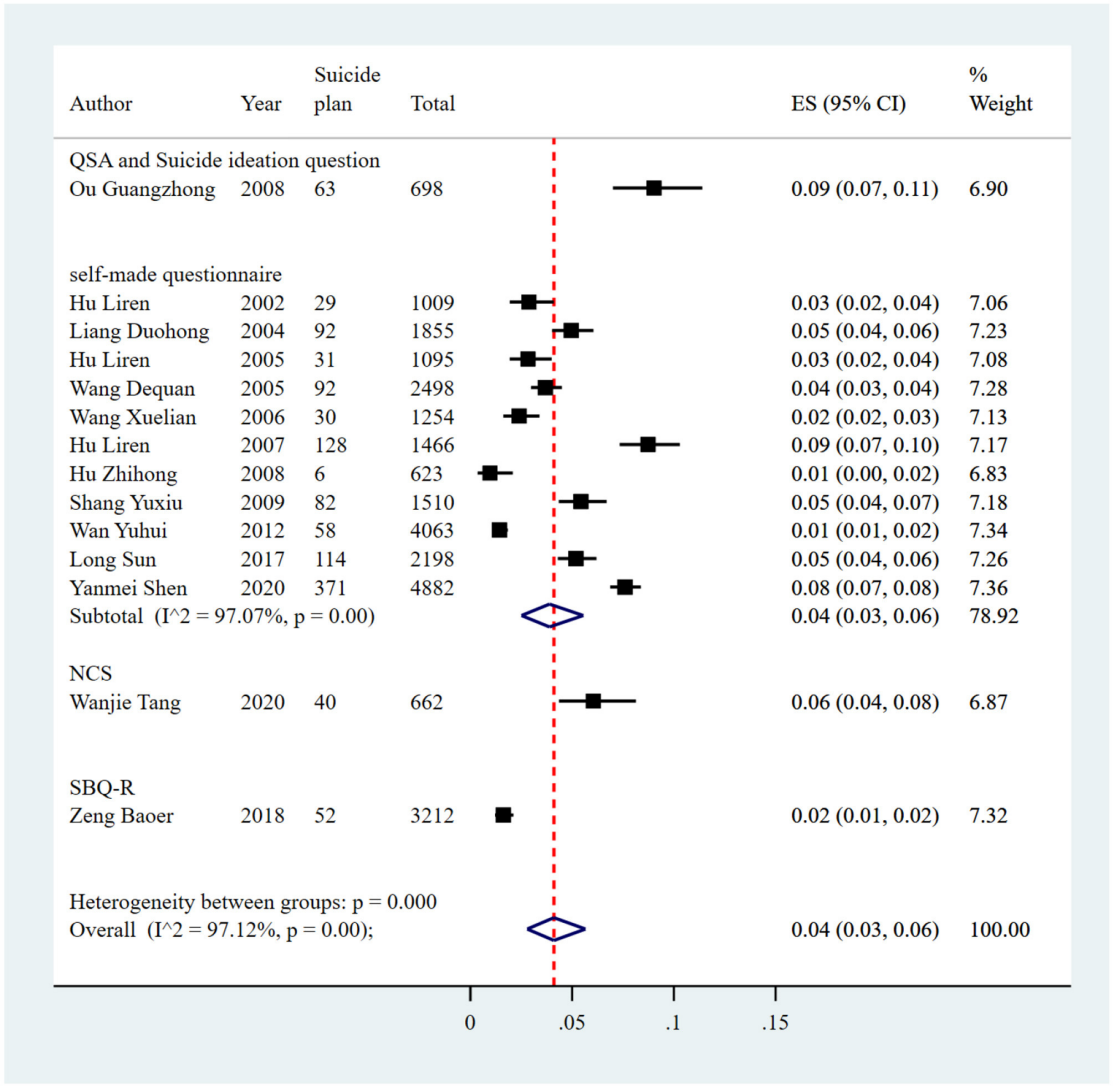
Subgroup analysis of suicide plan in Chinese medical students based on measurements tools.

Subgroup analysis showed differences in prevalence based on study regions, survey years, sampling methods, and measurement tools. The estimated prevalence of suicide attempt was higher in central China (8%; 95% CI: 7%−8%). Additionally, studies conducted between 2010 and 2015 had the lowest prevalence of suicide attempt (1%; 95% CI: 1%−2%) among all survey years. The estimated prevalence was higher in those studies using convenience sampling methods (8%; 95% CI: 7%−8%) than other sampling methods. Among all measurement tools, studies using the Questionnaire of Suicide Attitude (QSA) and Suicide Ideation Question reported a higher prevalence (9%; 95% CI: 7%−11%; Figure 11, Table 8).

Significant results were not found in all univariate meta-regression analyses to explain the heterogeneity between studies. Publication bias was found in the pooled prevalence analysis (*p* < 0.001 using Egger's test; Supplementary material S6, Figure 5).

## Discussion

### Summary of results

To the best of our knowledge, this is the most comprehensive systematic review and meta-analysis to estimate the prevalence of CMDs among Chinese medical students. Our study revealed that the pooled prevalence of depression, anxiety, suicidal ideation, suicidal attempt, and suicidal plans was 29%, 17%, 13%, 3%, and 4%, respectively. The high prevalence values emphasize the need for CMD prevention and intervention for Chinese medical students.

### Depression

Our study demonstrated a pooled prevalence of depressive symptoms among Chinese medical students of 29%, which was higher than that for general university students (24.4%) in low- and middle-income countries (LMICs) (40) and previously reported studies (28.4 and 23.8%) in China (41, 42). This may be because medical students may experience higher academic pressure due to the arduous training curriculum, less time for relaxing or seeking psychological help (18, 43), and employment stress since pursuing a master's or even doctoral degree is commonly required to enter a hospital in China (44). These two factors are unique to medical students (45). Furthermore, our results revealed that the prevalence of depression symptoms among Chinese medical students was higher than the global prevalence in medical students (28.0%) (46). This finding could be the result of cultural differences among different countries. Compared with Western countries, Asian countries with a prominent Confucian Heritage Culture, such as China, emphasize academic excellence starting at a young age (47). Such high expectations often result in excessive pressure on students, which could influence their psychological wellbeing. In this situation, students, especially medical students, who bear more stressors from clinical curriculums and trainings, might report higher levels of depression.

The prevalence of depression in our study was similar to that reported by resident physicians worldwide (28.8%) (15), which suggested that depression was a problem affecting all levels of medical training. However, the result of our study was lower than that found in nursing students (34.0%) of similar age and education level. The possible explanation is that nursing has been a female-dominated profession for decades, and it has been confirmed that women tend to be more commonly affected by mental disorders than men (48).

Thus, it is suggested that more attention should be paid to medical students with signs and symptoms of depression, and timely screening and proper interventions are highly necessary.

### Anxiety

This study demonstrated that the pooled prevalence of anxiety was 18%, which was much higher than that for Asian medical students (7.04%) (49). Interestingly, our result was lower than the prevalence of anxiety worldwide and even in other LMICs. For example, previous research has shown a pooled prevalence of anxiety among medical students of 33.8% worldwide (14), 32.9% in Brazil (50), and 34.5% in India (51). Different medical education systems and healthcare working environments among different countries could explain the discrepancies found in different areas.

However, anxiety among medical students was much higher than in the general population. Available data suggest that the prevalence of depressive and anxiety disorders in the general population ranges from 5 to 7% worldwide (52, 53). The long-term heavy academic burden (1), high intensity internships (2), complex doctor-patient relationships (54), and future uncertainty (5) could result in a higher prevalence of anxiety among medical students than the general population. Like depression, persistent anxiety symptoms could also lead to many undesirable consequences, such as poor academic performance, impaired cognitive function, burnout, and even suicidality (18, 55, 56). Thus, the anxiety in this population should be taken seriously and prevented effectively.

### Suicidal behaviors

This study identified that the pooled prevalence of suicide ideation, suicide attempt, and suicide plan was 13%, 3%, and 4%, respectively. The pooled prevalence of suicide ideation in this study was similar to the global pooled prevalence (11.1%) and the pooled prevalence in China published in previous studies (11%) (10, 28). Furthermore, the pooled prevalence of suicide plan was also similar to the results of a Chinese language meta-analysis, which demonstrated that 4.4% of medical students reported suicidal plans (57).

When compared with physicians worldwide, minor differences were found between our findings and a previous meta-analysis. In this study, the summarized life-time prevalence of suicidal ideation was 17.4%, while the 1-year prevalence was 8.6% and the 6-month prevalence was 11.9%. With respect to suicidal attempt, the lifetime prevalence was 1.8%, while the 1-year prevalence was 0.3% (58). Combined with the above results, Chinese medical students in our study were less likely to report suicidal ideation (2% in recent 6 months) but more likely to report suicidal attempt (2% in recent 1 year) than physicians in recent recall periods.

These results suggested that Chinese medical students, similar to other populations with clinical training (such as physicians), had a higher risk for suicide-related thoughts and behaviors. The possible reasons might be a high rate of depression, work burnout, medical adverse events and errors, and a lower likelihood of seeking psychological help among medical students and physicians (10, 59, 60). Effective preventive efforts and the accessibility of mental health services for medical students should be developed in the future.

## Limitations of this review and included studies

Our study has some limitations. First, the data were mostly derived from studies with a cross-sectional design, which limited a dynamic analysis of mental distress in this meta-analysis. Second, the data from different specialties (e.g., clinical medicine, dental medicine, preventive medicine, and nursing) and grades could not be extracted for final analysis, leaving substantial heterogeneity among studies unexplained. Third, it was impossible to perform a gender analysis since many studies did not provide separate prevalences of mental disorders for men and women. Fourth, a wide variety of screening instruments with different cutoff scores for mental distress were used in different studies, resulting in high heterogeneity across individual studies. Fifth, current studies on mental distress among Chinese medical students focused on limited mental problems. The investigation of other mental distresses such as obsessive-compulsive disorder, irritable bowel syndrome, bipolar disorders, and combinations of these was lacking in most studies. Finally, publication bias existed in our study, and the results should be interpreted with caution.

## Implications for further research

Most included studies used a cross-sectional design with small sample sizes, which limits the generalization of the results to a wider population. Thus, future research should include prospective, randomized, multicenter studies with larger sample sizes. Additionally, most included studies solely focused on major mental health problems, such as depression, anxiety, and suicidal behaviors. Future studies should investigate other mental health disorders, such as bipolar, obsessive-compulsive, and eating disorders, alone and in combination. More subgroup and stratified analyses are also suggested to identify the prevalence of mental health problems in different subgroups of Chinese medical students, such as different grades, to provide targeted and personalized intervention programs. Finally, more interventional studies are needed to find ways to address the poor mental health of this population.

## Implications for practice

Given the high prevalence of mental health disorders among medical students, there is a pressing need for further research utilizing standardized screening instruments with valid cutoff scores to accurately assess those disorders. It is suggested that medical schools implement regular monitoring of students' psychological wellbeing and establish comprehensive psychological interventions or programs that have demonstrated effectiveness in reducing students' mental health disorders. For instance, organizing structured programs with validated approaches like life skills training (61) and mindfulness therapy (62) could be implemented for medical students experiencing anxiety. Additionally, providing mental support within the college setting, including mental health-related courses and accessible counseling centers, is essential (26). Furthermore, continuous efforts are necessary to destigmatize mental health issues among medical students and promote a culture of help-seeking behavior. Medical schools can play a vital role in this by explicitly stating that having mental health problems will not result in demerit points or negative consequences for students. Sharing the successful experiences of senior doctors in managing mental health challenges may also encourage medical students to approach their own mental health struggles more positively (14). By prioritizing standardized assessments, implementing evidence-based interventions, and fostering a supportive environment, medical schools can actively address the mental health needs of their students. This multifaceted approach can not only alleviate the burden of mental health disorders but also create a positive and thriving learning environment for future healthcare professionals.

## Conclusion

Our findings showed that Chinese medical students had a high level of depression, anxiety, and suicidal behaviors. Thus, timely screening and targeted intervention programs in this population to improve their mental health are needed. However, high heterogeneity and publication bias across the included studies were found in this review, suggesting that the results should be interpreted with caution.

## Data availability statement

The original contributions presented in the study are included in the article/Supplementary material, further inquiries can be directed to the corresponding author.

## Author contributions

PX: conceptualization, data curation, formal analysis, funding acquisition, investigation, methodology, project administration, resources, software, supervision, validation, visualization, writing—original draft, and writing—review and editing. JWa, ML, and JB: data curation, formal analysis, investigation, methodology, software, visualization, and writing—original draft. YC, BL, and RW: writing—original draft. JL and JWu: data curation, investigation, and methodology. All authors contributed to the article and approved the submitted version.
