# Supplementary material for: Prevalence of common mental disorders among medical students in China: a systematic review and meta-analysis
Source: Front Public Health. 2023 Aug 31;11:1116616. doi: 10.3389/fpubh.2023.1116616 (PMC10501456; doi:10.3389/fpubh.2023.1116616)
Supplement: Supplementary file 1 [file Data_Sheet_1.docx]

**Support InformationS_1: Quality assessment for quantitative studies**

**S_1.1 Quality assessment for quantitative studies (n=197)**

| **1st Author (Year)** | **Item 1** | **Item 2** | **Item 3** | **Item 4** | **Item 5** | **Item 6** | **Item 7** | **Item 8** | **Item 9** | **Item 10** | **Total Score** |
| --- | --- | --- | --- | --- | --- | --- | --- | --- | --- | --- | --- |
| Lin Daxi (2000) | 2 | 1 | 2 | 2 | 2 | 2 | 1 | 2 | 2 | 1 | 17 |
| Du Zhaoyun (2000) | 2 | 1 | 0 | 2 | 2 | 2 | 1 | 2 | 2 | 2 | 16 |
| Wu Hualin (2000) | 2 | 1 | 2 | 2 | 1 | 1 | 1 | 2 | 2 | 1 | 15 |
| Yang Benfu (2000) | 2 | 2 | 1 | 1 | 0 | 1 | 1 | 2 | 2 | 2 | 14 |
| Huang Juan (2001) | 2 | 2 | 2 | 2 | 1 | 2 | 1 | 2 | 2 | 2 | 18 |
| Yu Miao (2001) | 2 | 2 | 2 | 2 | 1 | 2 | 1 | 2 | 1 | 2 | 17 |
| Lin Zhiping (2001) | 2 | 2 | 1 | 1 | 1 | 2 | 1 | 2 | 2 | 1 | 15 |
| Su Xiaomei (2001) | 1 | 2 | 1 | 1 | 1 | 1 | 1 | 2 | 2 | 1 | 13 |
| Zhang Yunsheng (2001) | 2 | 1 | 1 | 1 | 1 | 2 | 1 | 1 | 1 | 1 | 12 |
| Zhang Yushan (2001) | 1 | 1 | 0 | 0 | 1 | 1 | 1 | 2 | 1 | 1 | 9 |
| Qi Yulong (2002) | 2 | 1 | 1 | 1 | 1 | 1 | 1 | 1 | 2 | 1 | 12 |
| Hu Liren (2002) | 2 | 2 | 1 | 1 | 0 | 1 | 1 | 2 | 1 | 1 | 12 |
| Rao Hong (2002) | 0 | 1 | 0 | 0 | 0 | 0 | 1 | 2 | 1 | 1 | 6 |
| Zhou Rong (2003) | 2 | 1 | 0 | 2 | 2 | 1 | 1 | 2 | 2 | 2 | 15 |
| Zhang Xinwen (2003) | 2 | 2 | 1 | 1 | 0 | 1 | 1 | 2 | 1 | 2 | 13 |
| Wang Menglong (2003) | 2 | 1 | 1 | 0 | 1 | 0 | 1 | 2 | 1 | 1 | 10 |
| Gesang Zeren (2003) | 1 | 1 | 0 | 1 | 1 | 0 | 1 | 2 | 1 | 1 | 9 |
| Zheng Wenjun (2003) | 1 | 1 | 0 | 1 | 0 | 0 | 1 | 1 | 1 | 0 | 6 |
| Zhang Fuquan (2004) | 2 | 2 | 1 | 1 | 1 | 1 | 1 | 2 | 2 | 2 | 15 |
| Liang Duohong (2004) | 2 | 2 | 1 | 2 | 1 | 1 | 1 | 2 | 1 | 1 | 14 |
| Zhang Shuying (2004) | 1 | 1 | 0 | 1 | 0 | 1 | 1 | 2 | 2 | 1 | 10 |
| Shi Xiaoning (2005) | 2 | 2 | 2 | 2 | 2 | 2 | 1 | 2 | 2 | 2 | 19 |
| Gesang Zeren (2005) | 2 | 2 | 2 | 2 | 1 | 2 | 1 | 2 | 2 | 1 | 17 |
| Hu Liren (2005) | 2 | 2 | 1 | 2 | 1 | 1 | 1 | 2 | 2 | 2 | 16 |
| Ren Huaneng (2005) | 2 | 2 | 1 | 2 | 1 | 1 | 1 | 2 | 1 | 2 | 15 |
| Li Yingchun (2005) | 2 | 2 | 1 | 1 | 1 | 1 | 1 | 2 | 2 | 1 | 14 |
| Guo Rong (2005) | 2 | 1 | 1 | 2 | 1 | 1 | 1 | 2 | 2 | 1 | 14 |
| Xu Limei (2005) | 2 | 1 | 1 | 0 | 1 | 1 | 1 | 2 | 2 | 2 | 13 |
| Wang Dequan (2005) | 2 | 1 | 2 | 2 | 0 | 1 | 1 | 2 | 1 | 1 | 13 |
| Yang Xiuzhen (2005) | 2 | 1 | 1 | 1 | 0 | 1 | 1 | 2 | 2 | 1 | 12 |
| Wei Xiaoqing (2005) | 2 | 1 | 1 | 1 | 1 | 1 | 1 | 2 | 1 | 1 | 12 |
| Fen Fenglian (2005) | 2 | 1 | 0 | 0 | 0 | 0 | 1 | 2 | 1 | 1 | 8 |
| Wang Xuelian (2006) | 2 | 2 | 2 | 2 | 2 | 2 | 1 | 2 | 2 | 2 | 19 |
| Jin ji (2006) | 2 | 2 | 1 | 2 | 1 | 2 | 1 | 2 | 2 | 2 | 17 |
| Xu Minlan (2006) | 2 | 2 | 2 | 2 | 1 | 2 | 1 | 1 | 2 | 1 | 16 |
| Zhang Zewu (2006) | 1 | 1 | 1 | 2 | 1 | 2 | 1 | 2 | 2 | 2 | 15 |
| Zhai Dechun (2006) | 2 | 2 | 1 | 1 | 1 | 1 | 1 | 2 | 2 | 2 | 15 |
| Wei Junbiao (2006) | 2 | 1 | 1 | 2 | 1 | 1 | 1 | 2 | 2 | 1 | 14 |
| Zeng Qiang (2006) | 2 | 1 | 1 | 0 | 1 | 1 | 1 | 2 | 2 | 2 | 13 |
| Wang Yanfang (2006) | 2 | 1 | 0 | 1 | 1 | 1 | 1 | 2 | 2 | 1 | 12 |
| Zhang Zewu (2006) | 1 | 1 | 0 | 2 | 1 | 1 | 1 | 2 | 2 | 1 | 12 |
| Mei Lin (2006) | 2 | 1 | 1 | 1 | 1 | 0 | 1 | 2 | 2 | 1 | 12 |
| Song Jing (2006) | 1 | 1 | 1 | 2 | 1 | 0 | 1 | 2 | 1 | 1 | 11 |
| Wu Yan (2006) | 2 | 1 | 0 | 1 | 0 | 1 | 1 | 2 | 1 | 1 | 10 |
| Wang Tao (2007) | 2 | 2 | 1 | 2 | 1 | 1 | 1 | 2 | 1 | 2 | 15 |
| Hu Liren (2007) | 2 | 2 | 1 | 0 | 1 | 1 | 1 | 2 | 2 | 2 | 14 |
| Zhang Xiaoyuan (2007) | 2 | 2 | 1 | 1 | 1 | 1 | 1 | 2 | 2 | 1 | 14 |
| Liang Xinrong (2007) | 2 | 2 | 0 | 2 | 1 | 1 | 1 | 2 | 1 | 1 | 13 |
| Deng Shugong (2007) | 2 | 1 | 1 | 1 | 1 | 1 | 1 | 2 | 2 | 1 | 13 |
| Sang Wenhua (2007) | 1 | 1 | 1 | 1 | 1 | 1 | 1 | 2 | 2 | 1 | 12 |
| Liu Yulan (2007) | 2 | 1 | 0 | 1 | 0 | 1 | 1 | 2 | 1 | 1 | 10 |
| Li Li (2007) | 1 | 1 | 0 | 0 | 0 | 1 | 1 | 2 | 1 | 2 | 9 |
| Ou Guangzhong (2008) | 2 | 1 | 2 | 2 | 2 | 2 | 1 | 2 | 2 | 2 | 18 |
| Fan Yinguang (2008) | 2 | 2 | 2 | 2 | 1 | 1 | 1 | 2 | 2 | 2 | 17 |
| Wang Xin (2008) | 2 | 1 | 2 | 1 | 2 | 2 | 1 | 2 | 2 | 1 | 16 |
| Hu Zhihong (2008) | 2 | 1 | 1 | 2 | 1 | 2 | 1 | 2 | 2 | 2 | 16 |
| Du Juan (2008) | 2 | 2 | 1 | 1 | 2 | 1 | 1 | 2 | 2 | 2 | 16 |
| Yang Benfu (2008) | 2 | 2 | 2 | 0 | 1 | 2 | 1 | 2 | 2 | 1 | 15 |
| Li Yaqin (2008) | 2 | 1 | 1 | 1 | 1 | 2 | 1 | 2 | 2 | 1 | 14 |
| Qian Wencai (2008) | 2 | 1 | 1 | 2 | 1 | 1 | 1 | 2 | 2 | 1 | 14 |
| Li Youzi (2008) | 2 | 1 | 1 | 1 | 1 | 1 | 1 | 2 | 2 | 1 | 13 |
| Chen Zehua (2008) | 2 | 1 | 1 | 1 | 1 | 0 | 1 | 2 | 1 | 2 | 12 |
| Liu Baohua (2008) | 1 | 0 | 0 | 1 | 1 | 1 | 1 | 2 | 1 | 1 | 9 |
| Mu Yunzhen (2009) | 2 | 2 | 2 | 2 | 1 | 2 | 1 | 2 | 2 | 2 | 18 |
| Shang Yuxiu (2009) | 2 | 2 | 2 | 2 | 1 | 2 | 1 | 2 | 2 | 2 | 18 |
| Ye Rong (2009) | 2 | 1 | 2 | 2 | 2 | 2 | 2 | 1 | 2 | 2 | 18 |
| Cao Hongyuan (2009) | 2 | 1 | 2 | 2 | 1 | 2 | 1 | 2 | 2 | 2 | 17 |
| Zhou Xin (2009) | 2 | 1 | 1 | 1 | 2 | 2 | 1 | 2 | 2 | 1 | 15 |
| Li Wenwen (2009) | 2 | 2 | 0 | 1 | 1 | 2 | 1 | 2 | 2 | 2 | 15 |
| Yang Xiaohui (2009) | 2 | 2 | 1 | 1 | 1 | 1 | 1 | 2 | 2 | 1 | 14 |
| Jin Zhengguo (2009) | 1 | 1 | 1 | 1 | 0 | 1 | 1 | 2 | 2 | 1 | 11 |
| Zhao Shujuan (2009) | 2 | 1 | 1 | 1 | 0 | 0 | 1 | 2 | 1 | 1 | 10 |
| Liu Kerong (2009) | 1 | 1 | 1 | 1 | 1 | 0 | 1 | 2 | 1 | 1 | 10 |
| Zeng Zhuanping (2009) | 1 | 0 | 1 | 1 | 0 | 0 | 1 | 1 | 1 | 1 | 7 |
| Song Yumei (2010) | 2 | 2 | 2 | 2 | 1 | 1 | 1 | 2 | 2 | 2 | 17 |
| Fen Tianyi (2010) | 2 | 2 | 1 | 2 | 1 | 1 | 1 | 2 | 2 | 2 | 16 |
| Wang Fengsheng (2010) | 2 | 2 | 0 | 2 | 1 | 1 | 1 | 2 | 2 | 2 | 15 |
| Shen Liqin (2010) | 2 | 2 | 1 | 2 | 0 | 1 | 1 | 2 | 2 | 2 | 15 |
| Wang Jian (2010) | 2 | 1 | 1 | 1 | 2 | 1 | 1 | 2 | 2 | 2 | 15 |
| Yang Yanjie (2010) | 2 | 2 | 1 | 2 | 0 | 1 | 1 | 2 | 1 | 1 | 13 |
| Ge Xin (2010) | 2 | 1 | 1 | 1 | 1 | 1 | 1 | 2 | 1 | 1 | 12 |
| Ling Sun (2011) | 2 | 2 | 2 | 1 | 2 | 2 | 2 | 2 | 2 | 2 | 19 |
| Dong Guanbo (2011) | 2 | 2 | 2 | 2 | 1 | 2 | 1 | 2 | 2 | 2 | 18 |
| Ruan Ye (2011) | 2 | 2 | 2 | 2 | 2 | 0 | 2 | 2 | 2 | 2 | 18 |
| Zhu Shuang (2011) | 2 | 2 | 1 | 2 | 1 | 1 | 1 | 2 | 2 | 2 | 16 |
| Jiang Qing (2011) | 2 | 1 | 1 | 2 | 1 | 1 | 1 | 2 | 2 | 2 | 15 |
| Pan Xin (2011) | 2 | 2 | 0 | 2 | 1 | 1 | 1 | 2 | 2 | 1 | 14 |
| Wei Yali (2011) | 2 | 1 | 1 | 2 | 1 | 1 | 1 | 2 | 2 | 1 | 14 |
| Gao Shuhui (2011) | 2 | 2 | 0 | 2 | 1 | 0 | 1 | 2 | 2 | 2 | 14 |
| Zhang Guifeng (2011) | 2 | 1 | 1 | 1 | 1 | 0 | 1 | 2 | 2 | 1 | 12 |
| Zhao Qiuzhen (2011) | 2 | 1 | 1 | 1 | 1 | 1 | 1 | 2 | 1 | 1 | 12 |
| Xu Limei (2011) | 2 | 1 | 1 | 1 | 0 | 0 | 1 | 2 | 1 | 1 | 10 |
| Tan Erli (2011) | 1 | 1 | 0 | 0 | 1 | 1 | 1 | 2 | 1 | 1 | 9 |
| Wang Na (2012) | 2 | 2 | 2 | 1 | 1 | 2 | 1 | 2 | 2 | 2 | 17 |
| Wan Yuhui (2012) | 2 | 2 | 2 | 2 | 0 | 2 | 1 | 2 | 2 | 2 | 17 |
| Li Wei (2012) | 2 | 1 | 1 | 2 | 1 | 2 | 1 | 2 | 2 | 2 | 16 |
| Yang Chuanxiong (2012) | 2 | 2 | 1 | 2 | 1 | 1 | 1 | 2 | 2 | 2 | 16 |
| Fan,A.P. (2012) | 2 | 2 | 1 | 1 | 2 | 2 | 2 | 2 | 1 | 1 | 16 |
| Yang Yanfang (2012) | 2 | 2 | 1 | 2 | 1 | 2 | 1 | 2 | 1 | 1 | 15 |
| Shi Shenchao (2012) | 2 | 2 | 1 | 2 | 1 | 1 | 1 | 2 | 2 | 1 | 15 |
| Ding Jianfei (2012) | 2 | 1 | 1 | 2 | 1 | 1 | 1 | 2 | 2 | 1 | 14 |
| Liu Xiuhua (2012) | 2 | 2 | 1 | 1 | 1 | 1 | 1 | 2 | 2 | 1 | 14 |
| Wu Ling (2013) | 2 | 2 | 0 | 2 | 2 | 2 | 1 | 2 | 2 | 2 | 17 |
| Wang Dongping (2013) | 2 | 2 | 1 | 1 | 2 | 2 | 1 | 2 | 2 | 2 | 17 |
| Wang Jun (2013) | 2 | 2 | 1 | 2 | 1 | 1 | 1 | 2 | 2 | 2 | 16 |
| Liu Chang (2013) | 2 | 1 | 1 | 1 | 1 | 1 | 1 | 2 | 2 | 2 | 14 |
| Zhang Yuan (2013) | 2 | 1 | 0 | 2 | 0 | 1 | 1 | 2 | 2 | 2 | 13 |
| Ren Xiaohui (2013) | 2 | 1 | 0 | 2 | 0 | 0 | 1 | 2 | 2 | 2 | 12 |
| Yang Linsheng (2014) | 2 | 2 | 2 | 2 | 2 | 2 | 1 | 2 | 2 | 2 | 19 |
| Fan Yang (2014) | 2 | 2 | 2 | 2 | 1 | 2 | 2 | 2 | 2 | 2 | 19 |
| Yao Ran (2014) | 2 | 1 | 2 | 2 | 2 | 2 | 1 | 2 | 2 | 2 | 18 |
| Kunmi Sobowale (2014) | 2 | 1 | 1 | 2 | 2 | 2 | 2 | 2 | 2 | 2 | 18 |
| Aiming Zheng (2014) | 2 | 2 | 2 | 2 | 0 | 2 | 2 | 2 | 2 | 2 | 18 |
| Yang Linsheng (2014) | 2 | 1 | 2 | 2 | 1 | 2 | 1 | 2 | 2 | 2 | 17 |
| Qu Wei (2014) | 2 | 1 | 2 | 2 | 1 | 2 | 1 | 2 | 2 | 2 | 17 |
| Liu Yan (2014) | 2 | 2 | 1 | 2 | 2 | 1 | 1 | 2 | 2 | 2 | 17 |
| Tao Shuman (2014) | 2 | 2 | 2 | 2 | 1 | 1 | 1 | 2 | 2 | 2 | 17 |
| Xiang Pengcheng (2014) | 2 | 2 | 1 | 1 | 1 | 2 | 1 | 2 | 2 | 2 | 16 |
| Chen Fuxun (2014) | 2 | 1 | 1 | 1 | 1 | 2 | 1 | 2 | 2 | 2 | 15 |
| Wang Feiran (2014) | 2 | 1 | 1 | 1 | 1 | 2 | 1 | 2 | 2 | 1 | 14 |
| Liu Mei (2014) | 2 | 1 | 1 | 1 | 1 | 1 | 1 | 2 | 1 | 1 | 12 |
| Guo Kai (2014) | 2 | 1 | 1 | 1 | 0 | 0 | 1 | 2 | 2 | 1 | 11 |
| Meng Shi (2015) | 2 | 2 | 2 | 2 | 2 | 2 | 2 | 2 | 2 | 2 | 20 |
| Tian Yunqing (2015) | 2 | 2 | 2 | 2 | 2 | 2 | 1 | 2 | 2 | 2 | 19 |
| Liu Yan (2015) | 2 | 1 | 2 | 2 | 1 | 2 | 1 | 2 | 2 | 2 | 17 |
| Chang Hong (2015) | 2 | 2 | 1 | 2 | 2 | 2 | 1 | 2 | 2 | 1 | 17 |
| C.-J. CHEN (2015) | 2 | 1 | 1 | 1 | 2 | 2 | 2 | 2 | 2 | 2 | 17 |
| Zhang Kaili (2015) | 2 | 1 | 1 | 2 | 1 | 2 | 1 | 2 | 2 | 2 | 16 |
| Li Qiang (2015) | 2 | 1 | 0 | 2 | 1 | 2 | 1 | 2 | 2 | 2 | 15 |
| Guan Suzhen (2015) | 2 | 2 | 2 | 2 | 0 | 1 | 1 | 2 | 1 | 2 | 15 |
| Meng Shi (2015) | 2 | 2 | 1 | 1 | 1 | 2 | 2 | 1 | 1 | 2 | 15 |
| Yu Jiegen (2015) | 2 | 2 | 1 | 1 | 1 | 1 | 1 | 2 | 1 | 2 | 14 |
| Zhao Chuan (2015) | 2 | 2 | 1 | 2 | 1 | 0 | 1 | 2 | 2 | 1 | 14 |
| Yu Linlu (2015) | 2 | 1 | 1 | 1 | 1 | 1 | 1 | 2 | 2 | 1 | 13 |
| Hang Yazhu (2015) | 2 | 1 | 0 | 1 | 0 | 1 | 1 | 2 | 2 | 1 | 11 |
| Meng Shi (2016) | 2 | 2 | 2 | 2 | 2 | 2 | 2 | 2 | 2 | 2 | 20 |
| Gao Jie (2016) | 2 | 1 | 2 | 2 | 1 | 2 | 1 | 2 | 2 | 2 | 17 |
| Jiang Hongcheng (2016) | 2 | 2 | 2 | 2 | 1 | 1 | 1 | 2 | 2 | 2 | 17 |
| Dai Chengshu (2016) | 2 | 2 | 1 | 2 | 1 | 2 | 1 | 2 | 2 | 2 | 17 |
| Sun Weiwei (2016) | 2 | 2 | 1 | 2 | 2 | 2 | 1 | 2 | 2 | 1 | 17 |
| Huang Yalian (2016) | 2 | 2 | 2 | 1 | 1 | 1 | 1 | 2 | 2 | 2 | 16 |
| Qian Yunke (2016) | 2 | 1 | 0 | 2 | 1 | 1 | 1 | 2 | 2 | 2 | 14 |
| Lv Shixin (2016) | 2 | 2 | 0 | 2 | 2 | 1 | 1 | 2 | 1 | 1 | 14 |
| Qiu Nan (2016) | 2 | 1 | 1 | 1 | 1 | 1 | 1 | 2 | 1 | 1 | 12 |
| Wu Yingping (2016) | 2 | 1 | 0 | 1 | 1 | 0 | 1 | 2 | 1 | 1 | 10 |
| Long Sun (2017) | 2 | 2 | 2 | 2 | 2 | 2 | 2 | 2 | 2 | 2 | 20 |
| Fen Fenglian (2017) | 2 | 2 | 1 | 2 | 2 | 2 | 1 | 2 | 2 | 2 | 18 |
| Ma Xuan (2017) | 2 | 2 | 2 | 2 | 1 | 1 | 1 | 2 | 2 | 2 | 17 |
| Li Xiang (2017) | 2 | 2 | 2 | 2 | 1 | 1 | 1 | 2 | 2 | 2 | 17 |
| Li Xue (2017) | 2 | 2 | 1 | 2 | 2 | 1 | 1 | 2 | 2 | 2 | 17 |
| Chen Huan (2017) | 2 | 1 | 2 | 2 | 1 | 1 | 1 | 2 | 2 | 1 | 15 |
| Liang Peiyu (2017) | 2 | 2 | 2 | 2 | 1 | 1 | 1 | 2 | 1 | 1 | 15 |
| Xu Tao (2017) | 2 | 1 | 1 | 1 | 1 | 1 | 1 | 2 | 2 | 2 | 14 |
| Dai Ruoyi (2017) | 1 | 1 | 2 | 1 | 1 | 1 | 1 | 1 | 2 | 1 | 12 |
| Zeng Baoer (2018) | 2 | 2 | 2 | 2 | 2 | 2 | 2 | 2 | 2 | 2 | 20 |
| Ching-Yen Chen (2018) | 2 | 2 | 2 | 2 | 2 | 2 | 2 | 2 | 2 | 2 | 20 |
| Zhao Fei (2018) | 2 | 2 | 2 | 2 | 2 | 2 | 1 | 2 | 2 | 2 | 19 |
| Dan Wu (2018) | 2 | 2 | 2 | 2 | 1 | 2 | 2 | 2 | 2 | 2 | 19 |
| Lin Fen (2018) | 2 | 2 | 2 | 2 | 2 | 1 | 1 | 1 | 2 | 2 | 17 |
| Shi Junfang (2018) | 2 | 1 | 2 | 2 | 1 | 2 | 1 | 2 | 2 | 1 | 16 |
| Li Xiaoping (2018) | 2 | 2 | 0 | 2 | 1 | 1 | 1 | 2 | 2 | 2 | 15 |
| Jiang Nan (2018) | 2 | 1 | 1 | 1 | 2 | 2 | 1 | 2 | 2 | 1 | 15 |
| Li Xuanxuan (2018) | 2 | 2 | 1 | 2 | 1 | 1 | 1 | 2 | 2 | 1 | 15 |
| Sibo Zhao (2018) | 2 | 1 | 1 | 2 | 2 | 1 | 1 | 2 | 2 | 1 | 15 |
| Fen Fenglian (2018) | 2 | 1 | 1 | 1 | 1 | 1 | 1 | 2 | 2 | 2 | 14 |
| Wu Jinting (2018) | 2 | 1 | 1 | 2 | 0 | 1 | 1 | 2 | 1 | 2 | 13 |
| Zheng Chuanjuan (2018) | 1 | 2 | 1 | 1 | 0 | 1 | 1 | 2 | 2 | 1 | 12 |
| Jessica A Gold (2019) | 2 | 2 | 2 | 2 | 2 | 2 | 2 | 2 | 2 | 2 | 20 |
| Chunli Liu (2019) | 2 | 2 | 2 | 2 | 2 | 2 | 2 | 2 | 2 | 2 | 20 |
| Ling Wang (2019) | 2 | 2 | 2 | 2 | 2 | 2 | 2 | 2 | 2 | 2 | 20 |
| Xiaogang Zhong (2019) | 2 | 2 | 2 | 1 | 2 | 2 | 2 | 2 | 2 | 2 | 19 |
| Yanli Zeng (2019) | 2 | 2 | 2 | 2 | 2 | 2 | 1 | 2 | 2 | 2 | 19 |
| Zhao Xiujuan (2019) | 2 | 1 | 2 | 2 | 2 | 2 | 1 | 2 | 2 | 2 | 18 |
| Xiong Lin (2019) | 2 | 2 | 1 | 2 | 2 | 2 | 1 | 2 | 2 | 2 | 18 |
| Liu Jing (2019) | 2 | 1 | 2 | 2 | 1 | 2 | 1 | 2 | 2 | 2 | 17 |
| Tang Siyao (2019) | 2 | 2 | 1 | 2 | 2 | 1 | 1 | 2 | 2 | 2 | 17 |
| Cao Lei (2019) | 2 | 1 | 0 | 1 | 2 | 2 | 1 | 2 | 2 | 2 | 15 |
| Wang Zhe (2019) | 1 | 1 | 0 | 2 | 2 | 2 | 1 | 2 | 2 | 1 | 14 |
| Steven W. H. Chau (2019) | 2 | 2 | 1 | 2 | 0 | 1 | 1 | 2 | 1 | 1 | 13 |
| Lin Xin (2019) | 2 | 1 | 1 | 1 | 1 | 1 | 1 | 2 | 1 | 1 | 12 |
| Li Zhongcheng (2019) | 2 | 1 | 1 | 1 | 1 | 1 | 1 | 2 | 1 | 1 | 12 |
| Ai Dong (2019) | 1 | 1 | 1 | 2 | 1 | 0 | 1 | 2 | 1 | 1 | 11 |
| Wanjie Tang (2020) | 2 | 2 | 2 | 2 | 2 | 2 | 2 | 2 | 2 | 2 | 20 |
| Yanmei Shen (2020) | 2 | 2 | 2 | 2 | 2 | 2 | 2 | 2 | 2 | 2 | 20 |
| Jing Guo (2020) | 2 | 2 | 2 | 2 | 2 | 2 | 2 | 2 | 2 | 2 | 20 |
| Ruyue Shao (2020) | 2 | 2 | 2 | 2 | 2 | 2 | 1 | 2 | 2 | 2 | 19 |
| Chen Jun (2020) | 2 | 2 | 1 | 2 | 1 | 2 | 1 | 2 | 2 | 2 | 17 |
| Yang Xueling (2020) | 2 | 2 | 2 | 1 | 1 | 2 | 1 | 2 | 2 | 2 | 17 |
| Liu Ningning (2020) | 2 | 2 | 1 | 1 | 2 | 1 | 1 | 2 | 2 | 1 | 15 |
| Zhu Huiquan (2020) | 2 | 2 | 1 | 1 | 1 | 1 | 1 | 2 | 2 | 2 | 15 |
| Liu Xia (2020) | 2 | 2 | 1 | 1 | 0 | 1 | 1 | 2 | 2 | 2 | 14 |
| Xu Limei (2002) | 2 | 1 | 0 | 2 | 1 | 2 | 1 | 1 | 2 | 2 | 14 |
| Meng Zhaoying (2007) | 2 | 2 | 0 | 2 | 1 | 2 | 1 | 2 | 2 | 2 | 16 |
| Liao Yanhui (2010) | 2 | 2 | 1 | 2 | 2 | 1 | 1 | 2 | 2 | 2 | 17 |
| Xin Shen (2010) | 2 | 2 | 0 | 2 | 2 | 2 | 1 | 2 | 2 | 2 | 17 |
| Liu Rui (2014) | 2 | 2 | 0 | 2 | 1 | 1 | 1 | 2 | 2 | 2 | 15 |
| Pan X.F. (2016) | 2 | 2 | 2 | 2 | 1 | 1 | 2 | 2 | 2 | 2 | 18 |

**S_1.2 The items of JBI Systematic Reviews Checklist for Prevalence Studies (JBI checklist)**

| **Items** | *Y* | *N* |
| --- | --- | --- |
| Item 1. Was the purpose of the study clear and the basis of the topic sufficient? |  |  |
| Item 2. Were the study subjects and the setting described in detail? |  |  |
| Item 3. Were inclusion and exclusion criteria for study subjects clearly described? |  |  |
| Item 4. Was the sample frame appropriate to address the target population? |  |  |
| Item 5. Were valid methods used for the identification of the condition? |  |  |
| Item 6. Were appropriate measures taken to ensure the authenticity of the information? |  |  |
| Item 7. Were ethical issues considered? |  |  |
| Item 8. Was there appropriate statistical analysis? |  |  |
| Item 9. Was the presentation and analysis of the results appropriate and accurate? |  |  |
| Item 10. Was the value of the research clearly stated? |  |  |

*Note:* ***Y****=Yes;* ***N****=No*

**S_2. Included studies of this study**

1. Rong Y, Luscombe GM, Davenport TA, Huang Y, Glozier N, Hickie IB. Recognition and treatment of depression: a comparison of Australian and Chinese medical students. Social psychiatry and psychiatric epidemiology 2009; 44(8): 636-42.

2. Liao Y, Knoesen NP, Deng Y, et al. Body dysmorphic disorder, social anxiety and depressive symptoms in Chinese medical students. Social psychiatry and psychiatric epidemiology 2010; 45(10): 963-71.

3. Sun L, Sun LN, Sun YH, et al. Correlations between psychological symptoms and social relationships among medical undergraduates in Anhui Province of China. International journal of psychiatry in medicine 2011; 42(1): 29-47.

4. Fan AP, Kosik RO, Mandell GA, et al. Suicidal ideation in medical students: who is at risk? Annals of the Academy of Medicine, Singapore 2012; 41(9): 377-82.

5. Sobowale K, Zhou N, Fan J, Liu N, Sherer R. Depression and suicidal ideation in medical students in China: a call for wellness curricula. International journal of medical education 2014; 5: 31-6.

6. Yang F, Meng H, Chen H, et al. Influencing factors of mental health of medical students in China. Journal of Huazhong University of Science and Technology Medical sciences 2014; 34(3): 443-9.

7. Zheng A, Wang Z. Social and psychological factors of the suicidal tendencies of Chinese medical students. BioPsychoSocial medicine 2014; 8: 23.

8. Chen CJ, Chen YC, Sung HC, Hsieh TC, Lee MS, Chang CY. The prevalence and related factors of depressive symptoms among junior college nursing students: A cross‐sectional study. Journal of Psychiatric and Mental Health Nursing 2015; 22(8): 590-8.

9. Shi M, Liu L, Wang ZY, Wang L. The mediating role of resilience in the relationship between big five personality and anxiety among Chinese medical students: a cross-sectional study. PloS one 2015; 10(3): e0119916.

10. Shi M, Liu L, Yang Y-L, Wang L. The mediating role of self-esteem in the relationship between big five personality traits and depressive symptoms among Chinese undergraduate medical students. Personality and Individual Differences 2015; 83: 55-9.

11. Pan XF, Wen Y, Zhao Y, et al. Prevalence of depressive symptoms and its correlates among medical students in China: a national survey in 33 universities. Psychology, health & medicine 2016; 21(7): 882-9.

12. Shi M, Liu L, Wang ZY, Wang L. Prevalence of depressive symptoms and its correlations with positive psychological variables among Chinese medical students: an exploratory cross-sectional study. BMC psychiatry 2016; 16: 3.

13. Sun L, Zhou C, Xu L, Li S, Kong F, Chu J. Suicidal ideation, plans and attempts among medical college students in china: The effect of their parental characteristics. Psychiatry research 2017; 247: 139-43.

14. Chen CY, Yu NW, Huang TH, Wang WS, Fang JT. Harm avoidance and depression, anxiety, insomnia, and migraine in fifth-year medical students in Taiwan. Neuropsychiatric disease and treatment 2018; 14: 1273-80.

15. Wu D, Yang T, Rockett IR, Yu L, Peng S, Jiang S. Uncertainty stress, social capital, and suicidal ideation among Chinese medical students: Findings from a 22-university survey. Journal of health psychology 2018: 1359105318805820.

16. Zeng B, Zhao J, Zou L, et al. Depressive symptoms, post-traumatic stress symptoms and suicide risk among graduate students: The mediating influence of emotional regulatory self-efficacy. Psychiatry research 2018; 264: 224-30.

17. Zhao S, Zhang J. The Association Between Depression, Suicidal Ideation and Psychological Strains in College Students: A Cross-National Study. Culture, medicine and psychiatry 2018; 42(4): 914-28.

18. Chau SWH, Lewis T, Ng R, et al. Wellbeing and mental health amongst medical students from Hong Kong. International review of psychiatry (Abingdon, England) 2019; 31(7-8): 626-9.

19. Gold JA, Hu X, Huang G, et al. Medical student depression and its correlates across three international medical schools. World journal of psychiatry 2019; 9(4): 65-77.

20. Liu C, Wang L, Qi R, et al. Prevalence and associated factors of depression and anxiety among doctoral students: the mediating effect of mentoring relationships on the association between research self-efficacy and depression/anxiety. Psychology research and behavior management 2019; 12: 195-208.

21. Wang L, Yang Y, Zhu J, et al. Professional identity and mental health of rural-oriented tuition-waived medical students in Anhui Province, China. BMC medical education 2019; 19(1): 199.

22. Zeng Y, Wang G, Xie C, Hu X, Reinhardt JD. Prevalence and correlates of depression, anxiety and symptoms of stress in vocational college nursing students from Sichuan, China: a cross-sectional study. Psychology, health & medicine 2019; 24(7): 798-811.

23. Zhong X, Liu Y, Pu J, et al. Depressive symptoms and quality of life among Chinese medical postgraduates: a national cross-sectional study. Psychology, health & medicine 2019; 24(8): 1015-27.

24. Guo J, Meng D, Ma X, Zhu L, Yang L, Mu L. The impact of bedtime procrastination on depression symptoms in Chinese medical students. Sleep & breathing = Schlaf & Atmung 2020; 24(3): 1247-55.

25. Shao R, He P, Ling B, et al. Prevalence of depression and anxiety and correlations between depression, anxiety, family functioning, social support and coping styles among Chinese medical students. BMC psychology 2020; 8(1): 38.

26. Shen Y, Zhang W, Chan BSM, et al. Detecting risk of suicide attempts among Chinese medical college students using a machine learning algorithm. Journal of affective disorders 2020; 273: 18-23.

27. Tang W, Kang Y, Xu J, Li T. Associations of Suicidality with Adverse Life Events, Psychological Distress and Somatic Complaints in a Chinese Medical Student Sample. Community mental health journal 2020; 56(4): 635-44.

28. DU ZY, ZHONG WF, ZU JS. A Cross-sectional Study on Depression Symptoms of Students in Jining Medical College. Chinese Journal of School Health 2000; 21(5): 345,7.

29. Lin DX, Wang WP, Wu RW, et al. Investigation and Analysis of the Psychological Quality of Medical Students in Higher Vocational Schools (in chinese) Journal of Fujian Medical University(Social Science Edition) 2000; (02): 65-6+10.

30. Wu HL, Ma CG, Wang LS, Wang FF, Fen YR. Analysis of Depression and its Influencing Factors of Medical Students in Colleges and Technical Secondary Schools (in Chinese). China Journal of Health Psychology 2000; (03): 279-82.

31. Yang BF, Zhang ZJ, Yue XT, Ji F. Study of sleep quality and anxiety depression in medical college students. Chinese Journal of Behavioral Medicine and Brain Science 2000; 9(5).

32. Huang J, Wang JJ, Su XM, Wang QX. Anxiety of Students in a Medical University (in Chinese). Chinese Journal of School Health 2001; 22(6): 555.

33. Lin ZP, Yu B. Investigation on Mental Health of Medical Students and Its Influencing Factors (in Chinese). Journal of Guiyang Medical College 2001; 26(5): 391-3.

34. Su XM, Liu JA, Jing J, et al. Anxiety and its Risk Factors of Girls in Nursing School (in Chinese). Maternal and Child Health Care of China 2001; (10): 647-9.

35. Yu M, Cao JP. Investigation on mental health of 509 medical students (in Chinese). Strait Journal of Preventive Medicine 2001; 7(4): 32-3.

36. Zhang YS. Research on the Coping Style toward Anxiety and Depression in a Medical College Student (in Chinese). Chinese Journal of School Health 2001; (02): 157.

37. Zhang YS, Du FG, Gao LZ, Wang GY. Survey on mental health of medical students in grade 2000 Journal of Henan University(Medical Science) 2001; 20(2).

38. Hu LR, Kong DL, Chen GJ, Tan QH, Xu Y. Descriptive Study on Health Risk Behaviors of Students in a Medical College. Chinese Journal of School Health 2002; (01): 20-1.

39. Qi YL, Zhai CP. Discussion on Anxiety and Its Influencing Factors of Freshmen in Bengbu Medical College (in Chinese) Chinese Journal of School Health 2002; 23(6): 534-.

40. Rao H, Shen X, Wang Q, Chen W. Depression in military medical students and influential factors. Chinese Mental Health Journal 2002; 16(12): 865-.

41. Xu LM, Guang WP, Wei XQ, Li WY, Sun X. Investigation and Analysis of Anxiety and Depression of Lower-grade Students in Medical Colleges (in Chinese). China Journal of Health Psychology 2002; 10(1): 24-5.

42. Ge SZR, Liu LK, Hang M, Su DT. A Survey on Depression and Scl －90 Results of Students in Nurse Technical School. Modern Preventive Medicine 2003; 30(6): 888-9.

43. Wang ML. Study on the Relationship between Personality Trait and Depression among Medical Students. Chinese Journal of School Health 2003; 24(5).

44. Zhang XW, Ren Y. Investigation and Analysis of Anxiety of Medical Students (in Chinese) Acta Neuropharmacologica 2003; 20(4): 21-3.

45. Zheng WJ, Tang ZH. Analysis of state of psychology medical students when facing SARS (in Chinese). Chinese Mental Health Journal 2003; (09): 599.

46. Zhou R, Yang CH, Pan JY. Investigation and analysis of depression and its related factors of key medical college students in Guangzhou (in Chinese). Journal of Neuroscience and Mental Health 2003; 3(5): 367-8.

47. Fu - Quan Z, Qiu - Ping T, Yun-Long D. An Analysis of Mental Health Status of Medical Students and Its Related Factors. Chinese Journal of Clinical Psychology 2004; 12(1): 63-5.

48. Liang DH, Wang FZ, Yu GH, et al. An investigation on risk behavior of injury among medical students. Chinese Journal of Disease Control & Prevention 2004; (06): 525-7.

49. Zhang SY. Investigation on the Mental Health of Medical Students and Its Related Factors (in Chinese). Journal of Tongji University（Medical Science） 2004; 25(2).

50. Fen FL, Liang L, Yang JX. Investigation on life events of medical university students and their relationship with anxiety and depression (in Chinese). Journal of Hebei Medical University 2005; 26(2): 139-40.

51. Ge SZR, Liu LK, Zhang Q, Hang M, Zhu CH, Su DT. A Study on Epidemiological Characters of 1,100 Medical Student’s Depression Situation and the Relationship Between Depression Situation and Self-acceptance and Tropism. Modern Preventive Medicine 2005; 32(1): 16-9,26.

52. Guo R, Yu YS. Study of Depression and Theirs Influencing Factors by Logistic Regression Analysis in Medical College Students. Journal of Qiannan Medical College for Nationalities 2005; 18(1): 37-9.

53. Hu LR, Ding YL. A study on incidence and related risk behaviors of suicide ideation among medical students. Chinese Journal of Disease Control & Prevention 2005; 9(1): 18-20.

54. Li YC, Hu CL, Tao XY, Ruan L, Jing SB. Study on the Relationship of Medical Student's anxiety, Depression and Individuality. Chinese Journal of School Health 2005; 26(2): 107-8.

55. Ren HN, Yang XQ, Zhang JS, Liu MY, Gong HH, Liu SB. Correlation between Anxiety, Depression and Personality Characteristics of Medical Students (in Chinese). Chinese Journal of School Health 2005; 26(11): 953-,5.

56. Wang DQ, Wang BG, Zhou WP, Chen SD, Xiao J. The risk factors of suicide ideation in the college of medicine and pharmacy. Chinese Journal of Disease Control & Prevention 2005; 9(1): 21-2.

57. Wei XQ, Guang WP, Li WY, Ji YL, Xu LM. Analysis of psychological test of anxiety, depression and psychiatric symptoms in medical college students. Journal of China Medical University 2005; 34(1): 47-8.

58. Xiaoning S, Xiaohong L, Yan X, Chuanyun L. A Path Analysis of Influencing Factors of Psychological Stress in Military Medical Students. Psychological Science (China) 2005; 28(1): 76-9.

59. Xu LM. Analysis and Countermeasures of psychological emotion of medical graduates. China Journal of Health Psychology 2005; 13(4).

60. Yang XZ, Chen JW, Fen GS. The Analysis to the Correlation among Sleep Quality and Anxiety and Depression in Medical College Students. Acta Academiae Medicinae Weifang 2005; 27(2): 100-2.

61. Jin J, Zhai DC, Pan XD, Zhou XS. Investigation on the status of depression and anxiety among medical college students. Chinese Journal of School Doctor 2006; 20(6): 599-600.

62. Mei L, Zhang FY, Liang Y, Zhang BY, Wang XQ, Tan HR. Analysis of the mental health status between medical and non- medical students. China Medical Equipment 2006; 3(3): 46-8.

63. Song J, Yu YZ, Mao BQ, Wang L, Hu SR. Investigation and Analysis on Mental Health State of Clinical Students. Chinese Journal of Social Medicine 2006; 0(3).

64. Wang XL, Xu NF, Xue HD, Jiang DW, Yang DH. Analysis on suicide ideation and its risk factors among medical undergraduate students. Chinese Journal of Public Health 2006; 22(6): 653-5.

65. Wang YF, Yang CH, Pan JY. The Anxiety States of the Medical Students in One Key Medical College in Guangzhou City. China Journal of Health Psychology 2006; 14(2): 220-1.

66. Wei JB. Study on anxiety and depression and their relation in medical students. Journal of Xinxiang Medical University 2006; 23(6): 550-2.

67. Wu Y, Wang LG. Correlation Study between Life Events and Family and Depressive symptoms of Medical Students. Medicine and Society 2006; 19(4): 41-3.

68. Xu ML, Zhang HB, Xu J, Xu ZJ, Su JY. Socio-demographic and Social Psychological Analysis of College Students with Anxiety in Anhui Province. Chinese Mental Health Journal 2006; 20(8).

69. Zeng Q, Li L, Tang SY, et al. Analysis on Influential Factor and Preventive Way of Depression Among College Students in Certain University. Practical Preventive Medicine 2006; (06): 1412-3.

70. Zhai DC, Pan XD, Han SL, Mou J, Zhou XS. The Epidemic Features and Related Factors of Depression and Anxiety Among Medical Students. Chinese Journal of School Health 2006; 27(3): 217-9.

71. Zhang ZW, Wu CP, Liu GN, Fan CX, Wang HS. Analysis on depression and its influence factors of 488 medical students. Chinese Journal of Public Health 2006; 22(6): 649-50.

72. Zhang ZW, Wu CP, Liu GN, Fan CX, Wang HS. Depression and the Influencing Factors of Medical College Students in Guangzhou. Chinese Journal of School Health 2006; 27(1): 51-2.

73. Deng SG, Wu QJ, Guo R. Investigation of the Correlation Between Psychological Health Status and Life Events of Medical Students in the Minority Areas. Modern Preventive Medicine 2007; 34(15).

74. Hu YR, Tan QH, Wang XJ, Ding YL. Study on interrelationship of suicide attempts and sex-related behaviors among medical students. Chinese Journal of Public Health 2007; 23(5): 541-3.

75. Li L, Wang J, Su YY. Mental Health Diagnoses of Freshmen in Liaoning Medical University. Journal of Jinzhou Medical University(Social Science Edition) 2007; 5(4).

76. Liang XR, Long SK, Wang XL. Study on the Status of Anxiety of Medical College Students of Grade One in Junior College in Guangxi. Modern Preventive Medicine 2007; 34(14): 2701-3.

77. Liu YL, Zhou WJ, Zhang W. Investigation and analysis the mental status of clinical and nusing undergraduate students. Chinese Journal of Modern Nursing 2007; 13(31): 3038-9.

78. Meng ZY. Investigation on the Relationship between Sl eep Quality and Anxious Depressive Mood of Medical Students. China Journal of Health Psychology 2007; 15(10): 868-70.

79. Sang WH, Li KQ, Qin XH, Wang YF. Depression Disorder and Life Events, Personality of Medical Students in Health School. China Journal of Health Psychology 2007; 15(11): 1013-5.

80. Wang T, Wang NW, Hu HQ, Fen ZZ, Liu YB. Relationship of depression automatic thoughts and personality in medical college students. Journal of Third Military Medical University 2007; 29(5): 442-4.

81. Zhang XY, Yu SY, Zhao JB. Correlation between suicidal ideation and personality internal external locus of control self-estem in medical college students. Negative 2007; 28(8): 747-9.

82. Chen ZH, Zhong HB. The prevalence of injury-related behaviors of medical students and interventive measurements (in Chinese). Practical Preventive Medicine 2008; 15(2).

83. Du J, Li Y, Niu J, Zhang H-j. Correlative study on mental health, coping styles and posttraumatic stress response among medical students. Chinese Journal of Clinical Psychology 2008; 16(2): 183-5.

84. Fan YG, Xiao Q, Li WX, Song YM, Ye QL, Ye DQ. Correlated Study Between Social Psychological Factors and Suicide Ideation Among Medical Public Health School of Anhui Medical University. Chinese Journal of School Health 2008; 29(4): 328-30.

85. Hu ZH. Study on the related factors of suicidal ideation in college students of military medical university. Chinese Journal of Medical Education Research 2008; 7(2): 222-4.

86. Li YQ, Liu WM, Sun JH. Investigation and analysis about depression in medical college students. Journal of Modern Medicine 2008; 18(18): 2728-30,35.

87. Li YZ, Gu LL, Wang Y, Du Y, Liang DH. An investigation of mental health status and suicide ideation in medical undergraduates. Journal of Shenyang Medical College 2008; 10(4).

88. Liu BH, Huang YQ, Niu WY, Lv ZZ, Yue C, Wang PY. Study on the factors influencing suicidal ideation among medical students in Beijing. Zhonghua Liu Xing Bing Xue Za Zhi 2008; 29(2): 128-31.

89. Ou GZ, Wu ZD, Xu Y. Analysis on suicide ideation and related factors among medical junior class students in Fujian Province. Chinese Journal of Health Education 2008; 24(3): 167-9.

90. Qian WC, Liu BH, Huang YQ, Hao WL, Li W, Wang PY. Study on risk factors for suicidal ideation among undergraduates of medical college, China. Chinese Journal of Public Health 2008; 24(11).

91. Wang X, Zhao JF, Li MQ, Xiao LL, Guo M. Study on the relationship between suicidal ideation and mental health status as well as personality characteristics of medical students in old revolutionary areas (in Chinese). Journal of Qiqihar Medical University 2008; 29(20): 2454-5.

92. Yang BF, Song HM, Liu XH, Cao FF, Hu QQ. A Study on Suicide Ideation and Its Influencing Factors among Medical Undergraduate Students. Chinese Journal of Social Medicine 2008; 25(2): 98-100.

93. Cao HY, Sun YH, Yao YS, Zhou CS, Yang LS. Suicide Behavior and Its Risk Factors Among Medical Undergraduates in Anhui Province. Chinese Journal of School Health 2009; 30(1): 38-9.

94. Jin ZG, Xiong YH, Fang JN. Relationship between psychological health status and life events among the students in College of Medicine. Journal of Medical Science Yanbian University 2009; (2).

95. Li WW, Li XC, Luo YJ, Liao MS, Lu WL, Wei X. Investigation on psychological distress of medical graduates in Guangzhou (in Chinese). Chinese Journal of General Practice 2009; 7(11): 1220-1.

96. Liu KR, Hu GF, Zhang MY, Yan YX, Nie J. Psychological anxiety evaluation and analysis of graduates at a medical university under employment pressure. Journal of Southern Medical University 2009; 29(5): 1071-2.

97. Mu YZ. Comparion of Mental Health Status between Han and Minority Medical Students. Journal of Kunming Medical University 2009; 30(8).

98. Shang YX, Yan SZ, Zhu FP. Study on the Current Status of Suicidal Ideation and Depression and Its Influence Factors Among Medical Students. Modern Preventive Medicine 2009; 36(16): 3092-4.

99. Yang XH, Jing LS, Yan H, et al. Analysis on the Associativity between Depressive Disorder and Suicide Ideation of Medical Students. China Journal of Health Psychology 2009; 17(1): 19-21.

100. Zeng ZP, Yang Y. Risk Factors for Suicide Attempt among Medical College students. Chinese Journal of Social Medicine 2009; 26(3): 158-60.

101. Zhao SJ, Wang WP, Zhang SY, Jiang F. Relationshiop Between Depression and Self-congruence Among Freshmen in Medical University. Chinese Journal of School Health 2009; 30(11): 1005-6.

102. Zhou X, Li JM. Study on Sleeping Quality of Nursing Students in College and Its Relationship with Anxiety and Depression. China Journal of Health Psychology 2009; 17(10): 1220-3.

103. Fen TY. The Investigation and Analysis on Medical Student Psychological Anxiety and Influencing Factors. Journal of Ningxia Medical University 2010; 32(7): 786-8.

104. Ge X, Wang Y. Study on Relationship between Anxiety and Life Events in Medical Secondary Vocational Students. China Journal of Health Psychology 2010; 18(07): 843-5.

105. Shen LQ, Hao QH, Zhang XL. Analysis of Suicide Idea and Analysis of Psychological Problems in Medical Undergraduate Students. Modern Preventive Medicine 2010; 37(6): 1089-90.

106. Song YM, Wan YH, Pan FM, Ye DQ. Analysis of and suicide ideation and its influencing factors of suicidal ideation among students in a medical college (in Chinese). Chinese Journal of Health Statistics 2010; 27(2): 178-9.

107. Wang FS, Sun YH, Niu JJ, Cai B, Gong L, Sun LN. A study on the relationship between family environment and anxiety of the undergraduates in medical universities. Chinese Journal of Epidemiology 2010; 31(4): 475-6.

108. Wang J, Sun FZ, Yan K. Investigation of Relationshop between Suicidal Tendency and Mental Stress in Medical students. Journal of Capital Medical University 2010; 31(03): 377-80.

109. Xin S, He CS. Analysis of related factors of suicidal ideation in medical students (in Chinese). Anhui Medical Journal 2010; 31(5): 521-3.

110. Yang YJ, Qiu XH, Yang XX, et al. Study on the influencing factors of suicidal ideation among university students in Harbin. Zhonghua Liu Xing Bing Xue Za Zhi 2010; 31(10): 1103-6.

111. Dong GB, Liu XW, Li PF, Wang X. Study on the depression psychological state of eight-year program students majoring basic medical science at postgraduate stage. Chinese Journal of Medical Education Research 2011; 31(2): 209-12.

112. Gao SH, Jia XY. Research on the relationship between depression and personality characteristics of medical students (in Chinese). Nursing Practice and Research 2011; 08(11): 13-4.

113. Jiang Q, Lv QQ. Investigation on Anxiety, Depression and Related Psychological Susceptibility Factors of Clinical Graduates (in Chinese). Journal of Fujian Medical University(Social Science Edition) 2011; 12(01): 33-6.

114. Pan X, Shi HF, Kang Y. Study on Influential Factors of Anxiety of Medical University Students from Han and Minority. China Journal of Health Psychology 2011; 19(12): 1503-5.

115. Ruan Y, Wen SL, Cao YQ. Analysis on Anxiety Risk Factors of Medical College Students in Lanzhou. Chinese Primary Health Care 2011; 25(9): 73-5.

116. Tan EL. Multiple regression analysis of depression and its influencing factors in medical college students (in Chinese). Journal of Qiqihar Medical University 2011; 32(21).

117. Wei YL. Survey of depression in medical college freshmen. Journal of Qiannan Medical College for Nationalities 2011; (1).

118. Xu LM, Qiao X. Comparison and Analysis about Anxiety and Depression of Medical Students during Entrance and Graduation Stage. Chinese Medical Record 2011; 12(5): 56-7.

119. Zhang GF, Ma H, Huang Y, Liu YY, Su PZ. Correlation between Internet addiction disorder and depression in students of a medical university Chinese Journal of School Doctor 2011; 25(10): 739-41.

120. Zhao QZ. The Influence of perceived social support of Medical students on anxiety and depression (in Chinese). Journal of Hebei North University(Natural Science Edition) 2011; 27(3).

121. ZHU S, FAN YY, LIU KW, Meng XW, Sun CY, Cao JQ. Associations of Anxiety with General Self － Efficacy and Coping Style among Medical Students. Chinese General Practice 2011; 14(22): 2572-3.

122. Ding JF, Zhang HB, Wang J, She M, Duan LW. Analysis on the depressive symptoms and its cognitive characteristic among college students in a medical university. Chinese Journal of Disease Control & Prevention 2012; 16(6): 499-501.

123. Li W. Research into the mental health status of the seven-year program medical students and its related influencing factors. Journal of Chongqing Medical University 2012; 37(3).

124. Liu XH, Li X, Yuan J. Correlation analysis of medical students' depression and its influencing factors in medical students (in Chinese). Chinese Journal of Aesthetic Medicine 2012; 21(18): 205-6.

125. Shi SC, Yang CW, Jia SS, Zhang JS, Li YF. Study on current status of depression and its influencing factors among 1 372 medical college students Chinese Journal of School Doctor 2012; 26(10): 727-9.

126. Wan Y-h, Gao R, Tao X-y, Tao F-b, Hu C-l. Relationship between deliberate self-harm and suicidal behaviors in college students. Zhonghua liu xing bing xue za zhi = Zhonghua liuxingbingxue zazhi 2012; 33(5): 474-7.

127. Wang N, Xie FX, Tan FF, Zhao Y. Research of Depression and Psychological Health Status in Medical College Student. Practical Preventive Medicine 2012; 19(3): 451-3.

128. Yang CX, Shi SC, Jia SS, Chui WW, Zhang JS, Li YF. Correlation study of anxiety，depression and suicide idea among medical college students. Journal of Xinxiang Medical University 2012; 29(11): 840-2.

129. Yang YF, Wu D, Guang MJ, Wang WP. Analysis of depression status of 331 medical students (in Chinese). Journal of Baotou Medical College 2012; 28(5): 29-30.

130. Liu C, Zhao Q. Relationship between Suicide Idea and Loneliness in Medical Students. China Journal of Health Psychology 2013; 21(11): 1747-9.

131. Ren XH. Depression epidemiological studies of college students Journal of Harbin Medical University 2013; 47(5).

132. Wang DP, Zhan HQ, Wei SQ. The Effect of Psychological Health Education Activities on Easing the Anxiety and Depression of Medical Students. China Journal of Health Psychology 2013; 21(12): 1852-3.

133. Wang J, Jin YL, Chen Y, Yu JG, He LP, Yao YS. Correlation analysis between depressed mood and life events among medical college students. Journal Of Wannan Medical College 2013; 32(2): 151-3.

134. Wu L, Wang XD, Tian Z. The Cross-sectional Survey of Suicidal Intention in Medical Students in Hainan. China Journal of Health Psychology 2013; 21(10): 1587-8.

135. Zhang Y, Liu QQ. Research on Suicide Attempts and Suicide Attitude Status of Medical University Undergraduates-to Take Kunming Medical University as an Example. Journal of Kunming Medical University 2013; (12): 30-2,6.

136. Chen FX, Li R, Xu CY, Yu XL, Liu YZ, Li QW. Status Survey and Verification Analysis on Anxiety among a Certain Medical University Students in Shandong. Medicine and Society 2014; (7): 66-8.

137. Guo K, Liu ML, Chen W, Wang ZF. Investigation and Research on Depression and Its Influencing Factors of Medical Students in Qinghai University (in Chinese). Chinese Journal of Disease Control & Prevention 2014; 18(5): 475-6.

138. Linsheng Y, Zhihua Z, Liang S, Hongyan W, Yehuan S. Risk and risk factors of suicide attempt after first onset of suicide ideation: findings from medical students in grades 1 and 2. Journal of Hygiene Research 2014; 43(1): 47-53.

139. Liu M, Wu HS. Status and relationship of depression symptoms, neurotic personality traits, coping styles in medical college students (in Chinese). Journal of Chengdu University of Traditional Chinese Medicine(Educational Science Edition) 2014; 0(1): 67.

140. Liu R, Du RJ, Dai XY, Wang ZF. Relationship between depression status of 278 Uyghur college students and trace elements in hair. Practical Preventive Medicine 2014; 21(11): 1297-9+3.

141. Liu Y, Zhang Y, Shi XZ, Wang S, Liang DH. Effect of psychosocial factors on suicidal ideation in medical college students. Chinese Journal of Public Health 2014; 30(3): 269-72.

142. Qu W, Ding N, Pan FM, Song XY, Wu SS. Depression and anxiety status and related factors of medical college students in a vocational college of Anhui province. Anhui Medical and Pharmaceutical Journal 2014; 18(1): 71-4.

143. Tao SM, Wu XY, Liu YH, Zhang YY, Zhang SC, Tao FB. Self-harm in medical college students and its relation with mobile phone

dependence and depressive symptoms Chinese Mental Health Journal 2014; 28(6): 472-7.

144. Wang FR. Main effect factors of psychosomatic symptoms of medical students. Medical Research and Education 2014; 31(2).

145. Xiang PC, Yang YF, Wu D, Pang XL. Research on the Correlation of Depression Status quo of Han Nationality Medical Students with Their Life Events and Gene Polymorphism of Serotonin Transporter. Journal of Baotou Medical College 2014; (6): 35-7,40.

146. Yang LS. Association between the early history of suicide behaviors in childhood or early adolescent and suicidal behaviors in the last year among college students. Chinese Journal of School Health 2014; 35(2).

147. Yao R, Chen D, Liu ML, et al. Study on the Ｒelationship between Internet Addiction Disorder with Suicidal Ideation and Depression amomg a Certain Pharmaceutical University in Guangdong Province. Medicine and Society 2014; 27(8): 83-5.

148. CHANG Hong, WEN Ying, LI Si-qi, et al. Prevalence of depressive and anxiety symptoms among medical students, southwest China. Modern Preventive Medicine 2015; 42(19): 3544-7+50.

149. Chuan Z, Man-jie Q, Pan-pan W, Ning L, Li X. Status and risk of anxiety and depression among students in a medical university. Occupation and Health 2015; 31(12): 1691-3.

150. Guan SZ, Liu XY, Zheng P, Liu JW, Ge H. The influence of family factors on suicidal thoughts of medical students. Chinese Preventive Medicine 2015; 16(1): 36-8.

151. Hang YZ. Reason Analysis and Prevention of Depression among Students in a Medical College. Journal of Shenyang Medical College 2015; 17(1).

152. Kai-li Z, Xia W, Si-yuan T, et al. Meaning of Life of Medical Undergraduates and Its Influence Factors. Journal of Nursing(China) 2015; 22(15): 36-9.

153. Li Q. Investigation and analysis of undergraduate medical college students in Henan Province. Chongqing Medicine 2015; (16): 2237-8,41.

154. Lin-lu， Y, Xi， T, Yang， L, Ling-xi， C, Lu， C, Jun-hui K. Survey on depression emotion among students from medical universities /colleges. Guangxi Medical Journal 2015; 37(8): 1076-8.

155. Liu Y, Tan X, Li Y, Xu F, Zhang J, Kong JH. A survey on depression in college students and influencing factors. Chinese Journal of General Practice 2015; 13(01): 91-3.

156. Tian YQ, Yu LL, Chen LX, Chen L, Kong JH. The Situation and Countermeasures of Anxiety in Medical University Students. China Journal of Health Psychology 2015; (1): 153-5,6.

157. Yu JG, Lu W, Chang WW, et al. Depression status and its relationship with self acceptance among female medical university student. Journal of Qiqihar Medical University 2015; (9): 1353-5.

158. Dai CS. Research on college students’ suicide idea status in our country - In some medical colleges and universities as the research sample. Journal of Jiamusi Vocational Institute 2016; (1): 454-5.

159. Gao J, Song GQ, Wu NY, Fang GX. Incidence of depression among medical students in a medical university of Anhui. Chinese Rural Health Service Administration 2016; 36(8): 1025-7.

160. Huang YL. A Study on the Correlation among Rumination, Self-esteem and Depression in Medical students. Journal of Chengdu Medical College 2016; 11(4).

161. Jiang HC, Mao Y, Li YQ. Frontier province medical students sleep quality and anxiety and depression correlation analysis. Chinese Journal of Public Health Management 2016; 32(3): 305-8.

162. Lv SX, Tian X, Sun HW. The Mediating Effect of Dispositional Hope between Suicidal Ideation and Depression Emotion in Medical College Students. China Journal of Health Psychology 2016; 24(6): 922-4,5.

163. Qian YK, Li TZ, Wu D, Wu XJ. Relationship among Depression and Life events, Coping styles of Medical students. China Journal of Health Psychology 2016; 24(7): 1079-81.

164. Qiu N, Xu Y. The Relationship between Traditional Chinese Medicine University Students Depression and Traditional Chinese Medicine (TCM) Constitution China Journal of Health Psychology 2016; 24(4): 614-7,8.

165. Sun WW, Liu L, Zhao ZX, Zhu W. Research on the Psychological Anxiety and Its Influencing Factors of Medical College Students (in Chinese). Journal of Qiqihar Medical University 2016; 37(30): 3818-9.

166. Wu YP, Xu Y, Zhu HY. The Relevant Research on Medical College of Traditional Chinese Medicine Students Personality and Depression. China Journal of Health Psychology 2016; 24(2): 301-4.

167. Chen H, Li J, Zhu YM, Zainapu T, Chen N, Tao XJ. The relationship between anxiety, depression and sleep quality of students in a medical university (in Chinese). Journal of Ningxia Medical University 2017; 39(8): 913-6.

168. Dai RY, Gu XH. Analysis of influencing factors of life quality of female medical students with dysmenorrhea in a military medical university (in Chinese). Chinese Journal of Health Care and Medicine 2017; 19(05): 433-5.

169. Fen FL, Li M, Li RF. Mediating effect of feeling of inadequacy on relationship between self consistency and anxiety in medical students. Chinese Journal of Behavioral Medicine and Brain Science 2017; 26(5): 462-6.

170. Li X, Sun X, Wang L, Xue XX. A study on the relationship between big five personality and anxiety symptoms of undergraduates in medical universities and colleges. China Medical Equipment 2017; 14(06): 147-50.

171. LIANG PY, Xiran Y, Jiming H, Zhuangding C, Bin L, shou L. The study of current situation,factors and correlation of learning burnout and anxiety in medical students. Henan Journal of Preventive Medicine 2017; 28(6).

172. Ma X, Shao N, Song XB, He TT, Wan YH. Relationships between childhood abuse and suicide ideation and non-suicidal self injury in medical college students. Chinese Journal of School Doctor 2017; 31(12): 881-3,5.

173. Xu T, Wang Y, Xia YL. Analyses of Medical Student Depression and Anxiety Statusa nd Their Influential Effects. Inner Mongolia Medical Journal 2017; 49(12): 1415-8.

174. Xue L, Linlu Y, Jia'nan G, Ye P, Fuqin X, Junhui K. Epidemiological survey of the depressive symptoms among traditional Chinese medicine college students. China Medical Herald 2017; 14(8): 49-52.

175. Fen FL, Wang CY, Wang YR, Hu SW, Shi HS. Investigation on the status of depression and anxiety among medical college students. Journal of Hebei Medical University 2018; 39(6): 636-9,44.

176. Jiang N, Wang P. Relationship between Life Satisfaction and Depression among Medical Students. Journal of China Medical University 2018; 47(9): 838-41,49.

177. Li XP, Liu W, Yao Z, Hu YH. Investigation and Influence Factor Analysis on the Depression Epidemiology of Medical Students in Jinggangshan University. Journal of Jinggangshan University(Natural Science) 2018; 039(001): P.102-6.

178. Li XX, Zhang XM, Wu FY, et al. Status of Anxiety，Depression and Its Relationship with Type A Behavior Among Medical Students in a Medical College in Changchun City Medicine and Society 2018; 31(2): 55-8,62.

179. Lin F, Zhang LL, Luo ZY, et al. Depression and Its Influencing Factors in Medical Students. China Journal of Health Psychology 2018; 26(4): 622-7.

180. Shi JF, Shi JM, Tian WJ, Wang P, Yu XQ. Investigation on the prevalence of depression among college students and physical identification (in Chinese). Shanxi Journal of Traditional Chinese Medicine 2018; 34(5): 50-2.

181. Wu JT. The status of depression in Medical College Students and its correlation with personality characteristics Journal of Qiqihar Medical University 2018; 0(22).

182. Zhao F, Pan XF, Yang X, Wen Y, Zhao ZM, Yang CX. Prevalence of anxiety symptoms and its correlates among Chinese medical students. Modern Preventive Medicine 2018; 45(22): 4114-8.

183. Zheng CJ. Investigation on the Life Attitude of Medical Students in One Medical University in Wenzhou. Chinese Medical Ethics 2018; 31(6).

184. Ai D, Jiang MM, Wang YQ, Jin YL. Correlation study on the Internet addiction and depression and anxiety among students in a medical college. Journal Of Wannan Medical College 2019; 38(6): 586-8,92.

185. Cao L. The Depressive Symptom and Its Influencing Factors of Undergraduate Freshmen in a Medical School in Chongqing. Medicine and Society 2019; 32(12).

186. Jing， L, Ｒong， G, Yuhui W. Relationships between different forms of social support and suicidal ideation and gender differences analysis among medical college students in Anhui Province. Chinese Journal of Disease Control & Prevention 2019; 23(1): 80-4.

187. Li ZC, Zhang LM, Luo PJ, Qu ZQ. Investigation on Sleep Quality and Anxiety of Undergraduates Majoring in Traditional Chinese Medicine and Traditional Chinese Medicine

Health Preservation Journal of Zhejiang Chinese Medical University 2019; 43(7): 686-9.

188. Lin X, Zhang L, Li FY. A study on the relationship between medical students’ depression, self-esteem and optimistic-pessinistic and suicidal attitudes. Chinese Journal of Health Education 2019; 35(6): 488-92.

189. Tang SY, Xiao R, Zhang RX. Materialistic values and depression in medical students: the mediating effect of meaning in life. Journal of Southern Medical University 2019; 39(4): 495-9.

190. Wang Z. Impact of school bullying victimization and social supports on anxiety symptoms among medical college student. Chinese Journal of School Health 2019; 0(2).

191. Xiong L, Wan F, Huang M, et al. nvestigation and analysis on depression status of medical students in higher vocational college. Chongqing Medicine 2019; 48(7): 1185-7.

192. Xiujuan Z, Fengxue Z, Li Z, Sha L, Youzhong A. Anxiety and depression state and their associated factors of eight-year program medical students at postgraduate stage. Chinese Journal of Medical Education Research 2019; 39(1): 30-4.

193. Chen J. Investigation and study of psychological health of medical students in three universities. The Journal of Practical Medicine 2020; 36(10).

194. Liu NN. Investigation and analysis of multi-grades psychological status of eight-year undergraduates in clinical medicine. Basic & Clinical Medicine 2020; 40(1).

195. Liu X, Zhang YB, Li ZY. Correlation study between time management disposition and anxiety of students in a medical college. Journal of Jining Medical University 2020; 43(1): 68-71.

196. Shao R, He P, Ling B, et al. Prevalence of depression and anxiety and correlations between depression, anxiety, family functioning, social support and coping styles among Chinese medical students. Journal of Southern Medical University 2020; 40(8): 1178-83.

197. Yang XL, Yang CY, Chen PX, Sun XY, Wang Y. Relationship among self-regulatory focus with depression，anxiety symptoms and suicidal risks. Chinese Journal of School Health 2020; 41(9): 1354-7.

198. Zhu HQ, Chen JY, Jiang J, et al. Investigation on depression of medical students Journal of Hainan Medical University 2020; 26(3): 226-30.

**S_3. Sensitivity analysis**

| **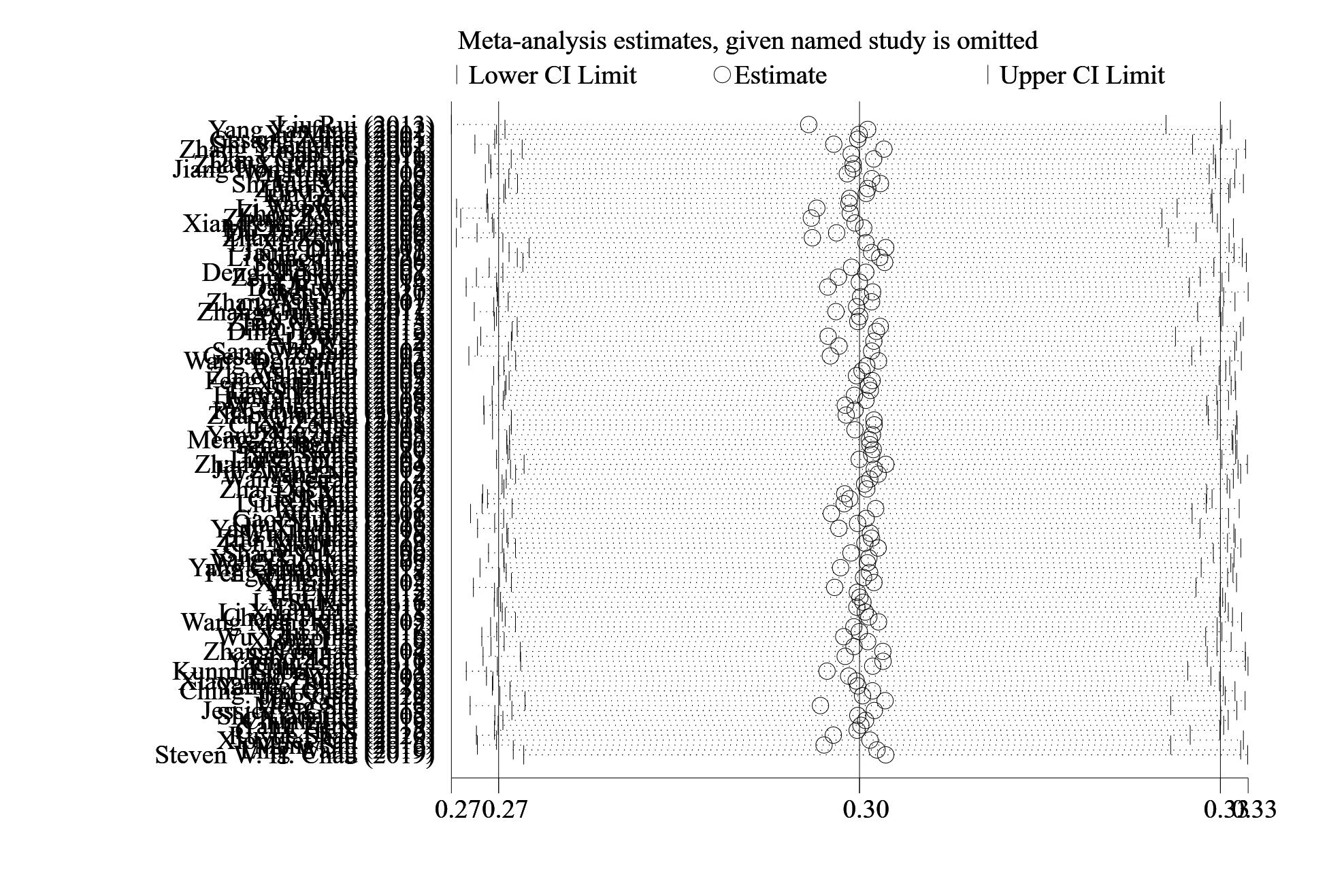** |
| --- |
| **S_3 Fig. 1** Sensitivity analysis of prevalence of depression in Chinese medical students |

| **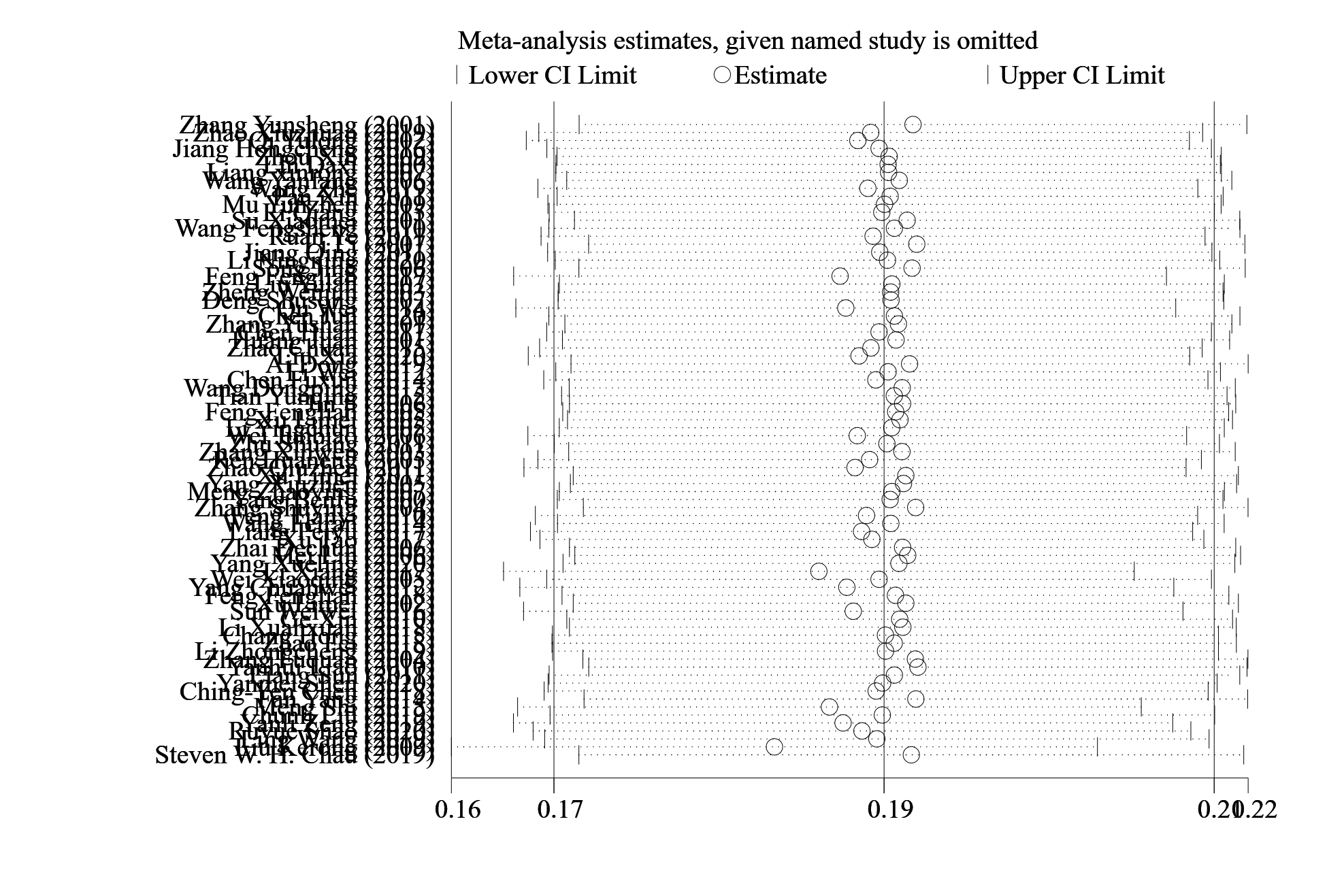** |
| --- |
| **S_3 Fig. 2** Sensitivity analysis of prevalence of anxiety in Chinese medical students |

| **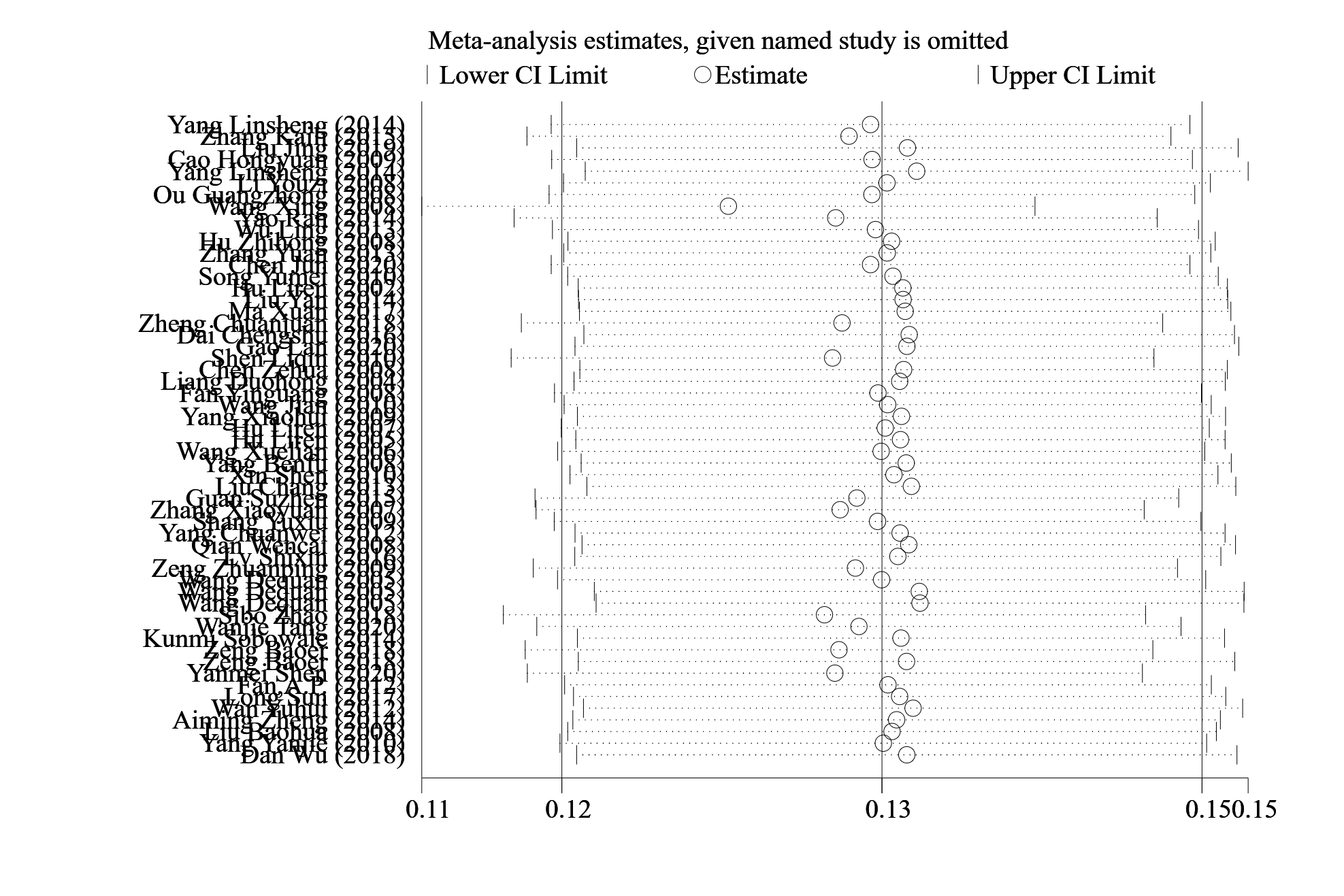** |
| --- |
| **S_3 Fig. 3** Sensitivity analysis of prevalence of suicidal ideation in Chinese medical students |

| **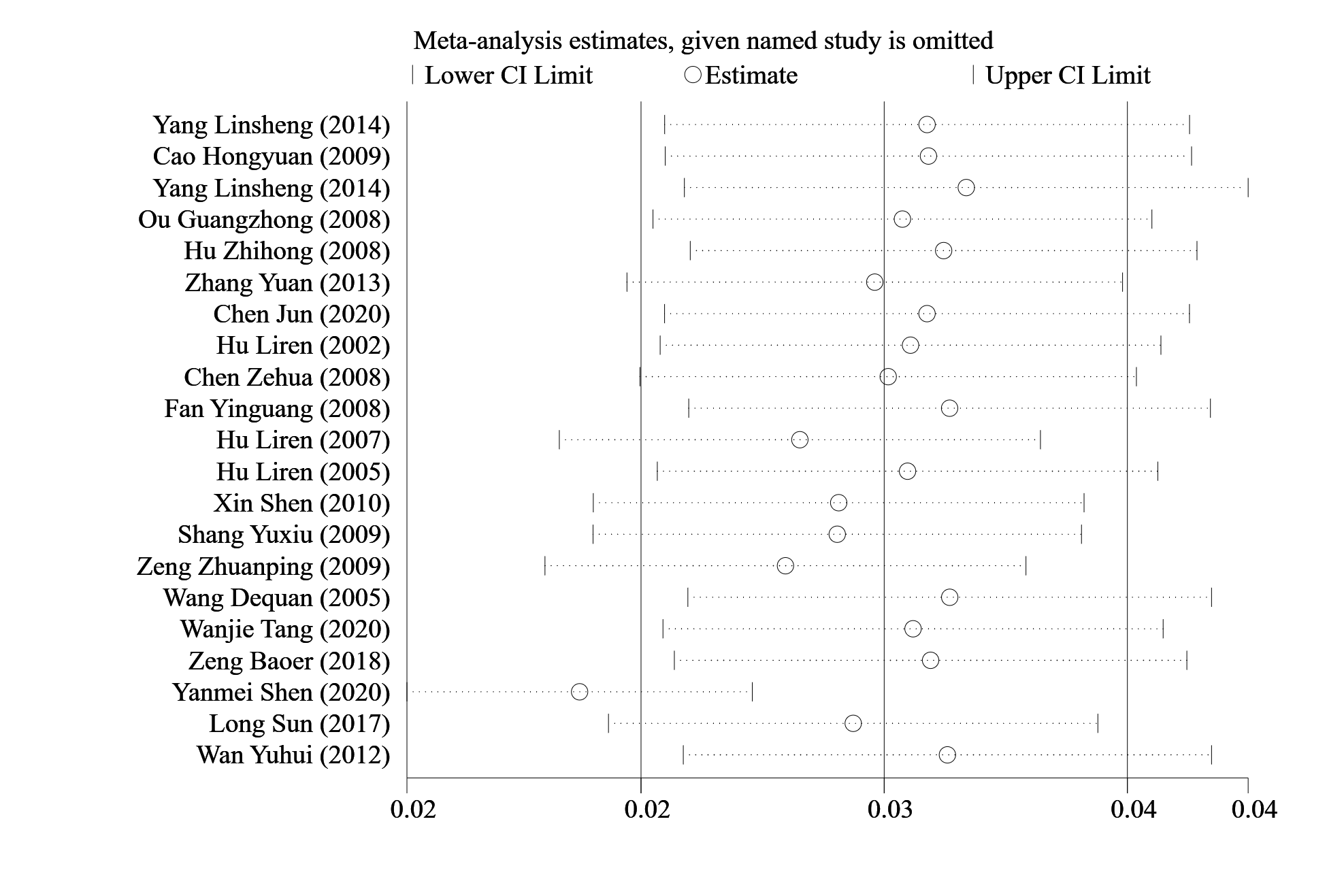** |
| --- |
| **S_3 Fig. 4** Sensitivity analysis of prevalence of suicidal attempts in Chinese medical students |

| **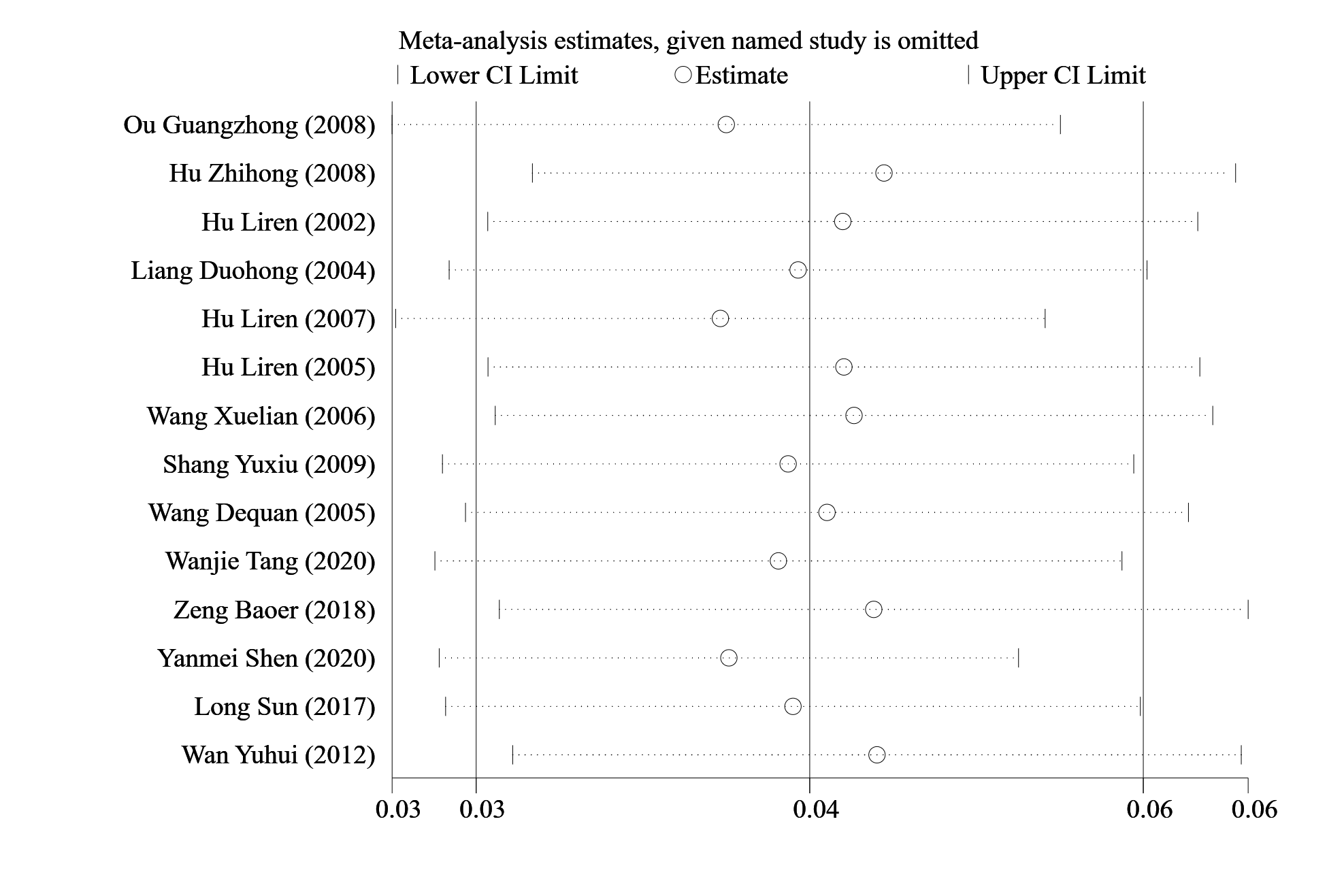** |
| --- |
| **S_3 Fig. 5** Sensitivity analysis of prevalence of suicidal plan in Chinese medical students |

**S_4. Publication bias**

| **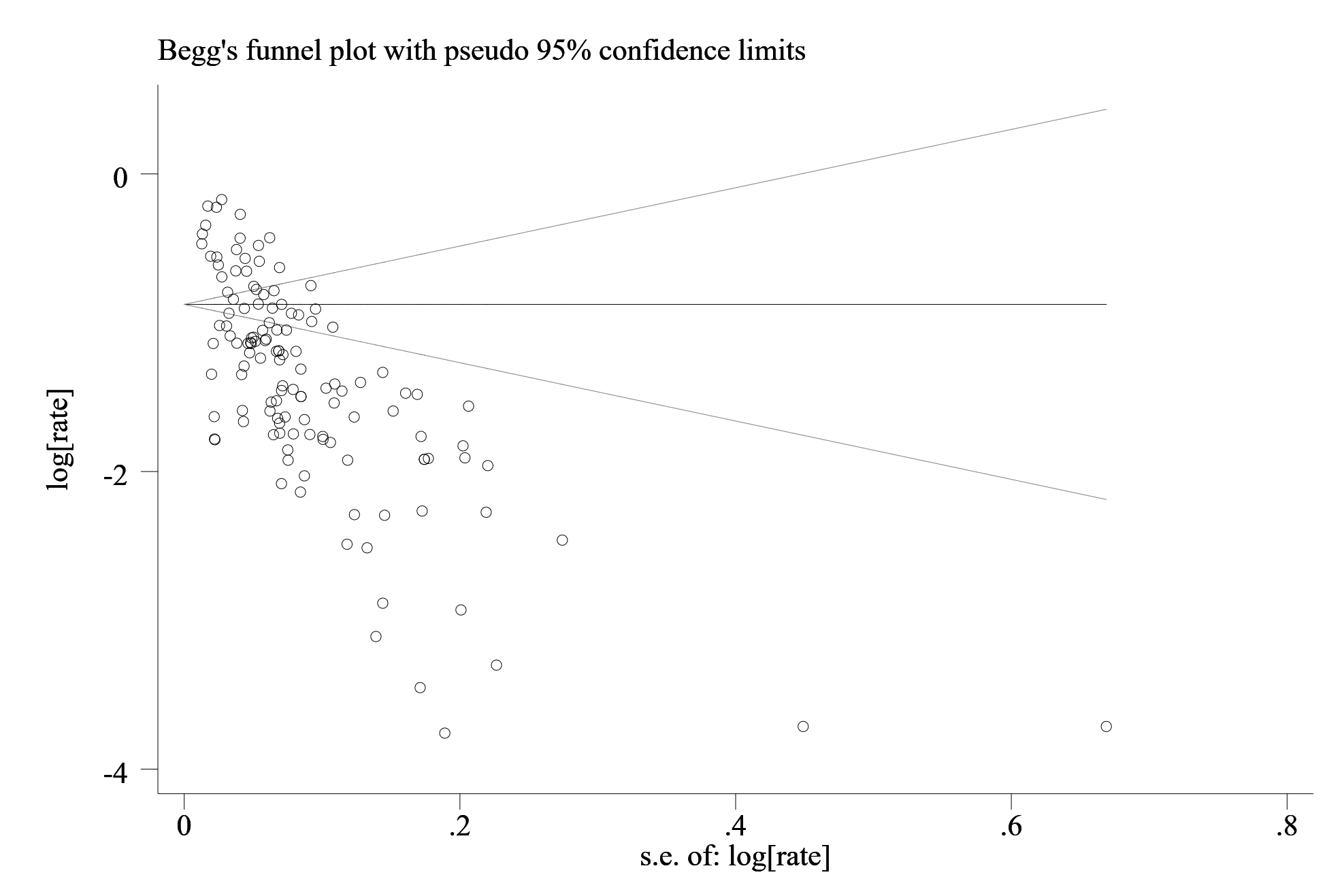** |
| --- |
| **S_4 Fig. 1** Begg’s funnel plot of prevalence of depression in Chinese medical students |

| 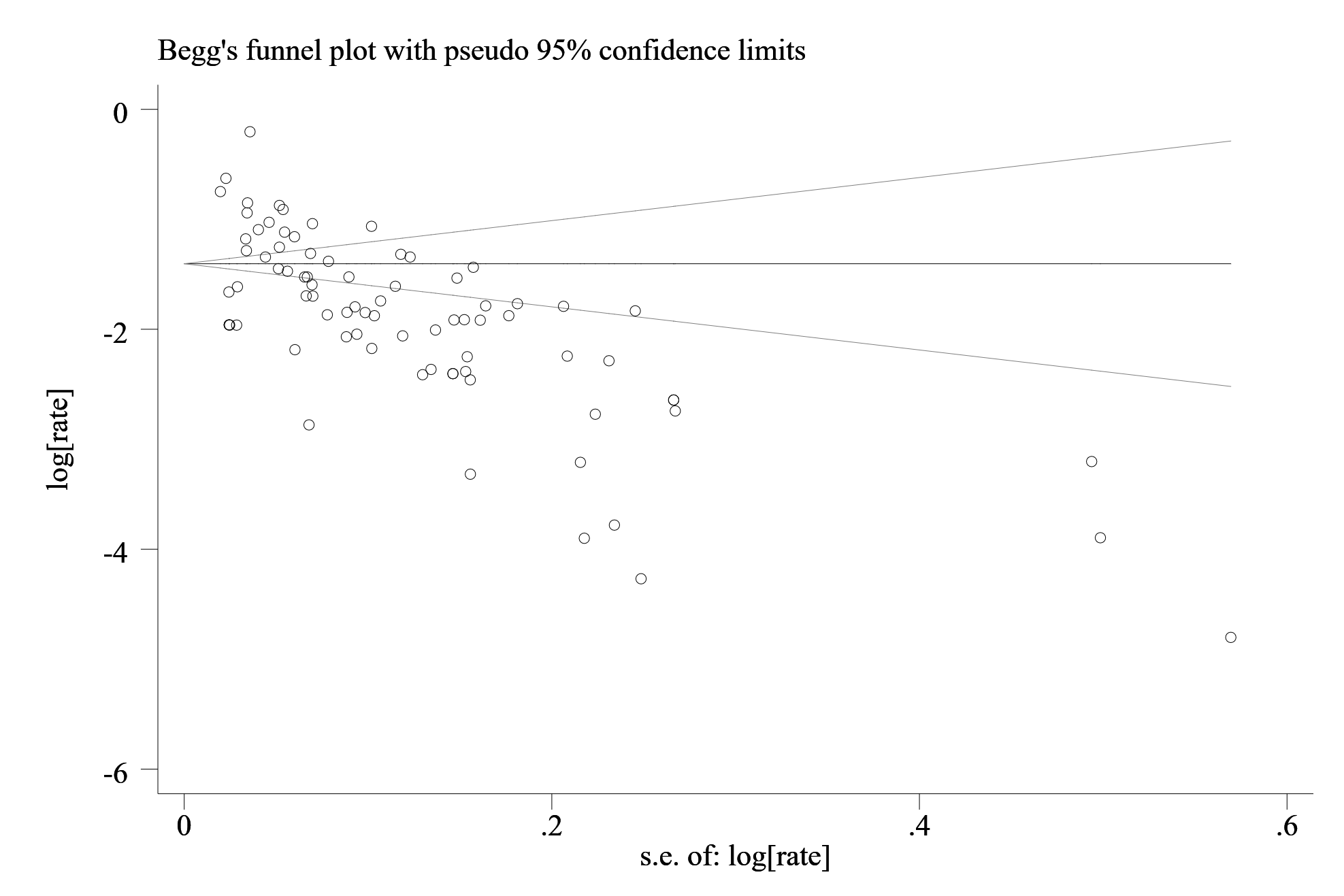 |
| --- |
| **S_4 Fig. 2** Begg’s funnel plot of prevalence of anxiety in Chinese medical students |

| 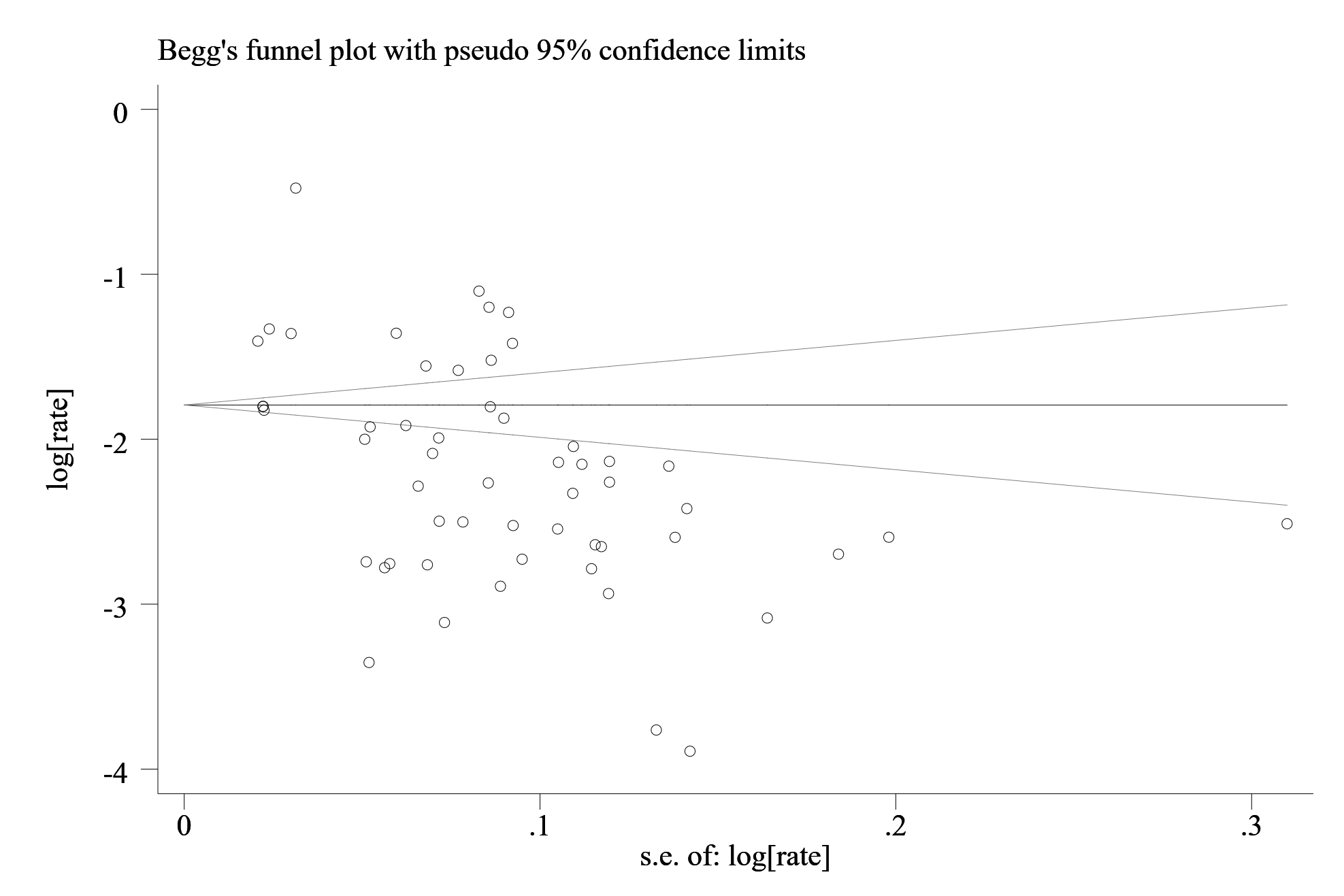 |
| --- |
| **S_4 Fig. 3** Begg’s funnel plot of prevalence of suicidal ideation in Chinese medical students |

| 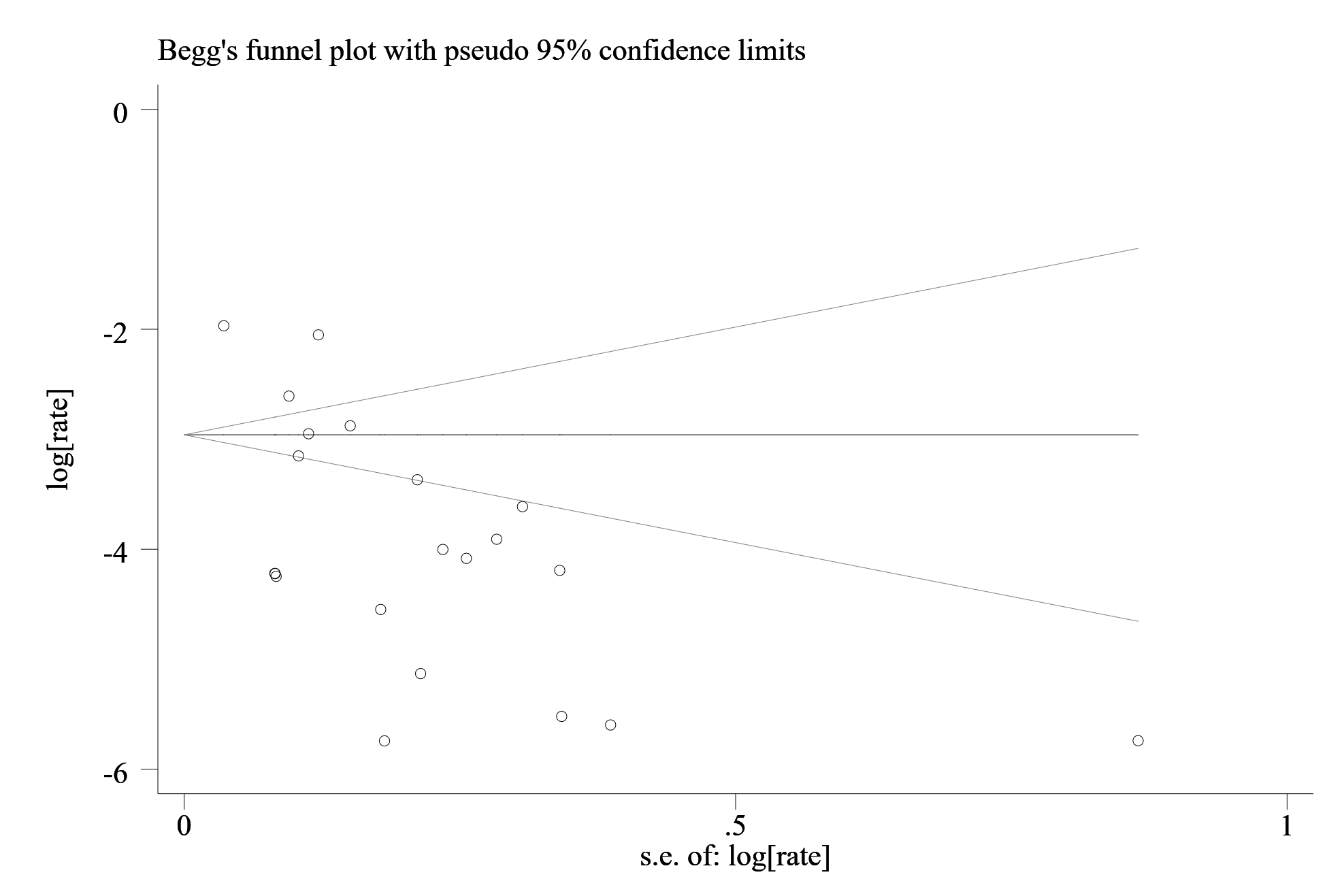 |
| --- |
| **S_4 Fig. 4** Begg’s funnel plot of prevalence of suicidal attempts in Chinese medical students |

| 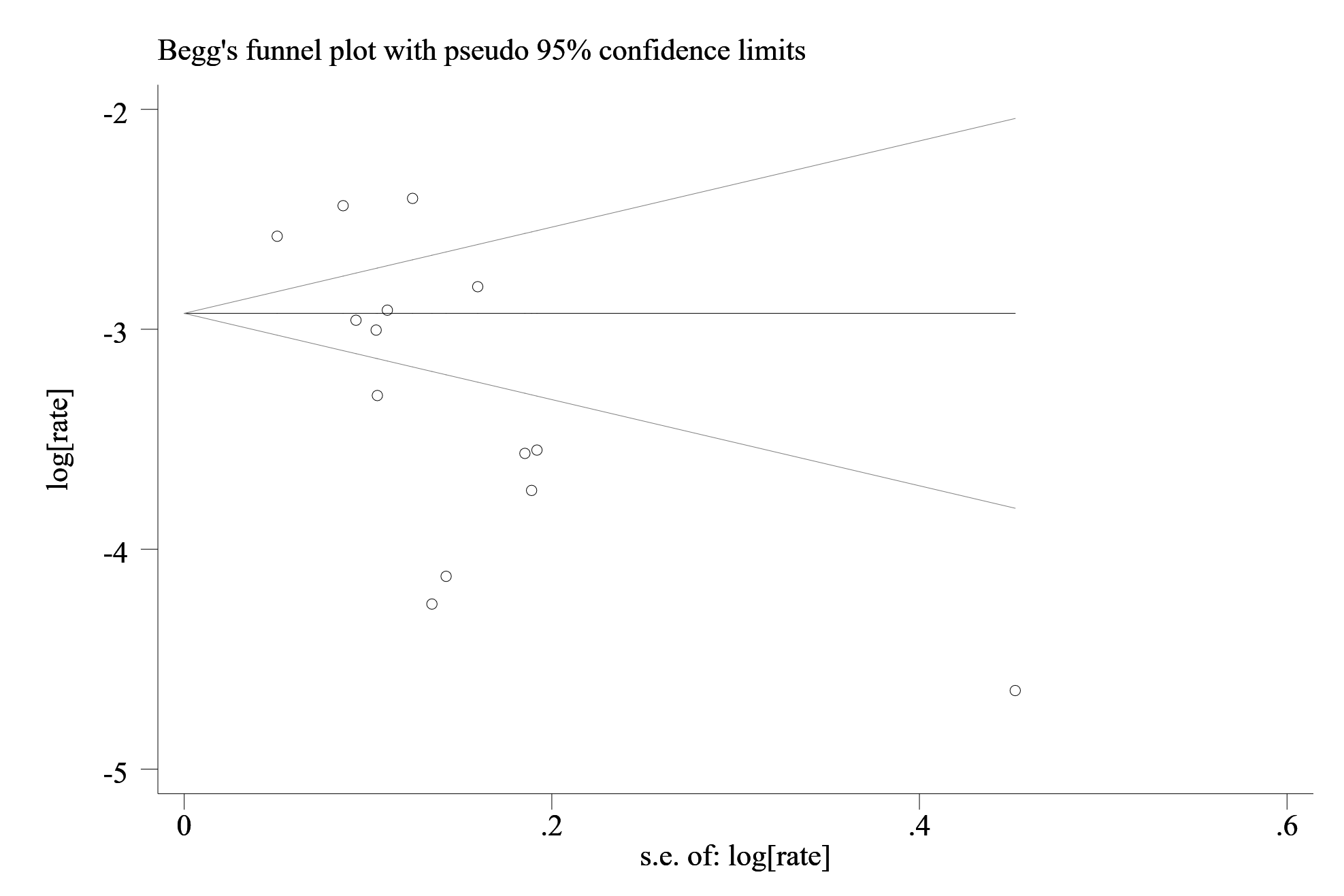 |
| --- |
| **S_4 Fig. 5** Begg’s funnel plot of prevalence of suicidal plan in Chinese medical students |
